# Modulating the dynamics of NFκB and PI3K enhances the ensemble-level TNFR1 signaling mediated apoptotic response

**DOI:** 10.1038/s41540-023-00318-0

**Published:** 2023-11-16

**Authors:** Shubhank Sherekar, Chaitra S. Todankar, Ganesh A. Viswanathan

**Affiliations:** Department of Chemical Engineering, Indian Institute of Technology Bombay Powai, Mumbai, 400076 India

**Keywords:** Dynamic networks, Stochastic modelling, Systems analysis, Numerical simulations

## Abstract

Cell-to-cell variability during TNFα stimulated Tumor Necrosis Factor Receptor 1 (TNFR1) signaling can lead to single-cell level pro-survival and apoptotic responses. This variability stems from the heterogeneity in signal flow through intracellular signaling entities that regulate the balance between these two phenotypes. Using systematic Boolean dynamic modeling of a TNFR1 signaling network, we demonstrate that the signal flow path variability can be modulated to enable cells favour apoptosis. We developed a computationally efficient approach “Boolean Modeling based Prediction of Steady-state probability of Phenotype Reachability (BM-ProSPR)” to accurately predict the network’s ability to settle into different phenotypes. Model analysis juxtaposed with the experimental observations revealed that NFκB and PI3K transient responses guide the XIAP behaviour to coordinate the crucial dynamic cross-talk between the pro-survival and apoptotic arms at the single-cell level. Model predicted the experimental observations that ~31% apoptosis increase can be achieved by arresting Comp1 – IKK^*^ activity which regulates the NFκB and PI3K dynamics. Arresting Comp1 – IKK^*^ activity causes signal flow path re-wiring towards apoptosis without significantly compromising NFκB levels, which govern adequate cell survival. Priming an ensemble of cancerous cells with inhibitors targeting the specific interaction involving Comp1 and IKK^*^ prior to TNFα exposure could enable driving them towards apoptosis.

## Introduction

Apoptotic response is a key outcome of Tumor Necrosis Factor Receptor 1 (TNFR1) signaling triggered by TNFα, a pleiotropic cytokine secreted by variety of immune cells in large quantities in several tissue microenvironment^1^. While normal cells maintain a balance between pro-survival and apoptotic phenotypic responses due to TNFα^2,3^, diseased ones such as those of certain cancer tissues favor proliferation by evading apoptosis and the strong host immune response^4,5^. However, counter-intuitively, certain tumors have higher sensitivity to apoptosis than the corresponding normal tissue^6,7^. Cells modulate the dynamic cross-talk between various intracellular entities wired in an underlying molecular machinery for maintaining this balance^8^. A detailed understanding of the causal mechanisms governing these dynamic cross-talks currently remains elusive, especially when inherent cell-to-cell variabilities leading to heterogeneous responses are present. Such an understanding is necessary to identify the targets for interventional therapeutic strategies for tilting the phenotypic balance towards cell-death without adversely affecting the otherwise normal functioning of an ensemble of cells. For instance, a population of malignant cells may be suitably primed to favor TNFR1 signaling mediated apoptotic outcome over a pro-survival response.

Activated NFκB is a key molecular player during TNFR1 signaling due to its ability to directly modulate both pro-survival and apoptotic responses^9–12^. NFκB regulates its own activity via different feedback loops involving IκB^*^ and A20, both of which are transcribed by NFκB^13,14^. A20 not only regulates NFκB but also deubiquitinates RIPK1 and thereby favors apoptosis by curtailing signals to the necroptosis mode of cell-death^15^. On the contrary, pro-survival markers such as PI3K^16^, IKK^*^^17^, cIAP^18^, XIAP^19^, BCL – 2^20^, directly or indirectly inhibit Caspase3, a precursor for cell-death^21,22^. Signal flow through a cell during TNFR1 mediated pro-survival and apoptotic responses is coordinated by the cross-talks between pathways involving these key regulators^23^. Thus, unravelling the causal mechanisms governing the dynamic signaling cross-talk between these pathways while accounting for cell-to-cell variability requires a detailed investigation of the signal flow through the TNFR1 signaling network.

Discrete model of TNFR1 network led to unveiling the checkpoints regulating apoptosis such as ubiquitination in membrane bound Complex – I, which consists of TRADD, RIPK1, TRAF, and cIAP1/2^22,24^. Modulating this checkpoint by arresting the activity of TAK1, which mediates the interaction between IKK^*^ and Complex – I, permits promoting apoptosis by downregulating the NFκB levels^25,26^ and regulating Compex–II, which consists of RIPK1, FADD, and Caspase8^27^. These studies account only for population-averaged behavior and therefore cannot directly offer insights into the cell-to-cell variability driven dynamic cross-talk between pro-survival and apoptosis arms of the network.

Cell-to-cell variability causing an ensemble-level behavior can influence the signal flow through every cell in a population^28–31^. The sources for variability during TNFR1 signaling range from protein abundances^32^ to cell-cycle effects^33^ to heterogeneity in pathway responses^34^. Besides, the correlation between the information exchanged during cross-talk across different signaling pathways can also contribute to the overall network variability^35^. Signaling networks usually have multiple pathways culminating in a phenotype. As a result, cells adopt different signal flow paths for committing to different cell-fates^36^. The sources for variability considered so far do not account for signal flow variability during TNFR1 signaling which is needed to mimic the natural heterogenous cellular response. Therefore, introducing signal flow variability while modeling single-cell level TNFR1 signaling response permits reliable identification of the governing principles behind the phenotypic heterogeneity.

Discrete modeling approaches such as Boolean Dynamic (BD) framework are amenable to study large-scale networks^37^. Petri-net^25^ and the multivalued Boolean model^38^ of TNFR1 signaling network revealed that regulation of NFκB by inhibition of Complex–I and of XIAP, respectively could modulate the cell-fate. The synchronous updating method of BD simulations employed in these studies are inherently deterministic in nature and therefore cannot account for heterogeneity in the phenotypic response, typically observed in an ensemble of cells^39^. Boolean model of TNFR1 network accounting for the inherent stochasticity using general asynchronous (GA) update scheme^40^ enabled distilling out the role of RIPK1 in cell-death^41^. GA scheme, though widely adopted for modeling signaling cross-talk in the presence of heterogeneous responses^41–43^, can generate spurious signal flow paths of the network. Another promising approach is the probabilistic generalization of the BD modeling^44,45^. In these approaches, challenge lies in mimicking the Boolean functions by a continuous probability framework that hinges on the approximation of the logics^44^ and on network partitioning^45^, errors in which can result in severe information loss. An appropriate approach for quantifying a biological network’s phenotype reachability must therefore account for signal flow variability while minimizing spurious signaling paths, which can be achieved by using Random order asynchronous (ROA) update scheme based BD simulations.

The goal of this study is to unravel the causal mechanisms governing the dynamic cross-talk between pro-survival and apoptotic pathways that regulate the cell-to-cell signal flow variability guided TNFR1 signaling mediated heterogeneous cell-death response. In order to achieve this goal, we develop a Boolean dynamic (BD) model of a TNFR1 network that accounts for the cell-to-cell signal flow variability via the ROA updating scheme. We corroborate the model predictions with the experimental apoptotic phenotypic outcome exhibited by TNFα stimulated U937 and Jurkat-T cells, which respectively are pro-monocytic lymphoma and T-lymphocyte cell lines. We propose a Boolean Modeling based Prediction of Steady-state probability of Phenotype Reachability (BM-ProSPR) algorithm for systematic analysis of the BD model and also reliable quantitation of the network’s reachability to multiple phenotypes. We show that TAK1 inhibition can enable phenotype switching from pro-survival to apoptosis in the model cell lines considered. In particular, we show that, in the presence of cell-to-cell variability, TAK1 inhibition modulates the dynamic signaling cross-talk involving NFκB and XIAP to regulate apoptosis.

## Results

### TNFα induced ensemble-level apoptotic response

**Fig. 1:**
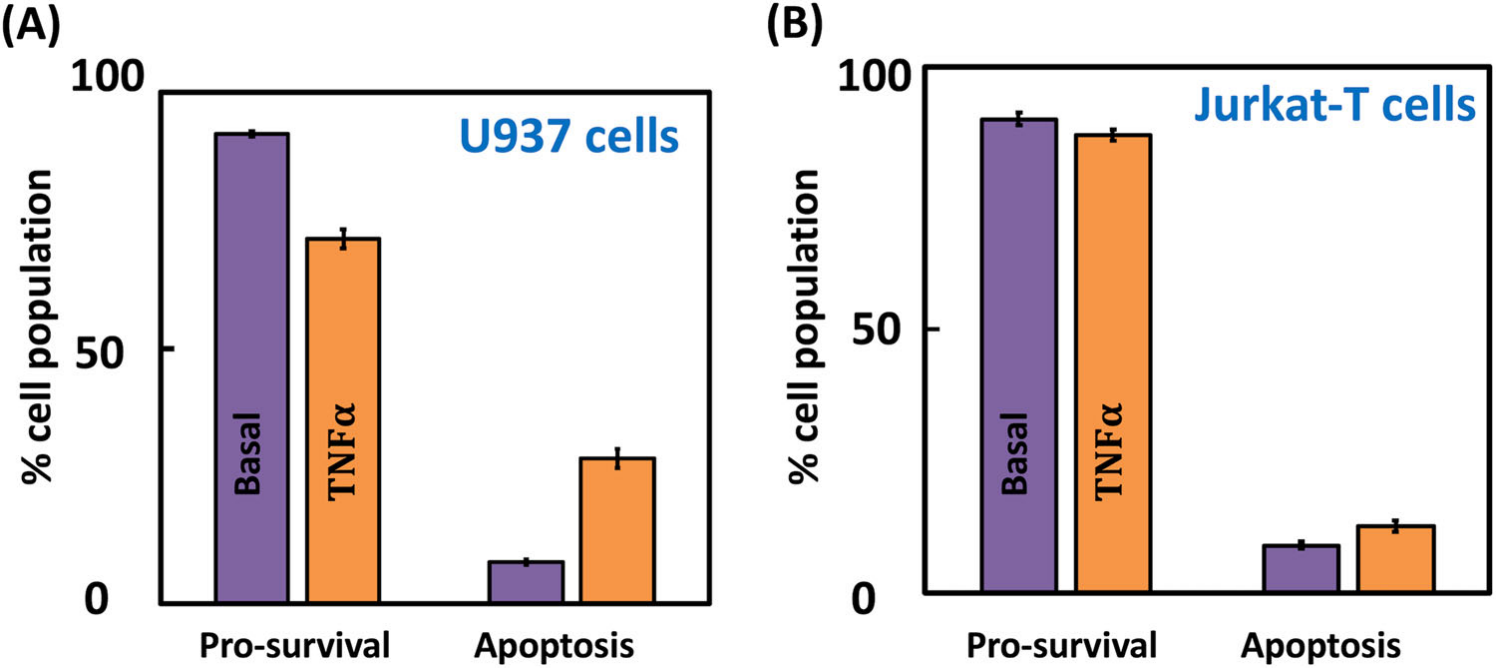
Phenotype responses following TNFα stimulation. Steady-state ${\rm{TNF}}{\rm{\alpha }}$ mediated pro-survival and apoptosis responses in **A** U937 and **B** Jurkat-T cells for basal (no stimulation) and 100 ng/ml ${\rm{TNF}}{\rm{\alpha }}$ stimulation cases. The four-quadrant plot capturing different U937 and Jurkat-T cell states under these conditions are in Supplementary Fig. 1. Error bars represent mean ± standard deviation across three independent replicates.

We consider TNFα driven ensemble-level apoptotic response in U937 and Jurkat-T model cell lines. We experimentally measured the steady-state percentage of cells resulting in apoptosis (Fig. 1) following exposure to 100 ng/ml TNFα (Methods and Supplementary Note 1.1). (The four-quadrant plots tracking different U937 and Jurkat-T cell states are in Supplementary Fig. 1.) While ~28% of the U937 population culminated in apoptosis, only ~13% of Jurkat-T cells exhibited cell-death (Fig. 1). Both U937 and Jurkat-T cells exhibited insignificant TNFα stimulated necrosis population (Supplementary Fig. 1, quadrant Q1)). Hence, unless otherwise stated explicitly, we restrict the definition of cell-death to apoptosis only. This indicates that the TNFα stimulation increases apoptosis but pro-survival response is dominant in both U937 (~72%) and Jurkat-T cells (~87%). This raises a question as to can these cells be primed to favor the ensemble-level apoptosis outcome in response to TNFα exposure and if so, what the targets are. Targets such as TAK1^27,46^ and SMAC mimetics^47^ to enable population-averaged signaling leading to cell-death response have been identified in literature. However, whether these targets are likely to modulate the ensemble-level signaling to offer improved TNFα mediated apoptosis response remains unclear. Identifying the extent of apoptosis modulation these targets can offer while accounting for the ensemble-level behavior requires a detailed model analysis of the signal flow through the TNFα stimulated TNFR1 signaling network to pro-survival and cell-death phenotypes. Moreover, unravelling the cross-talks between the phenotypes in the presence of the cell-to-cell signal flow variability may enable finding mechanisms suitable for the ensemble-level response.

**Fig. 2:**
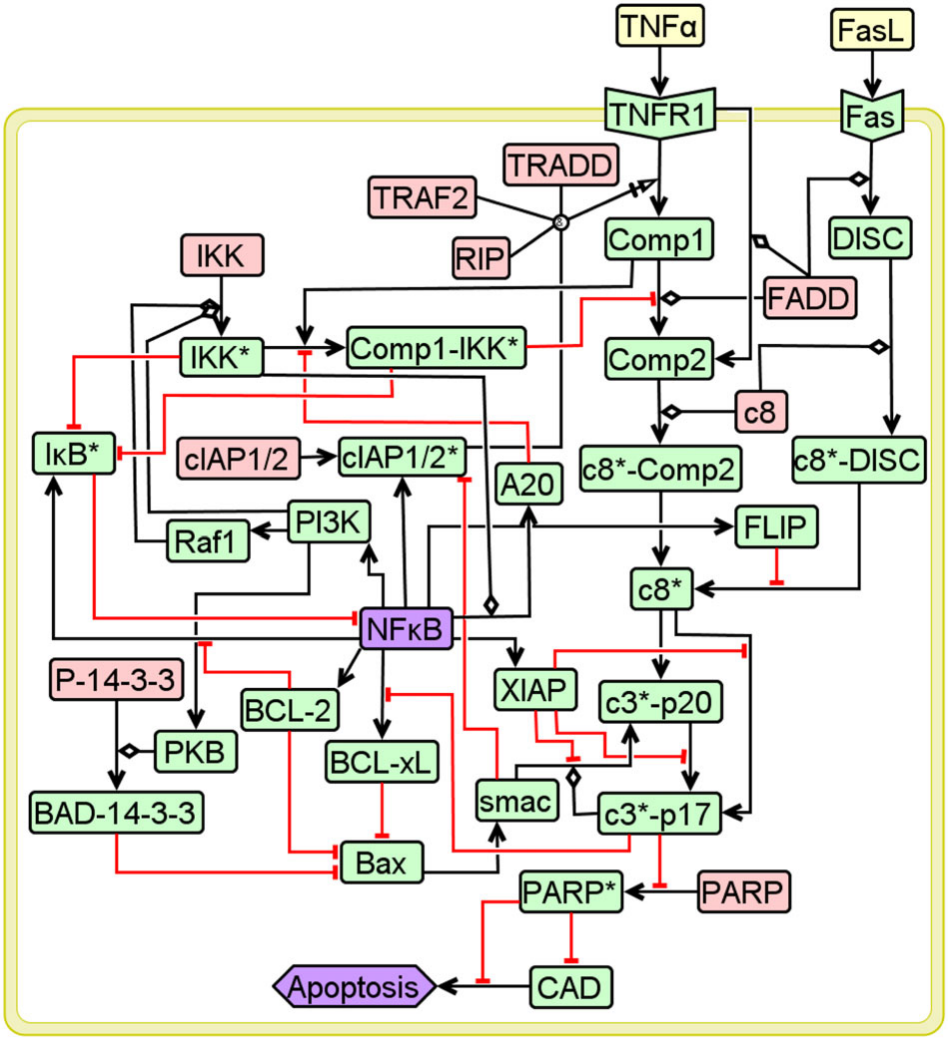
TNFR1 signaling network. Entities in pink, yellow, green and purple, respectively represent housekeeping, input, signaling and output nodes. Black arrows and red hammers capture the activation and inhibitory interactions. Detailed description of the network along with the node-specific Boolean functions are in Supplementary Notes 1.2 and 1.3.

We manually curated a molecular wiring diagram of TNFR1 signaling originating from TNFα and leading to apoptosis (Fig. 2)^1,36,38,41,48^. Fas Ligand (FasL), a TNF superfamily ligand, too leads to a strong apoptotic response via Fas receptor^49^. Since Fas mediated signaling shares majority of the death signal pathways in TNFR1 network, we considered apoptotic response originating from FasL as well for benchmarking purposes (Fig. 2). Note that since U937 and Jurkat-T cells show insignificant necrosis mode of cell-death, the corresponding signaling pathways were not included in the network. The signaling network consists of *N* = 40 entities and 62 causal interactions connecting them. (A detailed description of the network construction and the nature of the interactions along with a list of entities is in Supplementary Notes 1.2 and 1.3.) We classify these entities into 9 housekeeping (H), 2 input (I), 27 signaling (S) and 2 output (O) nodes (Supplementary Table 1). These 9 nodes classified as housekeeping nodes are constitutively present irrespective of any stimulation and also are present in abundance. Note that the levels of these nodes remain nearly constant under resting conditions in different mammalian tissues (Supplementary Table 1). Signaling nodes transduce signal flow from the input (TNFα or FasL) to output nodes (pro-survival represented by NFκB and Apoptosis).

### Boolean Dynamic model of the TNFR1 signaling network permits stochastic phenotypic response

We quantify the inactive (OFF) or active (ON) forms of a node *i* by the Boolean variable ${v}_{i}=0\ {\rm{or}}\ 1$, respectively. Note that ${v}_{j}=1\ \forall j\in {\rm{H}}$. The network’s eventual response can be either apoptotic, pro-survival or anti-apoptotic phenotype depending upon the different combinations of the active/inactive form of the output nodes (Table 1). In Boolean dynamic (BD) framework, the logic of the causal interactions between the entities specifies the Boolean functions ${f}_{i}$ corresponding to a signaling/output node *i*^37^. For example, the Boolean function capturing the activation of entity c8^*^ by c8^*^–DISC in the absence of FLIP or by c8^*^–Comp2 is specified by

1 $${f}_{{\rm{c}}{8}^{* }}\left(\bar{{\boldsymbol{v}}}\right)=\left.{v}_{{\rm{c}}{8}^{* }-{\rm{Comp}}2}\right|({v}_{{\rm{c}}{8}^{* }-{\rm{DISC}}}\ \& \left( \sim {v}_{{\rm{FLIP}}}\right))$$

where, set $\bar{{\boldsymbol{v}}}=\{{v}_{i},\forall i\}$ captures the instantaneous *state* of the network mimicking that of a cell. Note that in Eq. (1), &, | and ~, respectively represent AND, OR and NOT logic. While $\bar{{\boldsymbol{v}}}$ is in general an unordered set, for ease of representation and analysis, we place them in a specific order: 9H, 2I, 27S, 2O (Supplementary Table 1). Logic adopted for the interactions are incorporated in 29 node-specific Boolean functions corresponding to the signaling/output entities (Supplementary Note 1.3). Logic for every interaction was assigned based on the manually curated information pertaining to the underlying causality, details of which are in Supplementary Note 1.3.

**Table 1:** Phenotypes permitted by the TNFR1 signaling network (Fig. 2) and the conditions that specify them.

Apoptosis NFκB	Inactive (0)	Active (1)
**Inactive (0)**	Anti-apoptotic	Apoptotic
**Active (1)**	Pro-survival	–

Values inside a bracket indicate the Boolean value taken by the output nodes Apoptosis and NFκB.

We first reduce the dimensionality of the network using a partial-Logical steady state analysis (pLSSA)^50^ (Methods; Supplementary Note 1.4). For e.g., ${v}_{{\rm{TNF}}{\rm{\alpha }}}=1$ implies ${v}_{{\rm{TNFR}}1}={v}_{{\rm{Comp}}1}=1$ indicating that Comp1 value is fixed irrespective of the dynamics of the rest of the network (Supplementary Table 3). Under TNFα stimulation conditions, pLSSA led to fixing the Boolean values of 10 signaling nodes, *viz*., TNFR1, Comp1, Comp2, c8^*^–Comp2, Fas, DISC, c8^*^–DISC, c8^*^, cIAP1/2^*^, c3^*^–p20, and thereby resulting in *N*=19 dynamically varying signaling/output entities. Note that the value of these “fixed nodes” (listed in Supplementary Table 3) remains unchanged even if that of the remaining entities vary dynamically.

While several studies in literature have modeled TNFR1 signaling mediated cell-death using BD approaches^10,25,38,41^, these do not account for stochasticity caused by the cell-to-cell signal flow variability. We account for the cell-to-cell variability in signal flow by introducing the stochastic behavior due to interdependency of the nodes at all update steps via the random order asynchronous updating scheme, henceforth referred to as ROA^40,51^. Starting from a randomly chosen initial state ${\bar{{\boldsymbol{v}}}}_{0}$, we perform BD simulations of the network using ROA till a fixed-point attractor (FP) is reached and thereby track the corresponding signal flow path from ${\bar{{\boldsymbol{v}}}}_{0}$ to the FP^37,40^. (Note that in this study we only consider those absorbing states which are FPs.) Values of *v*_Apoptosis_ and *v*_NFκB_ that specify the three phenotypes are in Table 1.

A directed one step state-transition between two consecutive states in a signal flow path is obtained by evaluating the Boolean functions of the dynamically varying entities. An input condition- and Boolean function-specific state transition graph (STG), whose state-space ${\mathbb{R}}$ consists of 2^*N*^ states, is a collection of all directed signal flow paths to various FPs from any ${\bar{{\boldsymbol{v}}}}_{o}$. The choice of random sequence of Boolean function evaluation at every transition introduces the signal flow path variability to reach a FP from an initial state. Thus, reaching an FP via a signal flow path strongly hinges on the permutations that specify this random sequence at every state-transition involved (Methods).

**Fig. 3:**
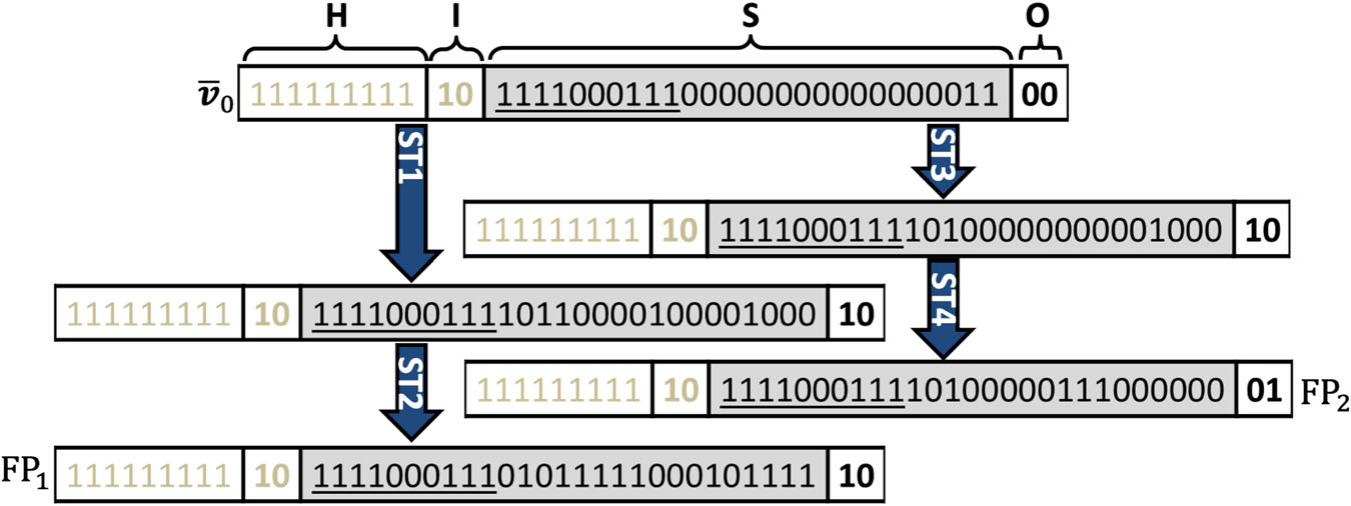
Signal flow paths to fixed points. Absorption of the initial state ${\bar{{\boldsymbol{v}}}}_{0}$ into two fixed points via distinct signal flow paths. The order in which the Boolean values of the entities are placed in a state is housekeeping (H), input (I), signaling (S) and output (O) nodes. Entities corresponding to the underlined values are pLSSA-fixed nodes. Blue arrows indicate one-step transitions with a certain permutation using ROA. Permutation used are in Supplementary Note 2.2.

TNFR1 network (Fig. 2) stimulated with TNFα can lead to *only* two FPs, *viz*., pro-survival FP ${\bar{{\boldsymbol{v}}}}_{{\rm{F}}{{\rm{P}}}_{1}}$ = {9H}{**2I**}{27S}**{2O}**=111111111**10**111100011101011111000101111**10** and apoptotic FP ${\bar{{\boldsymbol{v}}}}_{{\rm{F}}{{\rm{P}}}_{2}}$= 111111111**10**111100011110100000111000000**01** (Methods; Supplementary Note 2.1) where entities corresponding to those underlined digits are pLSSA-fixed. FPs for basal (no stimulation) and for FasL stimulation are in Supplementary Fig. 2. Starting from an initial state ${\bar{{\boldsymbol{v}}}}_{0}$ = 111111111**10**111100011100000000000000011**00**, chosen uniformly randomly, reaching either ${\bar{{\boldsymbol{v}}}}_{{\rm{F}}{{\rm{P}}}_{1}}$ or ${\bar{{\boldsymbol{v}}}}_{{\rm{F}}{{\rm{P}}}_{2}}$ depends upon the signal flow paths dictated by the chosen permutations (Fig. 3 and Supplementary Note 2.2). There could be several such signal flow paths leading to a certain FP from an initial state. Since the stochasticity in phenotypic response originates from signal flow path variability, knowledge of these will enable computing the absorption probability ${\mathcal{p}}^{\bar{\boldsymbol{v}}}_{{\rm{FP}}}$ with which the network would reach a FP starting from a state $\bar{{\boldsymbol{v}}}$. The absorption probability $\mathcal{p}$ due to Markov-chain random walk on the state transition graph is computed by tracking all signal flow paths from an initial state to an attractor. Stimulation condition-specific steady-state probability ${P}_{{ss}}^{{\rm{F}}{{\rm{P}}}_{1}}$ of the TNFR1 network to settle into FP_1_ is given by

2 $${{P}_{ss}^{{{\rm{FP}}}_{1}}=\frac{{\sum }_{\forall {\bar{\boldsymbol{v}}}_{i}\in {\mathbb{R}}}\ {{\mathcal{p}}}_{{{\rm{FP}}}_{1}}^{{\bar{\boldsymbol{v}}}_{i}}}{{\sum }_{\forall {\bar{\boldsymbol{v}}}_{i}\in {\mathbb{R}}}\ 1}}$$

Note that ${P}_{{ss}}^{{\rm{F}}{{\rm{P}}}_{1}}$ specifies the fraction of a population of cells exhibiting the phenotype FP_1_. Computing ${\mathcal{p}}$ requires reliable estimation of the state transition matrix (*M*) whose elements specify the transition probability between any two states in the STG given by

3 $${M}_{{ij}}=\frac{{z}_{{ij}}}{{q}_{\max }}=\frac{{z}_{{ij}}}{\sum _{\forall i,j}{z}_{{ij}}\,}$$

where, *z*_*ij*_ is the number of permutations causing the transition from state *i* to *j*, and *q*_*max*_ is the maximum possible permutations.

Quantifying *M* for TNFα stimulated pLSSA-fixed network (Fig. 2) consisting of ${\mathcal{N}}=19$ dynamically varying entities demands ${2}^{{\mathcal{N}}}\times{\mathcal{N}}{{!}}\times {\mathcal{N}}$ (no. of states × no. of permutations/state × no. of Boolean function evaluations/permutation) = ~1.2 × 10^24^(Unless otherwise stated explicitly, henceforth, TNFR1 network refers to the pLSSA-fixed one.) Note that the number of function evaluations increases exponentially with *N* (Supplementary Fig. 3 and Supplementary Note 2.3). Moreover, every function in turn has embedded in it a complex set of Boolean operations dictated by the network wiring. Therefore, computing *M* even for a reasonably sized network is prohibitively expensive, in spite of the network dimensionality reduction. Approaches for computing *M* so far relied primarily on employing a smaller state space and arbitrarily chosen significantly smaller number of permutations^52^. Employing the entire state space only can offer reliable prediction of the absorption probabilities, and thereby the ability of the network to settle into a phenotype. Thus, there is a clear need for a BD modeling approach using ROA that considers all ${2}^{{\mathcal{N}}}$ states in the STG.

We posit that many chains of permutations could lead to identical signal flow paths, that is, those consisting of the same set of intermediate states in the STG constructed using all ${2}^{{\mathcal{N}}}$ states. We hypothesize that it is possible to judiciously consider only a certain randomly chosen fraction of maximum possible permutations *q*_*max*_ and yet compute a partial STG whose state transition matrix is equivalent to that of a complete STG. Note that the partial STG thus arrived will still contain all ${2}^{{\mathcal{N}}}$ states in the state space. In the next section, we develop and benchmark a systematic algorithm to identify the fraction of ${q}_{\max }$ that can reliably quantitate the (partial) state transition matrix *M*, dimensions of which being (${2}^{{\mathcal{N}}},{2}^{{\mathcal{N}}}$) and subsequently, implement it on TNFR1 network to unravel the effect of signal flow variability on phenotypic heterogeneity.

### Boolean Modeling based Prediction of Steady-state probability of Phenotype Reachability (BM-ProSPR)

**Fig. 4:**
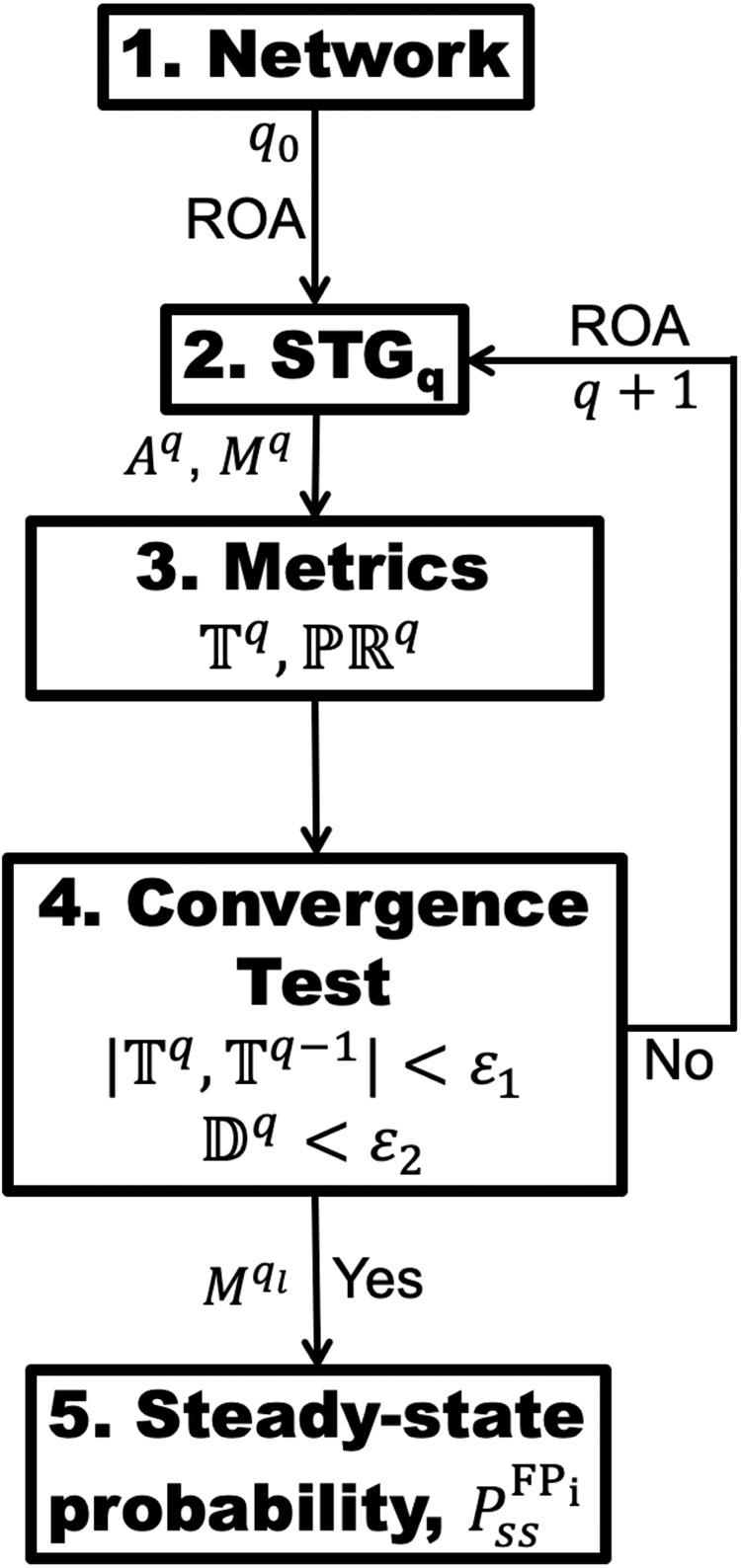
Flow chart elucidating steps involved in BM-ProSPR. While ${\mathbb{T}}$ and ${\mathbb{P}}{\mathbb{R}}$, respectively represent Temporality and PageRank measures, ${\mathbb{D}}$ reflects the discordant PageRank fraction across successive permutations. ${A}^{q}$ and ${M}^{q}$, respectively are the adjacency matrix and state transition matrix corresponding to the partial STG at the ${q}^{{th}}$ permutation. ${\epsilon }_{1}$ and ${\epsilon }_{2}$ are the error thresholds. ${P}_{{ss}}^{{\rm{F}}{{\rm{P}}}_{{\rm{i}}}}$ is the steady-state probability estimated using ${M}^{{q}_{l}}$ at the required minimum number of permutations ${q}_{l}$. ROA update scheme was used in the BD simulations.

The goal of BM-ProSPR algorithm is to find ${P}_{{ss}}^{{\rm{FP}}}$ of reaching different FPs by estimating a reliable state transition matrix *M* using a network-specific minimum number of permutations ${q}_{l}\ll {q}_{\max }$. Flow chart capturing the systematic BM-ProSPR algorithm is in Fig. 4. We first specify the network of interest consisting of *N* nodes, the edges between the entities and the node-specific Boolean functions (Step 1, Fig. 4). (Note that the proposed algorithm can be used for networks with or without pLSSA-fixed nodes.) Next, starting with a null STG (${S}^{0}$) consisting of ${2}^{N}$ isolated states and assuming an initial seed number of permutations $q={q}_{0}\ge 1$, we construct a partial STG ${S}^{{q}_{0}}$ (Step 2, Fig. 4). The steps involved in this construction are in Table 2. After constructing ${S}^{{q}_{0}}$, the associated state transition matrix ${M}^{{q}_{0}}$, where ${M}_{{ij}}^{{q}_{0}}=\frac{{z}_{{ij}}}{{q}_{0}}$ is computed.

**Table 2:** Steps involved in the construction of partial STG (${S}^{q}$) at the ${q}^{{th}}$ permutation.

Steps involved in constructing ${S}^{q}$
a) Choose a state $${\bar{{\boldsymbol{v}}}}_{i}{\mathbb{\in }}{\mathbb{R}}$$.
b) Choose a permutation from ${q}_{\max }=N!$.
c) Starting from ${\bar{{\boldsymbol{v}}}}_{i}$, for the chosen permutation, identify the state ${\bar{{\boldsymbol{v}}}}_{e}$ reached after one BD simulation with ROA (Methods).
d) Update ${S}^{q}$ with the one-step transitions between states ${\bar{{\boldsymbol{v}}}}_{i}$ and ${\bar{{\boldsymbol{v}}}}_{e}$.
e) Repeat steps (b) to (d) for the remaining *q*-1 times while ensuring no redundancy in the permutation chosen in step (b) for a specific state ${\bar{{\boldsymbol{v}}}}_{i}$
f) Repeat steps (b) to (e) for all $\bar{{\boldsymbol{v}}}{\mathbb{\in }}{\mathbb{R}}$.

Finding ${P}_{{ss}}^{{\rm{FP}}}$ hinges on identifying the minimum number of permutations ${q}_{l}\, < \,{q}_{\max }$ such that ${M}^{{q}_{l}}\approx {M}^{{q}_{\max }}$, the state transition matrix of ${S}^{{q}_{\max }}$. This implies that when $q={q}_{l}$ the corresponding partial STG ${S}^{q}$ must have sufficiently evolved to contain enough number of directed links such that ${S}^{q}$ mimics ${S}^{{q}_{\max }}$. We assess the extent of evolution of ${S}^{q}$ by monitoring (i) the connectedness between a pair of states and (ii) the number of permutations causing one-step transitions between a pair of states (Step 3, Fig. 4). First, we consider connectedness by tracking the fractional increment in finding at least one directed link between a pair of states resulting from the update due to the ${q}^{{th}}$ permutation. We quantitate this fractional increment across permutation-driven STG snapshots using a temporality measure^53–55^

4 $${{\mathbb{T}}}^{q}=\frac{1}{q}\mathop{\sum }\limits_{k=1}^{q}\frac{\sum _{\forall i,j{\mathbb{\in }}{\mathbb{R}}}|{a}_{{ij}}^{k-1}-{a}_{{ij}}^{k}|}{\sum _{\forall i,j{\mathbb{\in }}{\mathbb{R}}}{a}_{{ij}}^{k}}$$

where, $${a}_{{ij}}^{k}=\left\{\begin{array}{l}1,{if}\,{at}\,{least}\,{one}\,{link}\,{exists}\,{between}\,i\,{and}\,j\,{in}\,{S}^{k}\\ 0,{otherwise}\,\end{array}\right.$$ and |.| represents *mod*. ${\mathbb{T}}$ is normalized and $0\,<\,{{\mathbb{T}}}^{q}\le 1,\forall q$, with $\mathop{\mathrm{lim}}\nolimits_{q\to {q}_{\max }}{{\mathbb{T}}}^{q}\to 0$, indicating that the STG does not change any further. Next, we quantify the number of permutations leading to a transition between a pair of states by assessing the PageRank (${\mathbb{P}}{\mathbb{R}}$)^56–58^ of all $\bar{{\boldsymbol{v}}}{\mathbb{\in }}{\mathbb{R}}$ (Methods). (Details of the STG evolution quantified by these measures are presented in Supplementary Note 3.1) ${\mathbb{P}}{\mathbb{R}}$ of all states in the null STG is 0. A comparison of the histogram of the ${\mathbb{P}}{\mathbb{R}}$ of all $\bar{{\boldsymbol{v}}}\in {S}^{q}$ and of those in STG ${S}^{q+1}$ for the next permutation can, at best, indicate a change in the topology. However, it does not reveal how addition of a permutation led to a change, if any in the rank order of the states. We track change in this rank order by quantifying the discordance in ${\mathbb{P}}{\mathbb{R}}$ following introduction of a new permutation.

We next introduce conditions on ${\mathbb{T}}$ and ${\mathbb{P}}{\mathbb{R}}$ for identifying ${q}_{l}$ required to ensure sufficiency in the extent of evolution of the partial STG (Step 4, Fig. 4). We set ${q}_{l}$ required for this sufficiency and thereby, for ${M}^{{q}_{l}}\approx {M}^{{q}_{\max }}$ as that *q* which satisfies the conditions

5 $$\left|{{\mathbb{T}}}^{q}-{{\mathbb{T}}}^{q-1}\right| < {\epsilon }_{1}$$

and

6 $${{\mathbb{D}}}^{q}=\frac{\left(1-{\tau }^{q}\right)}{2}=\frac{{D}^{q}}{{C}^{q}+{D}^{q}} < {\epsilon }_{2}$$

where, ${\tau }^{q}={\tau }^{q}{\mathbb{(}}{\mathbb{P}}{{\mathbb{R}}}^{q-1}{\mathbb{,}}{\mathbb{P}}{{\mathbb{R}}}^{q})$ is the Kendall’s-Tau rank correlation^59,60^, and ${D}^{q}$ and ${C}^{q}$, respectively capture the number of pairs having dissimilar and similar rank-order in ${\mathbb{P}}{{\mathbb{R}}}^{q}$ with respect to those in ${\mathbb{P}}{{\mathbb{R}}}^{q-1}\,$(Methods). Therefore, ${C}^{q}+{D}^{q}={2}^{N-1}\left({2}^{N}-1\right)$ (Supplementary Note 3.2). Note that ${{\mathbb{D}}}^{q}$ (Eq. 6) specifies the fraction of pair of states having discordant PageRank across successive permutations. Thus, ${\mathbb{D}}$ offers a rational comparison of ${\mathbb{P}}{\mathbb{R}}$ order achieved in successive permutations. We set the error thresholds ${\epsilon }_{1}$ and ${\epsilon }_{2}$ in Eqs. 5 and 6, respectively by identifying the value at which the absorption probability distribution saturates. Conditions in Eqs. 5 and 6, respectively ensure that the fractional increment of directed links in the STG and topological improvement achieved beyond ${q}_{l}$ are insignificant. For a certain *q*, if Eqs. 5 and 6 are not satisfied, we introduce additional permutation(s) and repeat Steps 2-4 (Fig. 4) until convergence is achieved. After identifying ${q}_{l}$, in Step 5 (Fig. 4), we estimate the steady-state probability ${P}_{{ss}}^{{\rm{F}}{{\rm{P}}}_{{\rm{i}}}}$ (Eq. 2; Methods).

We systematically benchmarked BM-ProSPR using a 6-node apoptosis network of T-cell Large Granular Lymphocyte (T-LGL) cells^42^ permitting two phenotypes (FPs) (Supplementary Note 3.3) and an 8-node network regulating the spinal cord ventrilization in HEK293T^61^ permitting five phenotypes (Supplementary Note 3.4). Based on a sensitivity analysis, we assigned a value of ${1\times 10}^{-4}$ for both ${\epsilon }_{1}$ and ${\epsilon }_{2}$ for which the absorption probability distribution saturates. (Details of the sensitivity analysis used to identify the network-specific error threshold(s) along with an illustration are presented in Supplementary Note 3.5.) The computationally tractable complete STG for these two networks enabled validation of the predictions by BM-ProSPR. The minimum number of permutations required for construction of partial STG that results in accurate steady-state probability for reaching multiple phenotype are 286±65 and 167±2 for the 6-node and 8-node networks, respectively (Supplementary Note 3). Note that the number of permutations required is only a (small) fraction of the total number of possible sequences. In summary, we demonstrated that (a) temporality measure quantifies the extent of evolution of the STG better even in the absence of a complete one, which is typically the case for large networks and thereby making it amenable for analysis any biological system (Supplementary Note 3.3 and 3.4) and (b) BM-ProSPR accurately predicts the absorption probabilities and thereby the steady-state probability to reach multiple phenotypes.

### NFκB and PI3K promote the TNFR1 signaling mediated pro-survival response during TNF*α* stimulation

We next decode the signal flow paths facilitating TNFα mediated apoptotic and pro-survival responses and analyze the transient signaling behavior. After setting housekeeping nodes active, ${v}_{{\rm{TNF}}{\rm{\alpha }}}=1$ and ${v}_{{\rm{FasL}}}=0$, we tracked the Boolean dynamics of the 19 dynamically varying entities of the TNFR1 signaling network (Fig. 2) using BM-ProSPR. (Note that 10 other signaling nodes attained pLSS (see fig. 3)). Only ${q}_{l}=219$ (out of $19!= \sim 1.2\times {10}^{17}$) permutations were sufficient to construct the pSTG with ${2}^{19}=524288$ states for the reliable estimation of the steady-state probability of reaching the pro-survival (${\bar{{\boldsymbol{v}}}}_{{\rm{F}}{{\rm{P}}}_{1}}$) and apoptotic (${\bar{{\boldsymbol{v}}}}_{{\rm{F}}{{\rm{P}}}_{2}}$) attractors (Fig. 3). (BM-ProSPR implementation, the evolution of the pSTG and associated details are discussed in Supplementary Note 4.1) An implementation of BM-ProSPR algorithm on a configuration model random network resulted in the minimum number of permutations for constructing pSTG similar to that obtained for the TNFR1 network (Supplementary Note 4.2). This suggests that the BM-ProSPR approach for construction pSTG could be extended to any network, in general. Since the pSTG corresponding to ${\rm{TNF}}{\rm{\alpha }}$ stimulation condition consists of only non-terminating strongly connected components (SCCs), TNFR1 network (Fig. 2) does not permit cyclic attractors^40^. Moreover, this pSTG is dominated by a single SCC consisting of ~18.9±0.2% states suggesting its importance in the network dynamics. A rational comparison of the pSTG phenotype reachability predictions with experimental measurements on U937 and Jurkat-T cell lines requires incorporation of cell line specific information into the BD model analysis. In these model cell lines, under resting conditions, ${\rm{NF}}{\rm{\kappa }}{\rm{B}}$ is bound to ${\rm{I}}{\rm{\kappa }}{{\rm{B}}}^{* }$ ^13,62^. Thus, for the rest of this section, we consider only those signal flow paths in the pSTG that originate from 131072 states with ${v}_{{\rm{NF}}{\rm{\kappa }}{\rm{B}}}=0$ and ${v}_{{\rm{I}}{\rm{\kappa }}{{\rm{B}}}^{* }}=1$.

The overall dynamical behavior of the TNFR1 signaling network (Fig. 2) is governed by the transient response of the nodes that it consists of. We consider the transient response of ${\rm{NF}}{\rm{\kappa }}{\rm{B}}$, ${\rm{I}}{\rm{\kappa }}{{\rm{B}}}^{* }$, PI3K and ${\rm{c}}{3}^{* }-{\rm{p}}17$. While ${\rm{NF}}{\rm{\kappa }}{\rm{B}}$ and ${\rm{c}}{3}^{* }-{\rm{p}}17$ are respectively direct regulators of the pro-survival and apoptosis phenotypes, PI3K modulates the signal flow to both ${\rm{NF}}{\rm{\kappa }}{\rm{B}}$ and ${\rm{c}}{3}^{* }-{\rm{p}}17$. On the other hand, ${\rm{I}}{\rm{\kappa }}{{\rm{B}}}^{* }$ tightly controls the levels of ${\rm{NF}}{\rm{\kappa }}{\rm{B}}$. Deducing the transient response of the nodes in BD modeling using ROA is non-obvious. A signal flow path in the STG from a certain start state having the ability to reach both attractors mimics the transient behavior in a typical cell. We quantify the transient response of an entity and the embedded variability using an approach of tracking the variation of the node’s activity over the signal flow paths in the STG. An update step, that is, pseudo-time step, causing a one-step transition in the signal flow path qualitatively corresponds the real time. In a signal flow path, after every pseudo-time step, depending on the permutation considered, Boolean simulations force a node (say ${\rm{NF}}{\rm{\kappa }}{\rm{B}}$) to either transition between ON and OFF or maintain at ON/OFF level. Multiple signal flow paths connect a start state ${\bar{{\boldsymbol{v}}}}_{{\boldsymbol{0}}}$ and the two attractors ${\rm{F}}{{\rm{P}}}_{1}$ and ${\rm{F}}{{\rm{P}}}_{2}$. At a certain pseudo-time step *t*, the conditional probability ${\left.{{\mathbb{E}}}_{{\rm{NF}}{\rm{\kappa }}{\rm{B}}}\left(t\right)\right|}_{{\bar{{\boldsymbol{v}}}}_{{\boldsymbol{0}}}}$ of node ${\rm{NF}}{\rm{\kappa }}{\rm{B}}$ to either transition to an active form or remain activated in these multiple signal flow paths (from ${\bar{{\boldsymbol{v}}}}_{0}$) indicates the extent of instantaneous participation of ${\rm{NF}}{\rm{\kappa }}{\rm{B}}$ in the signaling process in a cell with the initial condition ${\bar{{\boldsymbol{v}}}}_{0}$. Thus, ${\left.{{\mathbb{E}}}_{{\rm{NF}}{\rm{\kappa }}{\rm{B}}}\left(t\right)\right|}_{{\bar{{\boldsymbol{v}}}}_{{\boldsymbol{0}}}}$ over all the pseudo-time steps mimics the transient activity of ${\rm{NF}}{\rm{\kappa }}{\rm{B}}$ measured experimentally in a single-cell exposed to ${\rm{TNF}}{\rm{\alpha }}$. We define this conditional probability of finding ${\rm{NF}}{\rm{\kappa }}{\rm{B}}$ being active at an update step *t* as the fractional overall number of permutations (in the ${t}^{{\rm{th}}}$ step) across all signal flow paths wherein the associated one-step transitions either cause an activation of ${\rm{NF}}{\rm{\kappa }}{\rm{B}}$ or maintain it activated. A collection of the pseudo-time step varying conditional probability over all start states in the STG qualitatively captures the ensemble-level ${\rm{NF}}{\rm{\kappa }}{\rm{B}}$ dynamics in a BD framework. Details of the procedure adopted for computing the ensemble-level dynamics of a node and an illustration of the same are respectively in Methods and Supplementary Note 4.3.

**Fig. 5:**
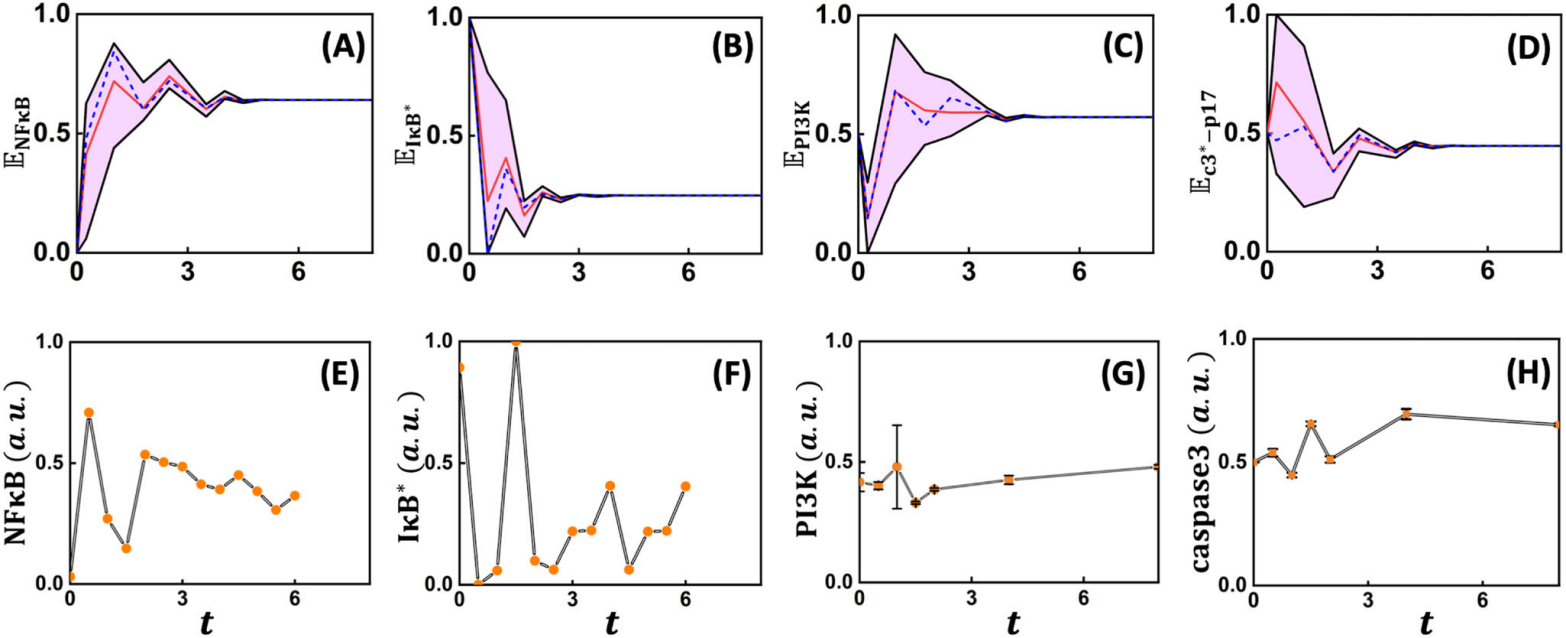
Ensemble level dynamics of key intracellular signaling entities. Model-predicted transients of the conditional probability **A** ${{\mathbb{E}}}_{{\rm{NF}}{\rm{\kappa }}{\rm{B}}}$, **B** ${{\mathbb{E}}}_{{\rm{I}}{\rm{\kappa }}{{\rm{B}}}^{* }}$, **C** ${{\mathbb{E}}}_{{\rm{PI}}3{\rm{K}}}$ and **D** ${{\mathbb{E}}}_{{\rm{c}}{3}^{* }-{\rm{p}}17}$, respectively of the nodes ${\rm{NF}}{\rm{\kappa }}{\rm{B}}$, ${\rm{I}}{\rm{\kappa }}{{\rm{B}}}^{* }$, PI3K, and ${\rm{c}}{3}^{* }-{\rm{p}}17$ being active at an ensemble-level (Methods). Cloud around the population-averaged response (red) captures the range for different transients achieved from various start states of the STG that are capable of reaching both phenotypes. Dynamics of **E** ${\rm{NF}}{\rm{\kappa }}{\rm{B}}$, **F** ${\rm{I}}{\rm{\kappa }}{{\rm{B}}}^{* }$, **G** PI3K/AKT and (H) caspase-3. While ${\rm{NF}}{\rm{\kappa }}{\rm{B}}$ transients are in mouse fibroblast cells treated with ${\rm{TNF}}{\rm{\alpha }}$ ^63^, those of PI3K/AKT and Caspase3 are in ${\rm{TNF}}{\rm{\alpha }}$ stimulated U937 cells^8^. Note that response of ${\rm{c}}{3}^{* }-{\rm{p}}17$ in the network mimics that of Caspase3 in a cell. The update steps in the Boolean simulations are equated to the experimental sampling time by qualitatively aligning the undulations in ${\rm{NF}}{\rm{\kappa }}{\rm{B}}$ transients. Blue dashed line captures the trajectory from a randomly chosen start state. For each of the model cases in (A) to (D), trajectories having the same trend as measurements are presented in Supplementary Fig. 13 in the form of a cloud.

We next compare the BD modeling predicted dynamics of the conditional probability of certain nodes such as ${\rm{NF}}{\rm{\kappa }}{\rm{B}}$, ${\rm{I}}{\rm{\kappa }}{{\rm{B}}}^{* }$, ${\rm{PI}}3{\rm{K}}$ and ${\rm{c}}{3}^{* }-{\rm{p}}17$ being active (Fig. 5A–D) contrasted with corresponding experimental observations (Fig. 5E–H) reported in literature in U937 or equivalent cell lines^8,63^. Even though the update step and the sampling times are not directly comparable, we align the pseudo-time of the BD simulations by contrasting the undulations in the ensemble-averaged ${{\mathbb{E}}}_{{\rm{NF}}{\rm{\kappa }}{\rm{B}}}\left(t\right)$ trajectory with that reported in experimentally measured ${\rm{NF}}{\rm{\kappa }}{\rm{B}}$ transients. We then fixed this update step and real-time mapping for the dynamics of the entities considered. We then juxtapose the model predicted ensemble-level dynamics and the experimentally measured population-averaged transients for ${\rm{NF}}{\rm{\kappa }}{\rm{B}}$, ${\rm{I}}{\rm{\kappa }}{{\rm{B}}}^{* }$, ${\rm{PI}}3{\rm{K}}$ and ${\rm{c}}{3}^{* }-{\rm{p}}17$ in Fig. 5 to contrast the nature of undulations, phase-lag and the steady-state levels. Note that cloud (Fig. 5A–D, pink) around the population-averaged transients (Fig. 5A–D, red) encompasses the dynamics from individual start states of the STG. Comparison of the representative model predicted transients (Fig. 5A–D dashed blue) with the experimentally measured dynamics (Fig. 5E–H) showed that the model predicted the number of crests and troughs exhibited in the ${\rm{NF}}{\rm{\kappa }}{\rm{B}}$ transients. The model simulated ensemble-averaged ${\rm{I}}{\rm{\kappa }}{{\rm{B}}}^{* }$ transient compared with that of ${\rm{NF}}{\rm{\kappa }}{\rm{B}}$ clearly shows these two are out-of-phase with each other (Fig. 5A,B), as observed in the experimental population-averaged measurements (Fig. 5E,F). While the model predicts that both ${\rm{NF}}{\rm{\kappa }}{\rm{B}}$ and ${\rm{I}}{\rm{\kappa }}{{\rm{B}}}^{* }$ settle to higher and lower levels, respectively at the fourth pseudo-time point, measurements show this trend only for ${\rm{NF}}{\rm{\kappa }}{\rm{B}}$. The discrepancy between the model predicted and experimental measurements of ${\rm{I}}{\rm{\kappa }}{{\rm{B}}}^{* }$ transients could be attributed to the inability to account for the strong temporal kinetic control present in the cells in the BD framework. The stimulated TNFR1 network may have a higher chance of favoring a pro-survival response in the initial phase (<3) owing to high probability of ${\rm{NF}}{\rm{\kappa }}{\rm{B}}$ being active (Fig. 5A) along with PI3K concomitantly showing an increasing trend after the first step (Fig. 5C). (Note that PI3K levels in the very early phase (1^st^ pseudo-time point) decreases rapidly as compared to experimental observations. This can be reconciled by considering those states having ${\rm{NF}}{\rm{\kappa }}{\rm{B}}=1$ (see Supplementary Fig. 13B).) In the initial phase, the negative regulators of ${\rm{NF}}{\rm{\kappa }}{\rm{B}}$ could be response for the probability of finding ${\rm{NF}}{\rm{\kappa }}{\rm{B}}$ being active in the individual trajectories to hover around the ensemble-average indicating moderate cell-to-cell variability. On the contrary, PI3K permits wider range for the cell-to-cell signal flow variability in the early phase. Moreover, in the early phase, a large cell-to-cell variation present in ${\rm{c}}{3}^{* }-{\rm{p}}17$ transients indicates that some start states may drive the network towards apoptosis (Fig. 5D). However, eventually, in the late-phase (>6 timesteps) probability that ${\rm{NF}}{\rm{\kappa }}{\rm{B}}$ will be active settles down to ~0.64 for all start states considered (Fig. 5A). This indicates that major fraction (~64%) of the signal flow paths from various start states are likely to favor pro-survival response, which requires ${\rm{NF}}{\rm{\kappa }}{\rm{B}}$ to remain active. Concomitantly the probability of ${\rm{c}}{3}^{* }-{\rm{p}}17$ being active decreases to <0.5 in the late-phase indicating that ${\rm{TNF}}{\rm{\alpha }}$ stimulation is unlikely to result in a strong apoptotic response (Fig. 5D). Transients of ${\rm{PI}}3{\rm{K}}$ and ${\rm{c}}{3}^{* }-{\rm{p}}17$ are out of phase in the very early phase (1^st^ update step). On the contrary, in the late-phase ${{\mathbb{E}}}_{{\rm{c}}{3}^{* }-{\rm{p}}17}(t)$ being in-sync with ${{\mathbb{E}}}_{{\rm{PI}}3{\rm{K}}}(t)$ suggests that the fraction of cells taking apoptotic phenotype maybe regulated by PI3K via Bax (Fig. 5C,D). The decision to attain a phenotypic response is made due to changes in activity of nodes in the initial phase.

**Fig. 6:**
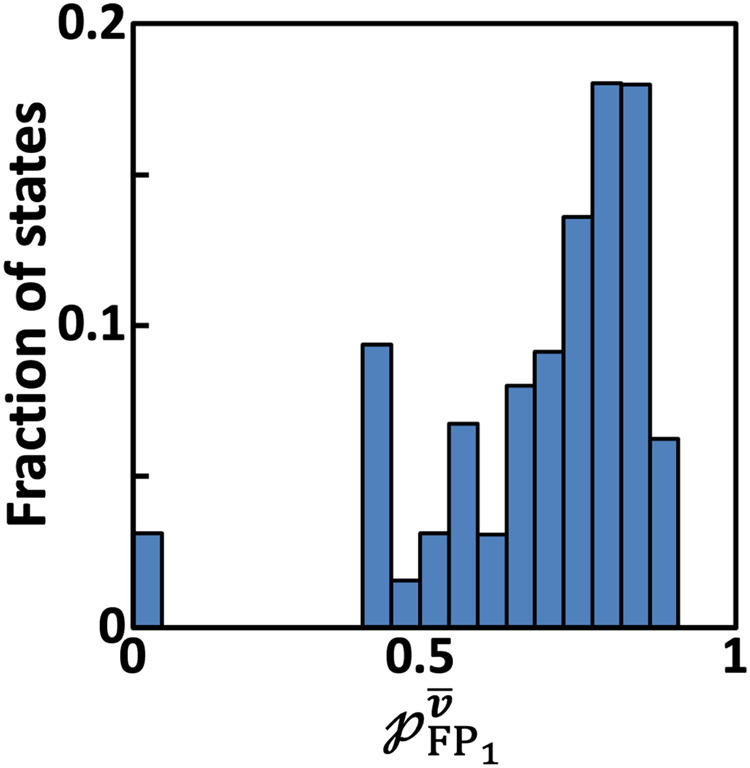
Absorption probability distribution. Histogram of the fraction of states reaching pro-survival FP (${\rm{F}}{{\rm{P}}}_{1}$) with a certain absorption probability ${\mathcal{p}}^{\bar{\boldsymbol{v}}}_{{{\rm{FP}}}_{1}}$.

In order to assess the extent to which ${\rm{TNF}}{\rm{\alpha }}$ mediated pro-survival response is orchestrated by the TNFR1 network (Fig. 2), we computed ${{\mathcal{p}}}_{{{\rm{FP}}}_{1}}^{\bar{\boldsymbol{v}}}=1-{{\mathcal{p}}}_{{{\rm{FP}}}_{2}}^{\bar{\boldsymbol{v}}}\ \forall \bar{{\boldsymbol{v}}}\,\in \,{\mathbb{R}}$ (Methods). Distribution of ${{\mathcal{p}}}_{{{\rm{FP}}}_{1}}^{\bar{\boldsymbol{v}}}$ shows that only 3.125% of the states belonged *only* to the apoptosis basin of attraction ${{\mathbb{B}}}^{{\rm{F}}{{\rm{P}}}_{2}}$ and clearly indicates that a pro-survival response is favoured (Fig. 6). Moreover, there are no states exclusively favoring the pro-survival response. (Note that pro-survival response is favored under no stimulation (basal) conditions as well (Supplementary Fig. 14).) The steady-state probability ${P}_{{ss}}^{{\rm{F}}{{\rm{P}}}_{2}}=1-{P}_{{ss}}^{{\rm{F}}{{\rm{P}}}_{1}}$ (Eq. 2) of the network reaching apoptosis attractor under ${\rm{TNF}}{\rm{\alpha }}$ stimulation and basal conditions respectively are ~0.34 and ~0.04. Note that this trend of pro-survival response being a dominant response is in line with the phenotypic observations for U937 and Jurkat-T cells (Fig. 1). This shows that the dynamics of ${\rm{NF}}{\rm{\kappa }}{\rm{B}}$, PI3K and ${\rm{c}}{3}^{* }-{\rm{p}}17$ being active (Fig. 5) clearly reflects the pro-survival response being the dominant outcome of the ${\rm{TNF}}{\rm{\alpha }}$ stimulated TNFR1 network.

In summary, ${\rm{NF}}{\rm{\kappa }}{\rm{B}}$, PI3K and ${\rm{c}}{3}^{* }-{\rm{p}}17$ transients extracted from the Boolean dynamics contain strong signatures of the ${\rm{TNF}}{\rm{\alpha }}$ stimulated TNFR1 network favoring pro-survival phenotype over cell-death at the single-cell level. This leads to a question as to what governs a cell to favor TNFR1 signaling mediated pro-survival phenotype. Further, can the signal flow through pro-survival pathways and specifically those involving ${\rm{NF}}{\rm{\kappa }}{\rm{B}}$ be modulated to enable phenotype switching from pro-survival to apoptosis at the single-cell level?

### Comp1-IKK* activity reduction enables pro-survival to apoptosis phenotype switching

**Fig. 7:**
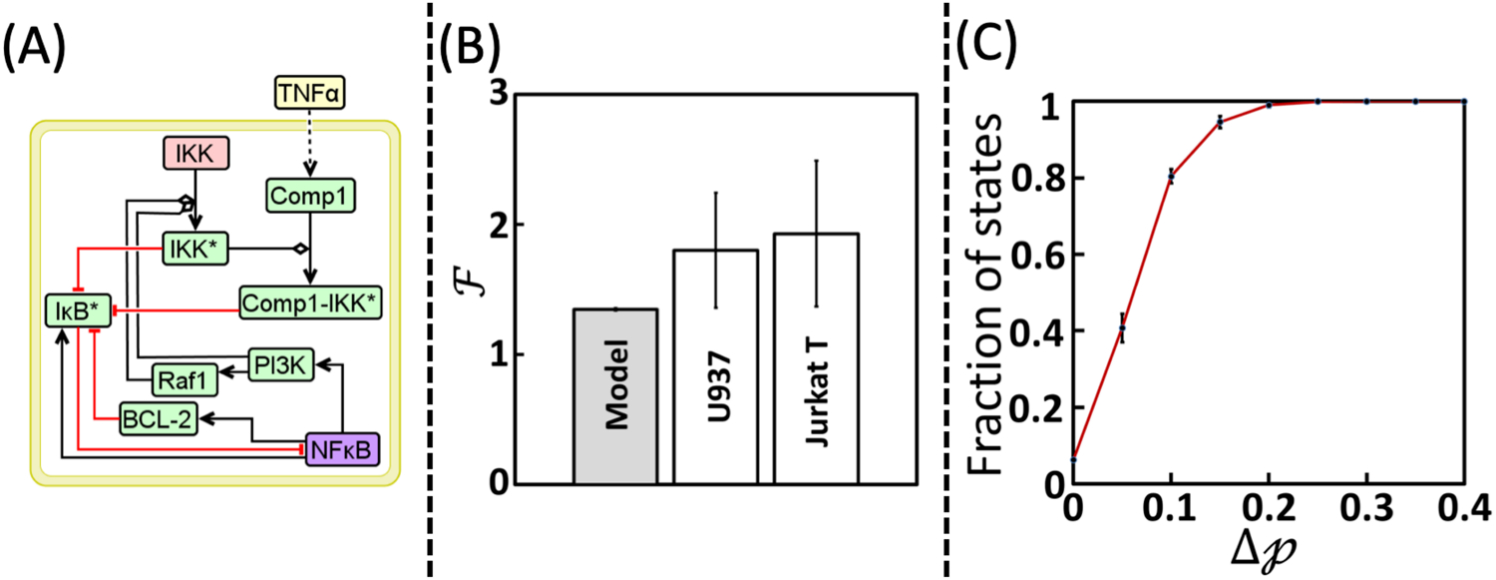
Phenotype switching from pro-survival to apoptosis. **A** Nested loop formed by the 7 key nodes regulating the signaling cross-talk to TNFR1 signaling mediated apoptosis response in the presence of cell-to-cell signal flow variability. Note that IKK (pink box) is a housekeeping node and Comp1 is pLSSA-fixed node. **B** A comparison of model predicted and experimentally measured apoptosis fold change obtained under TAK1 inhibitory conditions. While this inhibitory action in U937 and Jurkat-T cells was achieved using EDHS-206 chemical inhibitor (Methods), correspondingly ${\rm{Comp}}1-{\rm{IK}}{{\rm{K}}}^{* }$ inactivation in the model simulations mimicked TAK1 inhibition. Error bar in the model case captures mean ± standard deviation across 50 STG reconstructions. Error bars in cell line cases capture mean ± standard deviation across three independent replicates. **C** Cumulative distribution of the fraction of states with difference in the absorption probability ($\varDelta {\mathcal{p}}$) in the presence and absence of ${\rm{Comp}}1-{\rm{IK}}{{\rm{K}}}^{* }$. Note that the errorbars on the distribution capture the standard deviation over 50 STG reconstructions. Signal flow paths from those states with ${v}_{{\rm{NF}}{\rm{\kappa }}{\rm{B}}}=0$ and ${v}_{{\rm{I}}{\rm{\kappa }}{{\rm{B}}}^{* }}=1$ were considered to estimate ${\mathcal{F}}$ (Model) and $\varDelta {\mathcal{p}}$ in **B** and **C**, respectively.

Modulation of the signal flow through a pathway can be achieved by either suppressing the activity of a node or the action of an important interaction in such a manner that it does not hamper the otherwise normal functioning of the network. We identify such a target by analyzing the signal flow paths in the pSTG consisting of all the $524288\,(={2}^{19})$ states. We enumerated the frequency of occurrence of a Boolean value (0 or 1) taken by nodes in the 4096 states that exclusively belonged to ${{\mathbb{B}}}^{{\rm{F}}{{\rm{P}}}_{2}}$. In all these states, Boolean value of 7 nodes, *viz*., ${\rm{Comp}}1-{\rm{IK}}{{\rm{K}}}^{* }$, ${\rm{I}}{\rm{\kappa }}{{\rm{B}}}^{* }$, ${\rm{IK}}{{\rm{K}}}^{* }$, ${\rm{PI}}3{\rm{K}}$, ${\rm{NF}}{\rm{\kappa }}{\rm{B}}$, ${\rm{Raf}}1$ and BCL-2 (Table 3A), which are locked in nested loops (Fig. 7A), were the same. Note that the locking of Boolean values of these subset of nodes occurs only in ~0.8% of states. This suggests the strong presence of cell-to-cell signal flow variability while starting from a significant number of states in the STG. Signal flow towards apoptosis phenotype necessitates arresting of ${\rm{NF}}{\rm{\kappa }}{\rm{B}}$ along with other 5 nodes as it inhibits cell-death via multiple pathways such as those involving BCL-2, XIAP, FLIP (Fig. 2). ${\rm{I}}{\rm{\kappa }}{{\rm{B}}}^{* }$ accumulates in the absence of BCL-2 which prevents ${\rm{NF}}{\rm{\kappa }}{\rm{B}}$ activity (as noted in Supplementary Note 1.2). Absence of active PI3K and Raf1 results in lack of activation of ${\rm{IK}}{{\rm{K}}}^{* }$, and therefore the inhibitory action on ${\rm{I}}{\rm{\kappa }}{{\rm{B}}}^{* }$ is absent^64–66^, and thereby preventing ${\rm{NF}}{\rm{\kappa }}{\rm{B}}$ activation causing arrest of signal flow towards pro-survival phenotype (Fig. 1)^67,68^. However, the states having all Boolean value combinations for these seven nodes other than that specified in Table 3A will belong either exclusively to pro-survival basin of attraction ${{\mathbb{B}}}^{{{\rm{FP}}}_{2}}$ or to both ${{\mathbb{B}}}^{{{\rm{FP}}}_{1}}$ and ${{\mathbb{B}}}^{{{\rm{FP}}}_{2}}$. States with 16 (=2^4^) out of the 127 other combinations of the values of these 7 key nodes belong exclusively to the ${{\mathbb{B}}}^{{\rm{F}}{{\rm{P}}}_{1}}$ (Table 3B). (Note that the remaining 111 combinations have $0 \,<\, {\mathcal{p}}_{{\rm{F}}{{\rm{P}}}_{1}}=(1-{\mathcal{p}}_{{\rm{F}}{{\rm{P}}}_{2}})\, < \,1$.) In all these 16 states, ${{\rm{I}}{\rm{\kappa }}{\rm{B}}}^{* }$ takes an inactive form and thereby permitting activation of ${\rm{NF}}{\rm{\kappa }}{\rm{B}}$ leading to pro-survival response. Since inhibitory action on ${\rm{I}}{\rm{\kappa }}{{\rm{B}}}^{* }$ is via either ${\rm{IK}}{{\rm{K}}}^{* }$ or ${\rm{Comp}}1-{\rm{IK}}{{\rm{K}}}^{* }$ or BCL-2 (Fig. 2), BCL-2 taking a Boolean value of 1 (active) along with active ${\rm{NF}}{\rm{\kappa }}{\rm{B}}$ is sufficient to maintain a pro-survival response (Fig. 2). But, modulating BCL-2 alone may not shift the signal towards apoptosis. States belonging exclusively to ${{\mathbb{B}}}^{{\rm{F}}{{\rm{P}}}_{1}}$ can therefore have either active or inactive ${\rm{Comp}}1-{\rm{IK}}{{\rm{K}}}^{* }$. Thus, these 7 nodes locked in the nested loop regulate the cross-talk signaling between the pro-survival and apoptotic responses in the presence of cell-to-cell signal flow variability. We therefore ask a question if tweaking of this nested loop increases the chances of ${\rm{TNF}}{\rm{\alpha }}$ mediated apoptosis in a heterogeneous cell population. This requires modulation in signal flow inside this loop that could enable switching of phenotype, specifically from pro-survival to apoptotic response.

**Table 3:** Combination of Boolean values taken by the 7 key nodes in the states that belong exclusively to either (A) apoptosis or (B) pro-survival attractors.

${\rm{Comp}}1-{\rm{IK}}{{\rm{K}}}^{* }$	${\rm{I}}{\rm{\kappa }}{{\rm{B}}}^{* }$	${\rm{IK}}{{\rm{K}}}^{* }$	${\rm{PI}}3{\rm{K}}$	${\rm{NF}}{\rm{\kappa }}{\rm{B}}$	${\rm{Raf}}1$	${\rm{BCL}}-2$
**Apoptosis attractor** (${\rm{F}}{{\rm{P}}}_{2}$)						
0	1	0	0	0	0	0
**Pro-survival attractor** (${\rm{F}}{{\rm{P}}}_{1}$)						
X	0	X	X	1	X	1

Note that X refers to the value being either 0 or 1.

Switching of pro-survival to apoptotic phenotype can be enabled by either (i) shifting an initial state exclusively in the ${{\mathbb{B}}}^{{\rm{F}}{{\rm{P}}}_{1}}$ to ${{\mathbb{B}}}^{{\rm{F}}{{\rm{P}}}_{2}}$ or (ii) increasing the absorption probability of an initial state to a desired FP. While the former will require simultaneous fixing of the Boolean values of the 7 cross-talk regulating nodes (Table 3A), the latter can be achieved by modulating a single entity. Since tweaking multiple nodes simultaneously could be detrimental especially shutting down ${\rm{NF}}{\rm{\kappa }}{\rm{B}}$ completely, we consider increasing the absorption probability.

We analyzed the signal flow paths culminating into ${\rm{F}}{{\rm{P}}}_{2}$ and identified that knocking-off ${\rm{Comp}}1-{\rm{IK}}{{\rm{K}}}^{* }$ could facilitate tilting the cell-fate towards apoptosis (Supplementary Note 4.6). Since ${\rm{Comp}}1-{\rm{IK}}{{\rm{K}}}^{* }$ formation is mediated by TAK1^69,70^, signal flow through this complex can be modulated by inhibiting TAK1. Use of TAK1 inhibition to promote ${\rm{TNF}}{\rm{\alpha }}$ mediated apoptosis has been demonstrated previously under population-averaged measurements^46^. Moreover, TAK1 inhibition can promote apoptosis either by reducing ${\rm{NF}}{\rm{\kappa }}{\rm{B}}$ activity^71^ or by regulating signaling through RIPK1-FADD-Caspase8 pathway^27^. Since necrotic mode of cell-death seldom occurs in the cell lines considered (Fig. 1 and Supplementary Fig. 1), as noted in earlier section, arresting signal flow through RIPK1-FADD-Caspase8 pathway to promote apoptosis is highly unlikely. Therefore, the ${\rm{NF}}{\rm{\kappa }}{\rm{B}}$ dependent mechanism of promoting ${\rm{TNF}}{\rm{\alpha }}$ mediated cell-death under TAK1 inhibition is in action in Jurkat-T and U937 cells. While these studies have considered population-averaged level approaches, what will be the extent to which modulation of the cross-talk can enable a shift in the ${\rm{TNF}}{\rm{\alpha }}$ mediated apoptosis response, in the presence of cell-to-cell signal flow variability, remains unclear.

In order to unravel this, we created a perturbed TNFR1 network (henceforth referred to as ${\rm{TNFR}}{1}^{\Delta }$) wherein ${\rm{Comp}}1-{\rm{IK}}{{\rm{K}}}^{* }$ node is turned-off by setting ${f}_{{\rm{Comp}}1-{\rm{IK}}{{\rm{K}}}^{* }}=0$ throughout the simulations. BM-ProSPR implemented on ${\rm{TNF}}{\rm{\alpha }}$ stimulated ${\rm{TNFR}}{1}^{\Delta }$ (Supplementary Note 4.1) predicted that the steady-state probability of reaching apoptosis attractor is 0.44. (Note that setting ${f}_{{\rm{Comp}}1-{\rm{IK}}{{\rm{K}}}^{* }}=0$ will halve the number of states with ${\rm{NF}}{\rm{\kappa }}{\rm{B}}=0$ and ${\rm{I}}{\rm{\kappa }}{{\rm{B}}}^{* }=1$ to 65536). In order to validate this prediction, we experimentally measured the apoptosis fraction in U937 and Jurkat-T cells pre-treated with EDHS-206^69^, an inhibitor of TAK1 and subsequently exposed to $100{\rm{ng}}/{\rm{ml}}$ ${\rm{TNF}}{\rm{\alpha }}$ (Methods; Supplementary Note 4.7). Pre-treatment with TAK1 inhibitor for 1 h causes a significant reduction in the ${\rm{Comp}}1-{\rm{IK}}{{\rm{K}}}^{* }$ activity^69^. Note that both Jurkat-T and U937 cells treated with EDHS-206 for extended duration exhibited same viability as that observed for the basal (unstimulated) case (Supplementary Note 4.7). In order to contrast the model predictions with the experimental measurements, we define a fold-change ${\mathcal{F}}$ quantifying the effect of the ${\rm{Comp}}1-{\rm{IK}}{{\rm{K}}}^{* }$ perturbation on apoptosis reachability given by

7 $${\mathcal{F}}{=}\frac{{P}_{{\rm{ss}}}^{{\rm{F}}{{\rm{P}}}_{2}}\left({\rm{TNFR}}{1}^{\Delta }\right)-{P}_{{\rm{ss}}}^{{\rm{F}}{{\rm{P}}}_{2}}\left(\text{basal}\right)}{{P}_{{\rm{ss}}}^{{\rm{F}}{{\rm{P}}}_{2}}\left({\rm{TNFR}}1\right)-{P}_{{\rm{ss}}}^{{\rm{F}}{{\rm{P}}}_{2}}\left(\text{basal}\right)}=\frac{ \% {Apoptosis}\left({\rm{TNFR}}{1}^{\Delta }\right)- \% {Apoptosis}\left({\rm{basal}}\right)}{ \% {Apoptosis}\left({\rm{TNFR}}1\right)- \% {Apoptosis}\left({\rm{basal}}\right)}$$

where, ${P}_{{\rm{ss}}}^{{\rm{F}}{{\rm{P}}}_{2}}\left({\rm{TNFR}}{1}^{\Delta }\right)$ and ${P}_{{\rm{ss}}}^{{\rm{F}}{{\rm{P}}}_{2}}\left({\rm{TNFR}}1\right)$ respectively are the steady-state probabilities of reaching cell-death by ${\rm{TNF}}{\rm{\alpha }}$ stimulated ${\rm{TNFR}}{1}^{\Delta }$ and TNFR1 networks. ${P}_{{\rm{ss}}}^{{\rm{F}}{{\rm{P}}}_{2}}\left(\text{basal}\right)$ is the steady-state probability of the unstimulated TNFR1 network to reach FP_2_. Fold-change ${\mathcal{F}}$ computed using the model simulations and estimated from experimental observations for both Jurkat-T and U937 cells are contrasted in Fig. 7B. The BD model of ${\rm{TNFR}}{1}^{\Delta }$ was able to predict the fold-change reflecting the increased apoptotic response exhibited by both Jurkat-T and U937 cells under reduced ${\rm{Comp}}1-{\rm{IK}}{{\rm{K}}}^{* }$ activity. This shows that inhibiting TAK1 is a promising strategy to tilt the phenotypic response from pro-survival to apoptosis in the presence of cell-to-cell signal flow variability as well. We reason that this predictive ability is due to the effect of ${\rm{Comp}}1-{\rm{IK}}{{\rm{K}}}^{* }$ perturbation on the absorption probabilities, as captured by the $\Delta {\mathcal{p}}={{\mathcal{p}}}_{{{\rm{FP}}}_{2}}^{\bar{\boldsymbol{v}}}({\rm{TNFR}}{1}^{\Delta })-{{\mathcal{p}}}_{{{\rm{FP}}}_{2}}^{\bar{\boldsymbol{v}}}({\rm{TNFR}}1)$ (Fig. 7C). Lack of the activity of ${\rm{Comp}}1-{\rm{IK}}{{\rm{K}}}^{* }$ has resulted in ~88% of the states exhibiting increased ability to settle into apoptosis (Fig. 7C), while the $\Delta {\mathcal{p}}=0$ for the remaining states. Notably, on an average, ~20% of the states showed greater than 10% increase in the probability of settling into apoptosis.

**Fig. 8:**
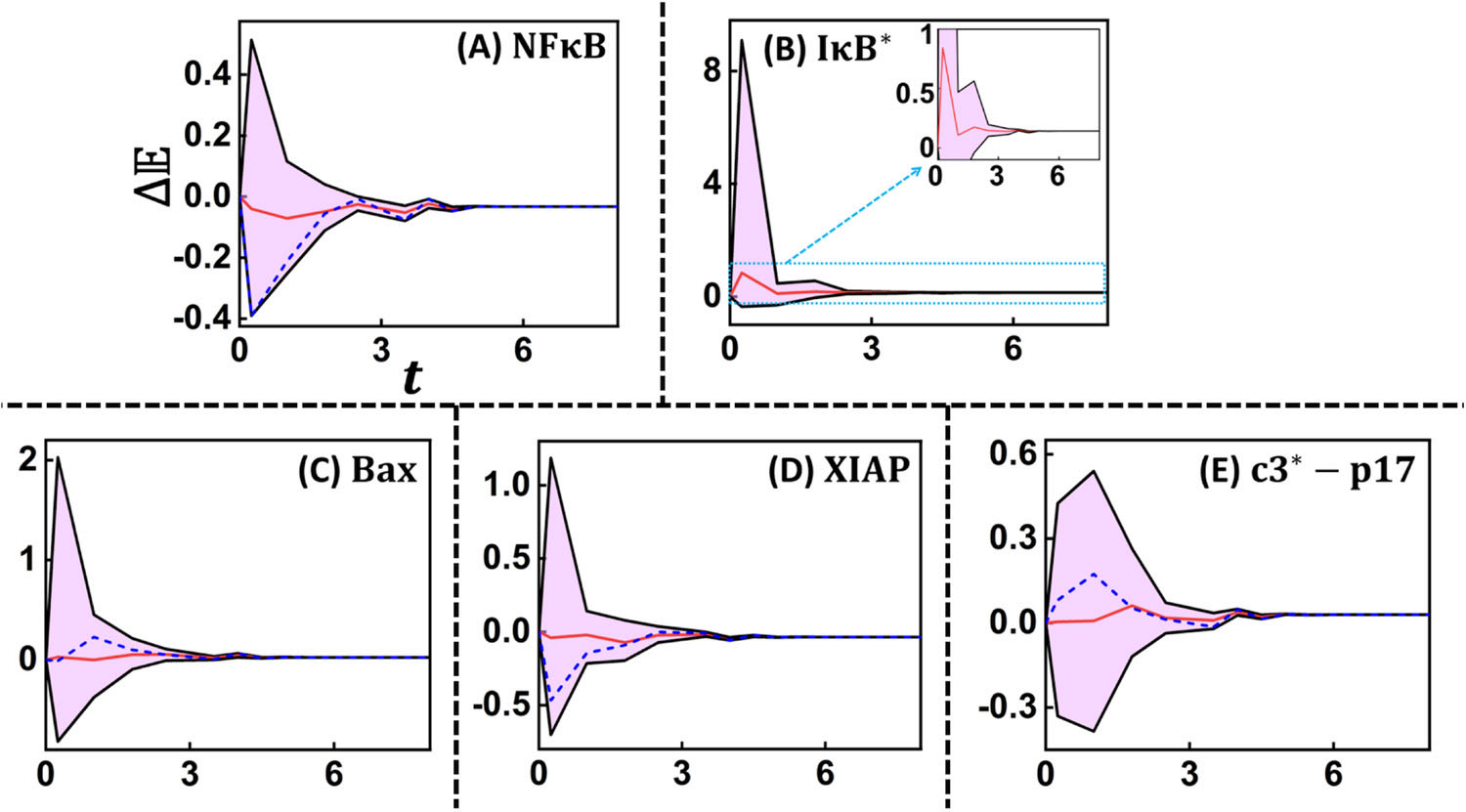
Effect of Comp1 – IKK* inhibition on intracellular signaling entities. Relative change of the transients of **A** ${\rm{NF}}{{\kappa }}{\rm{B}}$, **B** ${\rm{I}}{{\kappa }}{{\rm{B}}}^{* }$, **C** Bax, **D** XIAP and **D** ${\rm{c}}{3}^{* }-{\rm{p}}17$ being active for the case of TNFR1 network with inactive ${\rm{Comp}}1-{\rm{IK}}{{\rm{K}}}^{* }$. Relative change is quantified by ${{\Delta }}{\mathbb{E}}(t)=\left(\right.{{\mathbb{E}}}^{{\rm{TNFR}}{1}^{\Delta}}\left({\rm{t}}\right)-{{\mathbb{E}}}^{{\rm{TNFR}}1}\left({\rm{t}}\right)$)/$\,{{\mathbb{E}}}^{{\rm{TNFR}}1}\left({\rm{t}}\right)$. Pink cloud around the population-averaged trajectory (red) encompasses the ${{\Delta }}{\mathbb{E}}$ from different start states. Blue dashed line captures the trajectory for each of the nodes from the same randomly chosen start state. Inset in **B** captures the zoomed version of a part of the figure for better clarity.

An increase in the ability of a state to settle into apoptosis attractor in ${\rm{TNFR}}{1}^{\Delta }$ is due to the re-wiring of the dynamic path taken by it to reach the attractor, resulting in a shift in the signal flow paths. An illustration of the signal flow path alteration caused by the perturbation is in Supplementary Note 4.6. Such shifts will occur at several one-step transitions in the STG. This can be tracked by following the effect of shutdown of ${\rm{Comp}}1-{\rm{IK}}{{\rm{K}}}^{* }$ on the Boolean dynamics of the key signaling nodes that enable cross-talk between the pro-survival and apoptosis arms of TNFR1 network. For this purpose, we randomly chose a STG reconstruction for the ${\rm{TNF}}{\rm{\alpha }}$ stimulated ${\rm{TNFR}}{1}^{\Delta }$ in which 8282 states exhibited more than 10% increase in absorption probability to reach apoptosis FP. Using these as the start states, we estimated $\Delta {\mathbb{E}}\left(t\right)=({{\mathbb{E}}}^{{\rm{TNFR}}{1}^{\Delta }}\left(t\right)-{{\mathbb{E}}}^{{\rm{TNFR}}1}\left(t\right))/{{\mathbb{E}}}^{{\rm{TNFR}}1}\left(t\right))$ for ${\rm{NF}}{\rm{\kappa }}{\rm{B}}$, ${\rm{I}}{\rm{\kappa }}{{\rm{B}}}^{* }$, BCL-2, XIAP, Bax and ${\rm{c}}{3}^{* }-{\rm{p}}17$ being active following shutdown of ${\rm{Comp}}1-{\rm{IK}}{{\rm{K}}}^{* }$ activity. Inhibition of ${\rm{Comp}}1-{\rm{IK}}{{\rm{K}}}^{* }$ leads to, on an average a ~14% increase in the ability to find ${\rm{I}}{\rm{\kappa }}{{\rm{B}}}^{* }$ being active (Fig. 8B, inset). Concomitantly, in the late-phase, the average relative change in the dynamics of ${\rm{NF}}{\rm{\kappa }}{\rm{B}}$ being active is ~3% but with a large cloud around in the early phase (Fig. 8A). This suggests that the perturbation causes only a small fraction of the signal flow path, on an average, involving ${\rm{NF}}{\rm{\kappa }}{\rm{B}}$ to be shutdown. Thus, the other signaling pathways being active could ensure the other normal functioning of the cell to be preserved intact.

Large cloud around ${\Delta {\mathbb{E}}}_{{\rm{NF}}{{\kappa }}{\rm{B}}}$ in the early phase (t<2) indicates that for those start states for which ${\Delta {\mathbb{E}}}_{{\rm{NF}}{{\kappa }}{\rm{B}}} \,<\, 0$, the signal flows paths having ${\rm{NF}}{{\kappa }}{\rm{B}}$ being active in the case of TAK1 inhibition has significantly reduced compared to that without inhibition. These signal flow paths are perhaps diverted to apoptosis attractor leading to an increased absorption probability. This diversion to apoptotic signaling is mediated primarily by Bax and XIAP. While ${\rm{NF}}{\rm{\kappa }}{\rm{B}}$ directly activates XIAP, it indirectly inhibits Bax via two pathways, which respectively involves BCL-xL and BAD-14-3-3 (Fig. 2). This indirect inhibition could cause the start states having ${\Delta {\mathbb{E}}}_{{\rm{NF}}{{\kappa }}{\rm{B}}}\, < \,0$ to exhibit ${\Delta {\mathbb{E}}}_{{\rm{Bax}}} > 0$ as witnessed by the large cloud above the mean ${\Delta {\mathbb{E}}}_{{\rm{Bax}}}$ in the early phase (Fig. 8C). Since XIAP is activated by ${\rm{NF}}{{\kappa }}{\rm{B}}$, cloud around mean $\Delta {{\mathbb{E}}}_{{\rm{XIAP}}}$ in the early phase is similar to that of $\Delta {{\mathbb{E}}}_{{\rm{NF}}{{\kappa }}{\rm{B}}}$. On the other hand, while Bax can cause an increase in apoptosis by exhibiting a positive influence on ${\rm{c}}{3}^{* }-{\rm{p}}17$, XIAP indirectly reduces cell-death response by inhibiting ${\rm{c}}{3}^{* }-{\rm{p}}17$ in multiple ways including a pathway from Bax via ${\rm{c}}{3}^{* }-{\rm{p}}20$ (Fig. 2). Therefore the cloud around ${\rm{c}}{3}^{* }-{\rm{p}}17$ exhibits a larger relative change for a prolonged time (up to 4^th^ timepoint), indicating significant rewiring of the signal flow paths originating from many start states and leading to ${\rm{c}}{3}^{* }-{\rm{p}}17$ being active, and thereby improving the ability for those states to reach apoptosis. This shows that XIAP could be the prime mediator of the cross-talk between the pro-survival and apoptosis pathways. In fact, this finding is substantiated by the experimental evidence that TAK1 inhibition downregulates XIAP levels in multiple ${\rm{TNF}}{\rm{\alpha }}$ stimulated cells^18,72,73^.

In summary, we show that reduction in the activity of ${\rm{Comp}}1-{\rm{IK}}{{\rm{K}}}^{* }$ can improve the ability of cells to favor apoptotic response over pro-survival phenotype, in the presence of cell-to-cell signal flow variability. The model incorporating the inhibition of ${\rm{Comp}}1-{\rm{IK}}{{\rm{K}}}^{* }$ predicts the apoptotic phenotypic response in Jurkat-T and U937 cells pre-treated with TAK1 inhibitor, which induced the inhibitory conditions. In particular, the reduction in the activity led to modulation of the ensemble-level XIAP mediated dynamic cross-talk between the pro-survival and apoptotic arms of the TNFR1 network to tilt the phenotype towards cell-death.

## Discussion

Dynamic cross-talk regulating the TNFR1 signaling mediated pro-survival and apoptotic phenotypic responses due to ${\rm{TNF}}{\rm{\alpha }}$ stimulation is well-known^2^. Since ${\rm{TNF}}{\rm{\alpha }}$ cytokine is secreted in large quantities by immune cells in a tumor microenvironment^74^, varying extent of dynamic TNFR1 signaling is continuously occurring in a population of cancer cells. Therefore, this dynamic cross-talk regulation during TNFR1 signaling at a single-cell level can be capitalized upon for various cancer therapeutic purposes^7,75,76^. In this study, using a Boolean dynamic model accounting for cell-to-cell signal flow variability juxtaposed with experimental measurements, we demonstrate that ${\rm{NF}}{\rm{\kappa }}{\rm{B}}$ along with BCL-2 and PI3K via XIAP regulates the dynamic cross-talk signaling between TNFR1 network mediated pro-survival and apoptotic responses. The sources for heterogeneity are the variation in the active/inactive state of different nodes and in the multitude of transient signal flow paths, introduced by the random order asynchronous update scheme. We distilled out the dynamic regulation via this cross-talk by systematically analyzing the partial state transition graph (pSTG), constructed using a computationally effective algorithm: ‘Boolean Modeling based Prediction of Steady-state probability of Phenotype Reachability’ (BM-ProSPR). Model analysis predicted the phenotype switching from pro-survival to apoptosis in U937 and Jurkat-T cell lines induced by TAK1 inhibition.

${\rm{TNF}}{\rm{\alpha }}$ activated TNFR1 network model predicts the experimentally observed trend in Jurkat-T and U937 cells that pro-survival response is the favorable outcome under normal conditions. We showed that the transient variation of the key intracellular entities ${\rm{NF}}{\rm{\kappa }}{\rm{B}}$, ${\rm{I}}{\rm{\kappa }}{{\rm{B}}}^{* }$, PI3K, and ${\rm{c}}{3}^{* }-{\rm{p}}17$ being active follows the experimentally observed trend of the corresponding population-averaged trajectories reported in literature^8,63^. This demonstrates that the model developed could mimic the ensemble-level dynamics of the intracellular machinery and therefore captures the phenotypic response. A simultaneous regulation of ${\rm{NF}}{\rm{\kappa }}{\rm{B}}$, PI3K, and BCL-2 dynamics by blocking the ${\rm{Comp}}1-{{\rm{IKK}}}^{* }$ complex can lead to a partial shift of the phenotypic response from pro-survival to apoptosis at single-cell level. This shift can be achieved by preserving many signal flow paths resulting in ${\rm{NF}}{\rm{\kappa }}{\rm{B}}$ being active, especially at the late-phase suggesting the pro-survival signals continue to be preserved to some extent, indicating that essential functions may not have been sacrificed. We substantiated the phenotypic switching using single-cell level experimental measurements in the model cell lines pre-treated with TAK1 inhibitor, a direct modulator^69^ of Comp1 and ${{\rm{IKK}}}^{* }$ complex formation yet preserves the dynamics of essential nodes. TAK1 inhibition favoring the ${\rm{TNF}}{\rm{\alpha }}$ mediated apoptosis response via ${\rm{NF}}{\rm{\kappa }}{\rm{B}}$ has been shown previously^46^. However, we show that TAK1 inhibition additionally modulates the dynamic cross-talk between the ${\rm{TNF}}{\rm{\alpha }}$ mediated pro-survival and apoptotic pathways even when heterogeneity in the signal flow paths are present. A partial switch in the phenotype switching could perhaps be due to the inability of TAK1 inhibitor to shutdown other non-canonical pathways activating ${\rm{NF}}{\rm{\kappa }}{\rm{B}}$^77,78^. While our analysis show only partial shift to apoptosis, a possible approach to enable a significant phenotype switching from pro-survival to apoptotic response is to inhibit LUBAC, which by directly regulating ${{\rm{IKK}}}^{* }$ arrests ${\rm{NF}}{\rm{\kappa }}{\rm{B}}$ activity^79–82^. Although this could be a promising approach, a complete shutdown of ${\rm{NF}}{\rm{\kappa }}{\rm{B}}$ may be undesirable as it can significantly affect the other important and necessary functions of a cell.

A signal flow path from a start state, extracted from the BM-ProSPR constructed pSTG by computing the connectivity (${\mathbb{C}}$) and signal flow path (${\mathbb{P}}$) matrices (Methods), mimics the dynamics of a typical ${\rm{TNF}}{\rm{\alpha }}$ stimulated cell. For unperturbed conditions, simulated transients (of a few nodes) from ~48% of the start states were qualitatively similar to those measured (Supplementary Fig. 13A). Thus, the multitude of signal flow paths originating from such a start state could guide in assimilating the ensemble-level dynamic trends of the signaling nodes in TNFR1 network that either were not or could not be measured experimentally. A comparison of these trends with those computed under other perturbed conditions could offer insights into how signal flow paths can be suitably re-wired for enabling improved phenotype modulation following ${\rm{TNF}}{\rm{\alpha }}$ stimulation without compromising essential cellular functions.

BM-ProSPR employs Temporality^55^ and PageRank^57^ measures to self-learn the extent of evolution of the STG and thereby, aids in identifying the minimum number of permutations required to capture adequate signal flow paths, and the associated variabilities. For small, medium and large networks considered, BM-ProSPR predicted that a (tiny) fraction of the maximum possible permutations is sufficient for constructing a reliable pSTG (Supplementary Figs 6, 7, and 11). For example, only 219 out of $19!\,(=\ \sim\!1.2\times {10}^{17})$ permutations are sufficient to reliably quantify the ${\rm{TNF}}{\rm{\alpha }}$ activated ${\rm{TNFR}}1$ network’s reachability to pro-survival and apoptotic phenotypes (Supplementary Fig. 14). The self-learning nature of BM-ProSPR and the ensuing significantly low computational cost makes it directly amenable to larger networks for which constructing a complete STG is infeasible. Thus, BM-ProSPR makes performing signal flow analysis and thereby distilling out causalities in large biological networks feasible. BM-ProSPR’s ability to reliably construct partial STG for a random configuration model with pre-decided degree, sign and logic distributions suggests that developed algorithm can be applied to study any curated network to model, even non-biological systems. While BM-ProSPR assumes an ON/OFF behavior for a node, the approach can be extended to track cell-to-cell signal flow variability when the entities are multi-valued^83^ causing a steep increase in the state space size.

The ability of BM-ProSPR implementation hinges on starting with a null set consisting of all the states permitted by the Boolean model of the network. Very large biological networks, even after discounting for the partial logical steady-state fixed entities, can have significantly large state space. For such networks, BM-ProSPR too can offer a computational challenge in performing the signal flow analysis. Arriving at strategies for identifying a threshold minimum number of states needed for the reliable construction of the pSTG underlying such very large biological networks could help circumvent this challenge.

## Methods

### Cell culture and reagents

U937 and Jurkat-T cells were procured from the Cell Repository at National Centre for Cell Science (NCCS), Pune, India. It was cultured in RPMI-1640, supplemented with 10% fetal bovine serum (FBS), 2 mmol/l L-glutamine, and 1% antibiotic solution, all procured from HiMedia (Mumbai, India) with a cell-seeding density of 5 × 10^5^ cells/ml. Cells were maintained at 37 °C in a humidified 5% CO_2_ incubator. 17.3 kDa ${\rm{TNF}}{\rm{\alpha }}$ (GenScript) was reconstituted in double-distilled water to a concentration of 100 μg/ml.

### Apoptosis detection by Annexin V/PI staining

U937 and Jurkat-T cells were stimulated with 100 ng/ml ${\rm{TNF}}{\rm{\alpha }}$ for the specified time under the standard incubating conditions. Cells were then harvested in the form of a pellet by centrifugation at 1000 rpm for 5 min at room temperature (RT). Harvested cells were resuspended in 1X Annexin binding buffer, and then stained with FITC-labeled Annexin V and PI dyes (BD Pharmingen, San Diego, CA, US). The fluorescence from the bound Annexin V and PI were detected using BD FACS Aria (BD Biosciences, San Jose, CA, US) within 30 min of dye addition.

### One-step state transition using ROA

Starting from a state $\bar{{\boldsymbol{v}}}$, using a unique permutation sequence *q* chosen uniformly randomly, the first node, say *i*, in *q* is updated by evaluating the corresponding Boolean function ${f}_{i}(\bar{{\boldsymbol{v}}})$ to arrive at an intermediate state ${\bar{{\boldsymbol{v}}}}^{i}$. The next node *j* in *q* is updated by evaluating ${f}_{j}({\bar{{\boldsymbol{v}}}}^{i})$ to obtain ${\bar{{\boldsymbol{v}}}}^{j}$. This procedure of finding the intermediate states is repeated until all nodes in *q* are exhausted. The final state thus achieved is the one that is a result of the one-step state transition originating from $\bar{{\boldsymbol{v}}}$ and corresponding to *q*.

### Partial logical steady-state analysis (pLSSA)

The network’s list of interactions with the associated logic were parsed into CellNetAnalyzer (CNA)^50^. Housekeeping and the (relevant) input nodes were set to ‘1’. “*Compute logical steady-state*” option in CNA was used to identify the nodes attaining partial Logical Steady-State (pLSS) and the corresponding Boolean value.

### Identification of fixed points

Starting from each of the ${2}^{N}$ states ${\mathbb{\in }}{\mathbb{R}}$, a one-step transition was computed using any one permutation, chosen uniformly randomly. The state for which the one-step transition using the chosen permutation results in the same state is the fixed point. Detailed procedure along with an illustration is provided in Supplementary Note 2.1.

### PageRank

PageRank vector ${\mathbb{P}}{{\mathbb{R}}}^{q}$ corresponding to ${M}^{q}$ after the ${q}^{{th}}$ permutation was estimated by solving ${\mathbb{P}}{{\mathbb{R}}}^{q}=\alpha {M}^{q}{\mathbb{P}}{{\mathbb{R}}}^{q}+\left(1-\alpha \right){e}^{T}$, where, $\alpha =0.85$ and ${e}^{T}$ is a one vector. ${\mathbb{P}}{{\mathbb{R}}}^{q}$ after every *q* was computed using the inner-outer iterative scheme^57^.

### Kendall’s-Tau rank correlation

The elements of ${\mathbb{P}}{{\mathbb{R}}}^{q}$ were re-sorted in the same order of placement of the states in ${\mathbb{P}}{{\mathbb{R}}}^{q-1}$, that is, PageRank order after the penultimate permutation. Kendall’s-Tau rank correlation ${\tau }^{q}{\mathbb{(}}{\mathbb{P}}{{\mathbb{R}}}^{q-1}{\mathbb{,}}{\mathbb{P}}{{\mathbb{R}}}^{q})$ was computed by comparing the rank of the states in ${\mathbb{P}}{{\mathbb{R}}}^{q}$ and ${\mathbb{P}}{{\mathbb{R}}}^{q-1}$, and subsequently enumerating the concordant and discordant pairs. A SciPy implementation *scipy.stats.kendalltau*, accessed from Matlab R2018b^®^, was used for this purpose^84^.

### Markov chain random walk approach for estimating Absorption probability

The state transition matrix ${M}^{{q}_{l}}$ was re-arranged into a canonical form $$\left[\begin{array}{cc}{\boldsymbol{I}} & {\boldsymbol{0}}\\ {\boldsymbol{R}} & {\boldsymbol{Q}}\end{array}\right]$$ where, ***I*** and ***0***, respectively are ($n\times n$) identity matrix denoting self-loops for ${\rm{FPs}}$ and ($n\times ({2}^{N}-n)$) zero matrix with *n* being the number of ${\rm{FPs}}$ in the STG. While matrix ***R*** specifies the transition probability of states transitioning directly into a FP, ***Q*** captures that into any other transient state. Absorption probability ${\mathcal{p}}_{{\rm{F}}{{\rm{P}}}_{i}}^{\bar{{\boldsymbol{v}}}}$ to reach an attractor ${\rm{F}}{{\rm{P}}}_{i}$ from any transient state $\bar{{\boldsymbol{v}}}$ due to Markov chain random walk on the STG is given by the ${i}^{{\rm{th}}}$ column of ${\left({\boldsymbol{I}}-{\boldsymbol{Q}}\right)}^{-1}{\boldsymbol{R}}$ ^85^. ${\left({\boldsymbol{I}}-{\boldsymbol{Q}}\right)}^{-1}{\boldsymbol{R}}$ was calculated using stabilized bi-conjugate gradient (*bicgstab*) iterative scheme in Matlab R2018b^®^. The *bicgstab* converged usually within 5 to 7 iterations.

### Time varying conditional probability of finding a node being active

The conditional probability of a node, such as ${\rm{NF}}{\rm{\kappa }}{\rm{B}}$, being updated to or maintained in an active state in a cell with a certain start state ${\bar{{\boldsymbol{v}}}}_{0}$ having to ability to reach both apoptosis and pro-survival attractors is governed by the multiple signal flow paths from ${\bar{{\boldsymbol{v}}}}_{0}$. These signal flow paths may overlap. All signal flow paths from ${\bar{{\boldsymbol{v}}}}_{0}$ are first identified (Methods). Intermediate states in these signal flow paths are aligned according to the update steps. Distinct states appearing in every update step across these signal flow paths are captured in an associated ${t}_{\max }\times {2}^{N}$ connectivity matrix (${\mathbb{C}}$), where ${t}_{\max }$ is that maximum pseudo-time step by when all signal flow paths from ${\bar{{\boldsymbol{v}}}}_{0}$ have reached a fixed point or a set of states that recur thereafter. Note that the states in this recurring set are either (i) in the strongly connected component (SCC) or (ii) in any of the paths between the states in SCC and a FP or (iii) FPs themselves. The elements of the first and subsequent (${t}^{{th}}$ timestep) rows of ${\mathbb{C}}$ are respectively captured by

8 $${{\mathbb{C}}}_{1j}=\left\{\begin{array}{l}1,{\rm{if}}\,{M}_{{\bar{{\boldsymbol{v}}}}_{0}j}\,\ne \,0\\ 0,{\rm{otherwise}}\end{array}\,\right.$$

and

9 $${{\mathbb{C}}}_{{tj}}=\left\{\begin{array}{l}1,{\rm{if}}\mathop{\sum}\limits_{i}{M}_{{ij}}{{\mathbb{C}}}_{(t-1)i}\,\ne \,0\,{\rm{in}}\; {\rm{the}}\,{t}^{{\rm{th}}}{\rm{update}}\; {\rm{step}}\\ 0,{\rm{otherwise}}\end{array}\right.$$

For the TNFR1 network (Fig. 2), ${t}_{\max }=12$. (Procedure for finding ${t}_{\max }$ is in Supplementary Note 4.8). In the ${t}^{{\rm{th}}}$ update step, the overall transition probability of different one-step transitions resulting in a state *j* (with ${\rm{NF}}{\rm{\kappa }}{\rm{B}}$ updated to or maintained in an active state) in these signal flow paths are captured in a ${t}_{\max }\times {2}^{N}$ signal-flow-path matrix ${\mathbb{P}}$. The elements in the first and subsequent rows of ${\mathbb{P}}$ are respectively specified as

10 $${{\mathbb{P}}}_{1j}={M}_{{\bar{{\boldsymbol{v}}}}_{0}j}{\rm{|}}({v}_{{\rm{NF}\rm{\kappa}\rm{B}}}=1\ {in}\,j)\ \forall j={1,2}^{N}$$

and

11 $${{\mathbb{P}}}_{{tj}}=\mathop{\sum }\limits_{i=1}^{{2}^{N}}{M}_{{ij}}\,{{\mathbb{C}}}_{(t-1)i}{\rm{|}}({v}_{{\rm{NF}\rm{\kappa}\rm{B}}}=1\ {in}\,j)\ \forall j=1,{2}^{N}$$

For a start state ${\bar{{\boldsymbol{v}}}}_{0}$, the timestep dependent conditional probability ${\left.{{\mathbb{E}}}_{{\rm{NF}}{\rm{\kappa }}{\rm{B}}}\left(t\right)\right|}_{{\bar{{\boldsymbol{v}}}}_{0}}$ that ${\rm{NF}}{\rm{\kappa }}$B is active at the ${t}^{{\rm{th}}}$ timestep is the overall fraction of one-step transitions at step *t* leading to ${\rm{NF}}{\rm{\kappa }}$B either transitioning to active form or being maintained at “1”. This conditional probability for the first and ${t}^{{\rm{th}}}$ timestep, respectively, are given by

12 $${\left.{{\mathbb{E}}}_{{\rm{NF}}{\rm{\kappa }}{\rm{B}}}\left(1\right)\right|}_{{\bar{{\boldsymbol{v}}}}_{0}}=\mathop{\sum }\limits_{j=1}^{{2}^{N}}{{\mathbb{P}}}_{1j}$$

and

13 $${\left.{{\mathbb{E}}}_{{\rm{NF}}{\rm{\kappa }}{\rm{B}}}\left(t\right)\right|}_{{\bar{{\boldsymbol{v}}}}_{0}}=\frac{\mathop{\sum }\nolimits_{j=1}^{{2}^{N}}{{\mathbb{P}}}_{{tj}}}{\mathop{\sum }\nolimits_{j=1}^{{2}^{N}}{{\mathbb{C}}}_{(t-1)j}}$$

${\left.{{\mathbb{E}}}_{{\rm{NF}}{\rm{\kappa }}{\rm{B}}}\left(1\right)\right|}_{{\bar{{\boldsymbol{v}}}}_{0}}$ (Eqs. 12 and 13) specifies the ${\rm{NF}}{\rm{\kappa }}{\rm{B}}$ transients for a given start state in the BD modeling framework. Such transients estimated for all possible start states in the STG collectively gives the ensemble-level dynamics similar to that obtained from experimental measurements. This procedure was used to estimate the ensemble-level dynamics of PI3K and ${\rm{c}}{3}^{* }-{\rm{p}}17$ nodes in the TNFR1 network (Fig. 2).

### Inhibition of TAK1

Takinib (EDHS-206)^69^ (MedChemExpress) was dissolved in DMSO to a concentration of 10 mM and the stock was stored at −20°C until use. Cells were pre-treated with 20μM Takinib, which inhibits the activity of TAK1, for 1 hr before stimulating with 100 ng/ml ${\rm{TNF}}{\rm{\alpha }}$. As a control case for this, cells were treated only with Takinib for a prolonged duration, details of which are in Supplementary Note 4.7.

### Reporting summary

Further information on research design is available in the Nature Research Reporting Summary linked to this article.

### Supplementary information

Supplementary Information

Reporting Summary

## Data Availability

All data used in this study are available within the manuscript and the associated Supplementary Information files.

## Code availability

The codes are written in Matlab and are available via GitHub at https://github.com/ganeshIITB/BMProSPR.git.
