# Author Correction: Modulating the dynamics of NFκB and PI3K enhances the ensemble-level TNFR1 signaling mediated apoptotic response

**DOI:** 10.1038/s41540-024-00338-4

**Published:** 2024-01-19

**Authors:** Shubhank Sherekar, Chaitra S. Todankar, Ganesh A. Viswanathan

**Affiliations:** Department of Chemical Engineering, Indian Institute of Technology Bombay Powai, Mumbai, 400076 India

**Keywords:** Dynamic networks, Stochastic modelling, Systems analysis, Numerical simulations

Correction to: *npj Systems Biology and Applications* 10.1038/s41540-023-00318-0, published online 16 November 2023

In this article, reference 31 should have read “Matveeva, A., Fichtner, M., McAllister, K., McCann, C., Sturrock, M., & Longley, D. B. et al. Heterogeneous responses to low level death receptor activation are explained by random molecular assembly of the Caspase-8 activation platform. *PLoS Comput. Biol*. **15**, e1007374 (2019)”.

The original article has been corrected.

