# Supplementary material for: Modulating the dynamics of NFκB and PI3K enhances the ensemble-level TNFR1 signaling mediated apoptotic response
Source: NPJ Syst Biol Appl. 2023 Nov 16;9:57. doi: 10.1038/s41540-023-00318-0 (PMC10654705; doi:10.1038/s41540-023-00318-0)
Supplement: Supplementary file 1 — Supplementary Information [file 41540_2023_318_MOESM1_ESM.pdf]

Modulating the dynamics of NFκB and PI3K enhances the ensemble-level TNFR1 signaling mediated apoptotic response

Supplementary Information

Shubhank Sherekar, Chaitra S Todankar and Ganesh A Viswanathan*
Department of Chemical Engineering, Indian Institute of Technology Bombay, Powai
Mumbai – 400076
*Corresponding author (Email: ganeshav@iitb.ac.in)

Table of contents

Supplementary Note 1: Effect of TNFα in cells, TNFR1 signaling network and its Boolean representation..4

Supplementary Note 2: Identification of fixed point attractors………………..………......11

Supplementary Note 3: Benchmarking of the BM-ProSPR algorithm……….…………… ..14

Supplementary Note 4: Boolean dynamics of TNFR1 signalling network…………..………25

List of figures

Supplementary Figure 1: TNFα mediated pro-survival and apoptosis response in U937 (Panel I) and Jurkat-T cells (Panel II)………………..…………. …………….. ……………..…..4

Supplementary Figure 2: Fixed point attractors……………………………………….……..12

Supplementary Figure 3: Effect of state space size on the number of function evaluations…13

Supplementary Figure 4: 6-node T-cell Large Granular Lymphocyte (T-LGL) apoptosis network……………………………………………………………………………………14

Supplementary Figure 5: Permutation guided evolution of the state transition graph (STG)……………………………………………………………………………………...15

Supplementary Figure 6: Dependence of the metrics of evolution of STG on permutations and comparison of absorption probabilities to reach FPs computed using partial STG with those specified by complete STG..….....……………………………………………………..........18

Supplementary Figure 7: 8-node developmental transcription factor permitting multiple phenotypes……………………………………………………………………………...20

Supplementary Figure 8: FP reachability……………………….…………………………22

Supplementary Figure 9: Multiple FP reachability………..…………………………………23

Supplementary Figure 10: Sensitivity analysis of absorption probability distribution………24

Supplementary Figure 11: Metrics and pro-survival absorption probabilities for the three input and perturbation conditions…..…………………………………………………..…………25

Supplementary Figure 12: An illustration of alignment of the signal flow paths in STG as per the pseudo-time steps………………………………………….……………………………29

Supplementary Figure 13: Dynamics of the conditional probability of node……….….……30

Supplementary Figure 14: Steady-state probabilities of TNFR1 network settling into different phenotypes…………………………………………………………………………......31

Supplementary Figure 15: An illustration of a few one-step state transitions in case of TNFα stimulated TNFR1 and TNFR1$^{\Delta}$ network……………………………………………………......32

Supplementary Figure 16: Four quadrant plots of apoptosis response in TAK1 inhibited U937 and Jurkat-T cells……………………………………………………………………..…....33

Supplementary Figure 17: TNFα mediated pro-survival and apoptosis response in TAK1 inhibited U937 and Jurkat-T cells.…………………...……………………………….. ……34

Supplementary Figure 18: The distribution of the start states requiring a certain maximum pseudo-time points (t) in the connectivity matrix $\mathbb{C}$………..………………….……………35

List of Tables

Supplementary Table 1: Entities in TNFR1 network (Fig. 2, main text) along with its classification and position in the Boolean state....….. …………………………..……......7

Supplementary Table 2: Boolean functions for entities. &, | and ~ operators respectively capture AND, OR and NOT operations..…..……………… ……………………..…………………8

Supplementary Table 3: partial logical steady-state (pLSS) computed for different input conditions in TNFR1 signaling network using CellNetAnalyzer. State of the node that turned out “undetermined” from pLSS analysis and therefore can either be 0 or 1 are marked “X”…… ….9

Supplementary Table 4: List of states, sorted in the increasing order of state ID, corresponding to the T-LGL network (Supplementary Figure 4) along with absorption probability $\wp_{\mathrm{FP}_1}^{\bar{v}}$. The probability of a state reaching FP_2 is $\wp_{\mathrm{FP}_2}^{\bar{v}} = 1 - \wp_{\mathrm{FP}_1}^{\bar{v}}$. Note that the order in which the states are presented in merely for convenience purposes and do not reflect any specific preference to a state or otherwise……………………………………………………………………..…………19

Supplementary Table 5: States corresponding to different phenotypic responses of 8 node developmental transcriptional network regulating the spinal cord ventrilization.……………......21

Supplementary Table 6: BM-ProSPR predicted minimum number of permutations q_l for reliable estimation of the partial state transition matrix for different input and perturbation conditions on the TNFR1 network.………..……………...…………………………….......26

Supplementary Table 7: FPs reached by activated TNFR1 signaling network after introduction of perturbations. FP1 and FP2, respectively refers to pro-survival and apoptotic phenotypes. Procedure described in Supplementary Note 2.1 was used finding the FPs……….................26

Supplementary Table 8: Boolean functions corresponding to the nodes in the random network constructed using Configuration model. &, | and ~ operators respectively capture AND, OR and NOT operations. ………………..………………………………………………………28

Supplementary Table 9: Boolean values and absorption probability to reach apoptosis FP ($\wp_{\mathrm{FP}_2}^{\bar{v}}$) for the states in the signal flow paths of TNFR1 and TNFR1$^{\Delta}$ network in Supplementary Figure 14………………………………………………………………………….. ...32

Supplementary Note 1: Effect of TNFα in cells, TNFR1 signaling network and its Boolean representation

Supplementary Note 1.1: Apoptotic response observed in U937 and Jurkat-T cells

Apoptosis levels in U937 and Jurkat-T cells under basal (no stimulation) and TNFα stimulation were measured using Annexin V-PI assay at ensemble-level (Methods, main text). We stimulated U937 and Jurkat-T cells with 100 ng/ml TNFα. While stimulation of U937 cells were for 16, 24 and 32 hours, Jurkat-T cells were exposed to the cytokine for 12 and 18 hrs. For every condition and time point, experiments were performed in triplicate. Pro-survival and apoptotic responses obtained in U937 cells after 24 hrs without or with TNFα stimulation are shown in Supplementar Figure 1A-B. While ~72% cells were present in survival state, rest exhibited apoptotic response.

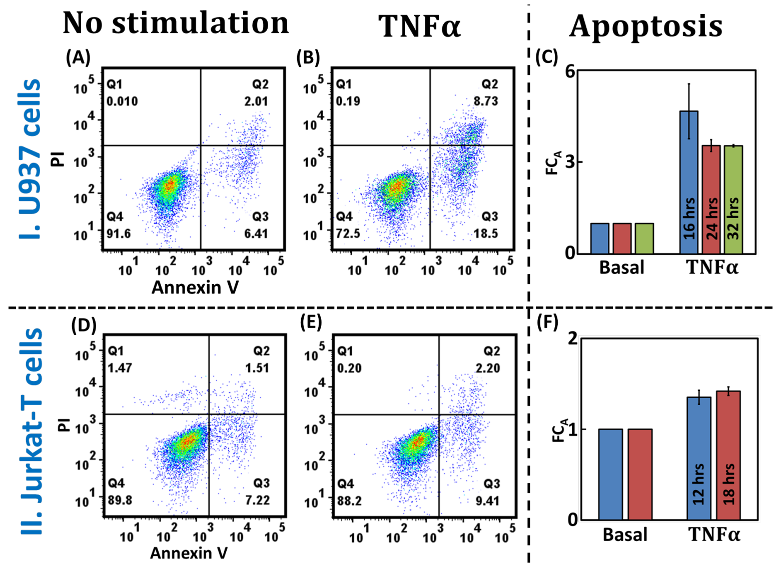

Supplementary Figure 1: TNFα mediated pro-survival and apoptosis response in U937 (Panel I) and Jurkat-T cells (Panel II). (A) and (B) shows the four-quadrant plot capturing different U937 cell states under no stimulation condition and 100 ng/ml TNFα stimulation for 24 hours. (D) and (E) shows the four-quadrant plot capturing different Jurkat-T cell states under no stimulation condition and 100 ng/ml TNFα stimulation for 18 hours. While cells in quadrant Q4 specifies the population expressing pro-survival response, combination of those in quadrants Q2 and Q3 together reflects apoptotic cells. (C) and (F) shows the effect of exposure time on the apoptosis fold change (FC_A) due to TNFα stimulation in U937 and Jurkat-T cells, respectively. FC_A is the ratio of the fraction of population undergoing apoptosis under stimulation and basal (no stimulation) conditions. Error bars represent mean ± standard deviation across three independent replicates.

Apoptosis fold change achieved in U937 cells remained unchanged after 16 hrs suggesting that the cells have a reached a steady-state (Supplementary Figure 1C). Similarly, Jurkat-T cells achieved a steady-state at ~12 hrs (Supplementary Figure 1F). Steady-state fold-change for U937 and Jurkat-T cells are ~3.5 and ~1.4, respectively (Supplementary Figures 1C,F).

Supplementary Note 1.2: TNFR1 signaling network

Signaling downstream Tumor Necrosis Factor Receptor 1 (TNFR1) is triggered by the binding of 17 kDa $\mathrm{TNF\alpha}$ to it.[1] Following activation, TNFR1 signals both cell death and cell survival via $\mathrm{Comp1}$, a complex consisting of TRADD, $\mathrm{TRAF2}$, $\mathrm{RIP1}$, $\mathrm{cIAP1/2^*}$.[2,3] In the cell-survival arm, the interaction between $\mathrm{IKK^*}$ and $\mathrm{Comp1}$ forms a intermediate complex $\mathrm{Comp1-IKK^*}$. $\mathrm{Comp1-IKK^*}$ essentially captures the complex made by $\mathrm{TAK1}$ which activates the $\mathrm{IKK^*}$ from $\mathrm{Comp1}$ bound to $\mathrm{RIPK1}$ via sequential phosphorylation.[4-7] $\mathrm{Comp1-IKK^*}$ prevents the activation of RIPK1 by phosphorylating at specific location to remain in $\mathrm{Comp1}$.[5] In resting cells, activated $\mathrm{I\kappa B}$ ($\mathrm{I\kappa B^*}$) is bound to $\mathrm{NF\kappa B}$ as $\mathrm{NF\kappa B-I\kappa B^*}$.[8] Deactivation of $\mathrm{I\kappa B^*}$ by $\mathrm{IKK^*}$ or $\mathrm{Comp1-IKK^*}$ leads to its disassociation from the $\mathrm{NF\kappa B-I\kappa B^*}$ complex and thereby releasing the $\mathrm{NF\kappa B}$ (functional form).[3] $\mathrm{NF\kappa B}$ transcribes $\mathrm{I\kappa B^*}$ to regulate its own expression level. $\mathrm{BCL-2}$ is overexpressed in leukemia cells such as acute myeloid leukemia (AML).[9,10] Since U937 is from the AML lineage, $\mathrm{BCL-2}$ will be overexpressed in the cells considered and such over-expression has been shown to degrade cytoplasmic $\mathrm{I\kappa B^*}$ under $\mathrm{TNF\alpha}$ stimulation.[11,12] This degradation induces $\mathrm{NF\kappa B}$.[13] This implies that $\mathrm{I\kappa B^*}$, when present, sequesters the available $\mathrm{NF\kappa B}$ and thereby, playing an inhibitory role on $\mathrm{NF\kappa B}$. $\mathrm{NF\kappa B}$ up-regulates $\mathrm{PI3K}$,[14,15] which subsequently activates $\mathrm{Raf1}$.[16] Both $\mathrm{PI3K}$ and $\mathrm{Raf1}$ promote the transition from the inactive to active forms of IKK,[17] and resulting in a feedback back loop involving these along with $\mathrm{I\kappa B^*}$ and $\mathrm{NF\kappa B}$. On the other hand, $\mathrm{NF\kappa B}$ mediates transcription of $\mathrm{A20}$ and $\mathrm{IKK^*}$ directly regulates it via phosphorylation.[18] $\mathrm{A20}$ regulates the activity of $\mathrm{NF\kappa B}$ by deubiquitination of $\mathrm{RIPK1}$ and thereby preventing $\mathrm{TAK1}$ activation.[19] Downstream, $\mathrm{NF\kappa B}$, being a master regulator, activates various apoptosis inhibitors ($\mathrm{cIAP1/2}$, XIAP, $\mathrm{BCL-xL}$ and $\mathrm{BCL-2}$) and thereby, plays a key role in apoptotic response.[8,20] $\mathrm{PI3K}$ activates PKB which eventually controls the intrinsic apoptotic response.[21]

In the apoptotic arm of the TNFR1 signalling, $\mathrm{Comp1}$ activates $\mathrm{Comp2}$ which is inhibited by $\mathrm{Comp1 - IKK^*}$. The direct activation of $\mathrm{Comp2}$ by TNFR1 captures the RIPK1 independent apoptosis. Comp2 then activates caspase 8 ($\mathrm{c8}$) via a series of interactions ($\mathrm{Comp2}$, FADD, $\mathrm{c8}$).[22,23] Activated caspase 8 ($\mathrm{c8^*}$) activates the subunit of caspase3 ($\mathrm{c3^* - p20}$).[24] While $\mathrm{c3^* - p20}$ or $\mathrm{c8^*}$ can activate $\mathrm{c3^* - p17}$, XIAP is known to inhibit both these interactions.[25,26] On the other hand, $\mathrm{c3^* - p20}$ is also activated via the intrinsic apoptotic pathway originating from Bax, the levels of which is controlled by an inhibitory action exhibited by $\mathrm{BCL - xL}$ and $\mathrm{PI3K}$.[27,28] Bax activates smac which is involved in a feedback loop consisting of $\mathrm{c3^* - p17}$ to activate $\mathrm{c3^* - p20}$ in the absence of XIAP.[29-33] $\mathrm{c3^* - p17}$ cleaves $\mathrm{BCL - xL}$ to allow signal flow through Bax and inhibit anti-apoptotic pathways.[34,35] $\mathrm{BCL - 2}$ is expected to be highly expressed in U937 cells being from the acute myeloid leukemia lineage[9] and its cleavage requires large amounts of $\mathrm{caspase - 3}$.[34] Therefore, we have not considered this interaction in the Boolean model. $\mathrm{c3^* - p17}$ then executes $\mathrm{Apoptosis}$ by inhibiting downstream protein Parp* that inhibits CAD* (which is responsible for DNA fragmentation and chromatin condensation).[36]

Fas receptor in the presence of FasL leads to apoptotic response by interacting with FADD and forms DISC complex. DISC activates $\mathrm{c8}$ directly through complex formation.[37] All other interactions downstream of $\mathrm{c8^*}$ is same as that in TNFR1 signaling.[38] Since most of apoptosis regulating molecular players are shared between Fas and TNFR1 signaling networks, Fas signalling may be considered a positive control for TNFα mediated apoptotic phenotypic response.

The Boolean function of a node is captured with the combination of three fundamental Boolean logics, AND (&), OR (|) and NOT (~) which depends on the nature of interactions regulating that node. AND logic is used when the higher levels of two or more nodes are strictly required for a particular node. For example, $\mathrm{Comp1}$ gets activated only when TNFR1 receptor along with all the necessary components are present adequately in the system. Hence, $f_{\mathrm{Comp1}} = \mathrm{TRADD\ \&\ TRAF2\ \&\ RIP\ \&\ TNFR1\ \&\ cIAP1/2^*}$. OR logic is implemented when multiple nodes can individually activate a downstream node and NOT logic denotes negative influence on the nodes. The logic assigned to capture the effect of multiple interactions affecting a node is decided based on pertinent literature.

Supplementary Note 1.3: Nodes in the network, Boolean functions and partial Logical steady state analysis

List of nodes in the TNFR1 network (Fig. 2, main text) along with the classification, i.e., housekeeping/input/signaling/output entity is in Supplementary Table 1. Besides, the adopted order of placement of the entities in the network state ($\bar{\boldsymbol{v}}$) is specified in the 'Alignment' column in Supplementary Table 1. The Boolean functions corresponding to the signaling and output entities are in Supplementary Table 2. Note that while the Boolean values of the housekeeping entities are fixed at a certain pre-decided value, those of input nodes are specified *a priori* depending on the stimulation condition considered.

Supplementary Table 1: Entities in TNFR1 network (Fig. 2, main text) along with its classification and position in the Boolean state.

Name of the entity	Short name	Alignment	Classification
TRADD	TRADD	1	Housekeeping[39]
RIPK1	RIP	2	Housekeeping[40]
TRAF2	TRAF2	3	Housekeeping[41]
FADD	FADD	4	Housekeeping[42]
$\mathrm{p}-14-3-3$	$\mathrm{p}-14-3-3$	5	Housekeeping[43]
[c8]	c8	6	Housekeeping[44]
[IKK]	IKK	7	Housekeeping[5]
[cIAP1/2]	cIAP1/2	8	Housekeeping[45]
[PARP]	PARP	9	Housekeeping[46]
TNFα	TNFα	10	Input
FasL	FasL	11	Input
TNFR1	TNFR1	12	Signaling
complex 1	Comp1	13	Signaling
complex 2	Comp2	14	Signaling
$\mathrm{c8}^{*}-\mathrm{complex\ 2}$	$\mathrm{c8}^{*}-\mathrm{Comp2}$	15	Signaling
Fas	Fas	16	Signaling
DISC	DISC	17	Signaling
$\mathrm{c8}^{*}-\mathrm{DISC}$	$\mathrm{c8}^{*}-\mathrm{DISC}$	18	Signaling
$\mathrm{c8}^{*}$	$\mathrm{c8}^{*}$	19	Signaling
cIAP1/2	$\mathrm{cIAP1/2}^{*}$	20	Signaling
$\mathrm{c3}^{*}-\mathrm{p20}$	$\mathrm{c3}^{*}-\mathrm{p20}$	21	Signaling
$\mathrm{c3}^{*}-\mathrm{p17}$	$\mathrm{c3}^{*}-\mathrm{p17}$	22	Signaling
PARP	PARP^{*}	23	Signaling
CAD	CAD	24	Signaling
PI3K	PI3K	25	Signaling
PKB	PKB	26	Signaling
Raf1	Raf1	27	Signaling

Bad − 14 − 3 − 3	Bad − 14 − 3 − 3	28	Signaling
BCL − xL	BCL − xL	29	Signaling
Bax	Bax	30	Signaling
Smac	smac	31	Signaling
IκBα	IκB*	32	Signaling
IKK	IKK*	33	Signaling
complex 1 − IKK	Comp1 − IKK*	34	Signaling
FLIP	FLIP	35	Signaling
XIAP	XIAP	36	Signaling
A20	A20	37	Signaling
BCL − 2	BCL − 2	38	Signaling
NFκB	NFκB	39	Output
Apoptosis	Apoptosis	40	Output

Supplementary Table 2: Boolean functions for entities. $\&, \vert$ and $\sim$ operators respectively capture AND, OR and NOT operations.

**Node Name**	**Boolean function name**	**Boolean function**
TNFR1	f_{TNFR1}	TNFα
Comp1	f_{Comp1}	TRADD & TRAF2 & RIP & TNFR1 & cIAP1/2*
Comp2	f_{Comp2}	((Comp1 & FADD) $\vert$ ~Comp1 − IKK*) $\vert$ (TNFR1 & FADD)
c8* − Comp2	$f_{\mathrm{c8^*-Comp2}}$	c8 & Comp2
Fas	f_{Fas}	FasL
DISC	f_{DISC}	Fas & FADD
c8* − DISC	$f_{c8^*\mathrm{-DISC}}$	c8 & DISC
c8*	f_{c8^*}	c8* − Comp2 $\vert$ (c8* − DISC & ~FLIP)
cIAP1/2*	$f_{\mathrm{cIAP1/2^*}}$	cIAP1/2 $\vert$ NFκB $\vert$ ~smac
c3* − p20	$f_{c3^*\mathrm{-p20}}$	(smac & c3* − p17 & ~XIAP) $\vert$ c8*
c3* − p17	$f_{c3^*\mathrm{-p17}}$	(c3* − p20 & ~XIAP) $\vert$ (c8* & ~XIAP)
PARP*	f_{PARP3^*}	PARP & ~c3* − p17
CAD	f_{CAD}	~PARP*
PI3K	f_{PI3K}	NFκB
PKB	f_{PKB}	PI3K
Raf1	f_{Raf1}	PI3K
Bad − 14 − 3 − 3	$f_{\mathrm{Bad-14-3-3}}$	P − 14 − 3 − 3 & PKB
BCL − xL	$f_{\mathrm{BCL-xL}}$	NFκB & ~c3* − p17
Bax	f_{Bax}	~BCL − xL & ~Bad − 14 − 3 − 3 & ~BCL − 2
smac	f_{smac}	Bax
IκB*	$f_{\mathrm{I\kappa B^*}}$	(NFκB &~BCL − 2)$\vert$(~BCL − 2 & ~IKK* & ~Comp1 − IKK*

IKK^*	$f_{\text{IK}K^*}$	IKK & PI3K & Raf1
$\text{Comp1} - \text{IKK}^*$	$f_{\text{Comp1}-\text{IK}K^*}$	Comp1 & IKK^* & $\sim\text{A20}$
FLIP	f_{FLIP}	NFκB
XIAP	f_{XIAP}	NFκB
A20	f_{A20}	NFκB & IKK^*
$\text{BCL} - 2$	$f_{\text{BCL}-2}$	NFκB
NFκB	$f_{\text{NF}\kappa\text{B}}$	$\sim\text{I}\kappa\text{B}^*$
Apoptosis	$f_{\text{Apoptosis}}$	CAD & $\sim\text{PARP}^*$

Supplementary Note 1.4: partial Logical steady-state analysis (pLSSA)

pLSSA was performed on the TNFR1 network for all cases (including no stimulation) considered using CellNetAnalyzer.[47] Nodes that attained a pLSS for different conditions considered in this study are in Supplementary Table 3. Nodes marked "X" in Supplementary Table 3 are those whose LSS remained undetermined for the considered stimulation condition. Nodes that attain pLSS will remain in that Boolean value irrespective of the dynamic variation in the values of all other entities. Note that the case of Takinib, the pLSSA fixed nodes are same as that of Basal, indicating that the overall response of TAK1 inhibited cells with no stimulation will display response same as that of Basal case.

Supplementary Table 3: partial logical steady-state (pLSS) computed for different input conditions in TNFR1 signaling network using CellNetAnalyzer.[47] State of the node that turned out "undetermined" from pLSS analysis and therefore can either be 0 or 1 are marked "X".

Node ID	Signaling/ Output nodes	pLSS				
		Basal	TNFα	FasL	TNFα + Takinib	Takinib
12	TNFR1	0	1	0	1	0
13	Comp1	0	1	0	1	0
14	Comp2	0	1	0	1	0
15	$\text{c8}^* - \text{Comp2}$	0	1	0	1	0
16	Fas	0	0	1	0	0
17	DISC	0	0	1	0	0
18	$\text{c8}^* - \text{DISC}$	0	0	1	0	0
19	c8^*	0	1	X	1	0
20	cIAP1/2^*	1	1	1	1	1
21	$\text{c3}^* - \text{p20}$	X	1	X	1	X
22	$\text{c3}^* - \text{p17}$	X	X	X	X	X

23	$PARP^*$	X	X	X	X	X
24	CAD	X	X	X	X	X
25	PI3K	X	X	X	X	X
26	PKB	X	X	X	X	X
27	Raf1	X	X	X	X	X
28	Bad − 14 − 3 − 3	X	X	X	X	X
29	BCL − xL	X	X	X	X	X
30	Bax	X	X	X	X	X
31	smac	X	X	X	X	X
32	$I\kappa B^*$	X	X	X	X	X
33	IKK^*	X	X	X	X	X
34	$Comp1 - IKK^*$	0	X	0	0	0
35	FLIP	X	X	X	X	X
36	XIAP	X	X	X	X	X
37	A20	X	X	X	X	X
38	BCL − 2	X	X	X	X	X
39	NFκB	X	X	X	X	X
40	Apoptosis	X	X	X	X	X

Supplementary Note 2: Identification of fixed point attractors

Supplementary Note 2.1: Procedure for identification of fixed points

Asynchronous updating enables capturing behavior of an ensemble of cells, wherein individual cells initially at a certain state can choose its fate resulting in a cell-specific phenotype.[48] Such an absorbing state can be either a fixed point consisting of a single state or a cyclic attractor, which meanders over a set of states. We consider only fixed points in this study since the TNFR1 network (Fig 2, main text) permits only fixed-point attractors (FPs). In order to identify the FP in STG, we chose a permutation uniformly randomly and performed a one-step state transition using ROA (Methods, Main text) from all states in state-space ($\mathbb{R}$). For this purpose, we chose the case of TNFα stimulation. (Without loss of generality, the approach described here is applicable to any stimulation/condition of the network.) In Supplementary Figure 2, using the permutation sequence 25,33,39,40,27,24,32,29,30,22,28,35,23,26,34,36,31,38,37 we show a few one-step transitions. Starting from a state, one-step transition using any permutation can lead to three outcomes, *viz.*,

(i) reach a new state which is not a fixed point and therefore is a transient one. For example, the one-step state transition $\bar{\boldsymbol{v}}_1 \rightarrow \bar{\boldsymbol{v}}_2$ in Supplementary Figure 2.

(ii) reach a new state which is a fixed point. For example, the one-step state transition $\bar{\boldsymbol{v}}_2 \rightarrow \bar{\boldsymbol{v}}_3$ in Supplementary Figure 2.

(iii) reach the same as the start state. For example, the one-step state transitions $\bar{\boldsymbol{v}}_3 \rightarrow \bar{\boldsymbol{v}}_3$, $\bar{\boldsymbol{v}}_4 \rightarrow \bar{\boldsymbol{v}}_4$ in Supplementary Figure 2.

One-step transition from a state can lead to itself if and only if the state is a FP attractor (case (iii) above). Thus, $\bar{\boldsymbol{v}}_3$ is a FP attractor. Since the 'Apoptosis' output node takes a Boolean value of 1 with the other output one being 0, this FP is deemed apoptosis attractor. On the other hand, $\bar{v}_4$ is a pro-survival FP as the Boolean value of 'NFκB' output node only takes the Boolean value of 1. We used this approach to identify the FPs for the case of unstimulated and FAS stimulated network (Fig. 2, main text) as well. These are presented in Supplementary Figure 2 below.

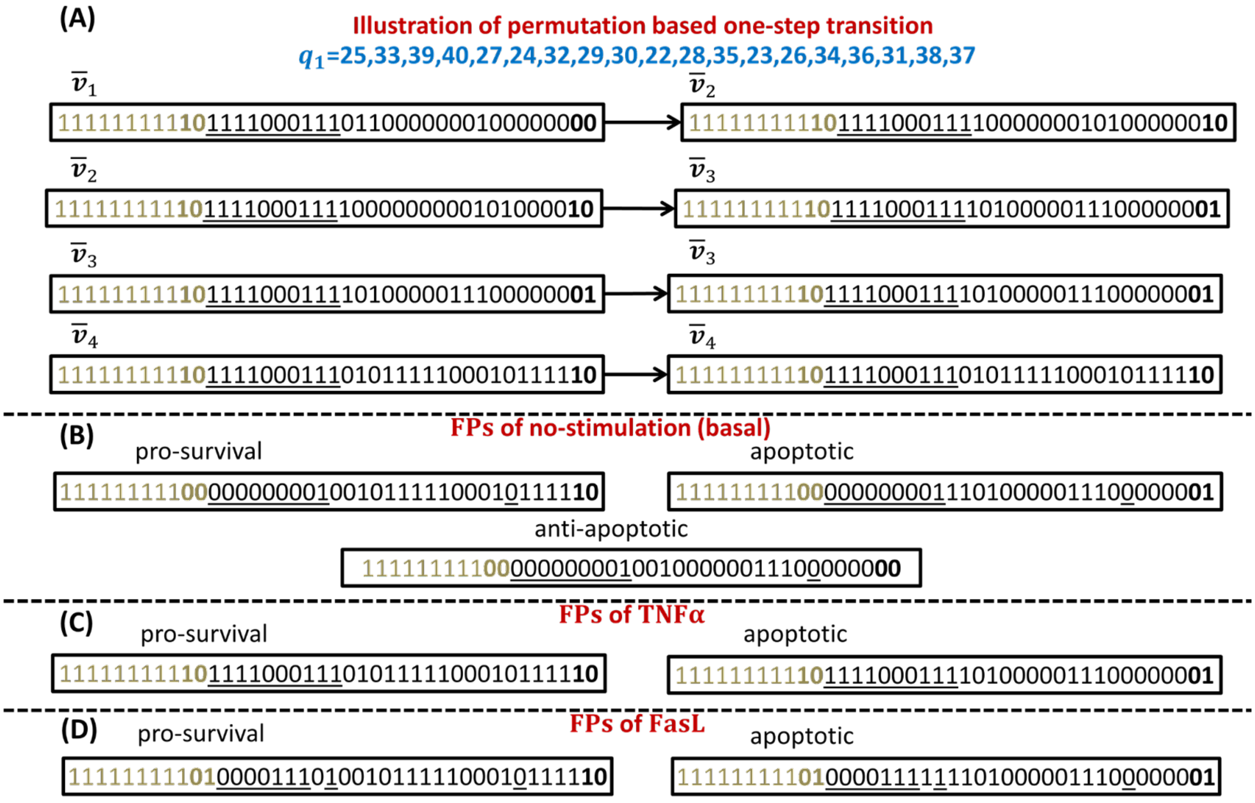

Supplementary Figure 2: Fixed point attractors (A) One-step state transition between transient states, transient states to FP attractor, FP attractor to FP attractor from a randomly chosen permutations which is mentioned in blue color. The fixed points of the TNFR1 signaling network (Fig. 2, main text) under no stimulation, TNFα stimulation and FasL stimulation are in (B), (C) and (D), respectively.

Supplementary Note 2.2: Permutations for one-step transitions in Figure 3, main text

The permutations, chosen uniformly randomly, used for simulating the one-step transitions reported in Fig. 3 are

i) ST1- 40, 22, 26, 29, 39, 30, 37, 36, 32, 31, 33, 37, 25, 35, 24, 28, 23, 27, 34
ii) ST2- 36, 22, 23, 40, 26, 32, 33, 35, 39, 29, 28, 38, 30, 24, 25, 31, 37, 27, 34
iii) ST3- 22, 34, 30, 25, 33, 36, 29, 32, 31, 39, 40, 28, 27, 38, 37, 35, 24, 26, 23
iv) ST4- 27, 32, 30, 38, 26, 36, 28, 37, 31, 33, 40, 23, 35, 39, 25, 22, 29, 34, 24

Supplementary Note 2.3: Boolean function evaluations for different size of network

Construction of a complete state transition graph and thereby, quantification of the corresponding state transition matrix (M) for any network having N nodes requires $2^N \times N! \times N$

Boolean function evaluations. The number of Boolean function evaluations increases exponentially with the state space size specified by 2^N, as shown in Supplementary Figure 3.

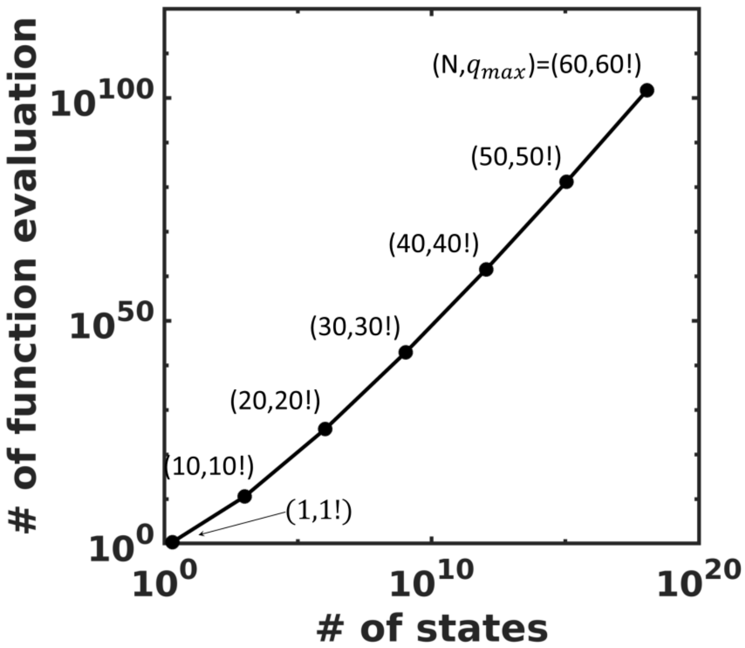

Supplementary Figure 3: Effect of the state space size, specified by 2^N, on the required number of function evaluations for the construction of the state transition graph. For a sample point considered, the number of nodes N in the network and the number of permutations q_{max} is specified respectively by the first and second elements within the brackets.

Supplementary Note 3: Benchmarking of the BM-ProSPR algorithm

<u>Supplementary Note 3.1</u>: <u>Evolution of STG</u>

As a motivating example, we consider a small 6 node network[48] to benchmark the BM-ProSPR algorithm (Supplementary Figure 4A). The six Boolean functions (f_i) are in Supplementary Figure 4B. Starting with a null STG (S^0) consisting of $2^6 = 64$ isolated states and assuming an initial seed number of permutations $q = q_0 \geq 1$, we construct a partial STG S^{q_0} corresponding to 0 (null), 1, and 2 permutations. Snapshots of partial STGs of the 6-node network are in Supplementary Figure 5A.

(A)

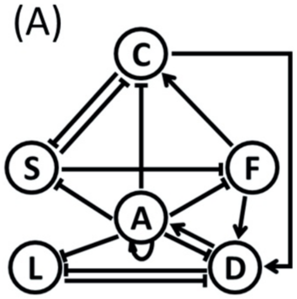

(B)

$$f_S(\overline{\boldsymbol{v}}) = \sim v_C \, \& \sim v_A$$
$$f_L(\overline{\boldsymbol{v}}) = \sim v_D \, \& \sim v_A$$
$$f_F(\overline{\boldsymbol{v}}) = \sim v_S \, \& \sim v_A$$
$$f_C(\overline{\boldsymbol{v}}) = v_F \, \& \sim(v_S \, | v_A)$$
$$f_D(\overline{\boldsymbol{v}}) = (v_C \, | v_F \, \& \sim v_L) \, \& \sim v_A$$
$$f_A(\overline{\boldsymbol{v}}) = v_D \, | \, v_A$$

Supplementary Figure 4: (A) 6-node T-cell Large Granular Lymphocyte (T-LGL) apoptosis network. Nodes A, C, D, F, L, and S, respectively represent Apoptosis, Ceramide, DISC, Fas, FLIP, and S1P. Arrows and hammers, respectively capture activation and inhibition interactions. (B) Boolean functions corresponding to the nodes in (A). &, |, and ~, respectively correspond to $AND, OR,$ and NOT logics.

While S^0 (Supplementary Figure 5A-i) consists of only isolated states marked with randomly chosen IDs, S^1 contains 64 one-step transitions (Supplementary Figure 5A-ii). These 64 directed links are either self-links from two FPs (state IDs 2 and 49, green circles), or a transition into one of the FPs (for e.g., 4 to 2, 33 to 49) or a transition between non-FP transient states (for e.g., 3 to 33). Supplementary Figure 5A-ii also shows signal flow paths (for e.g., 5 → 12 → 2, and 3 → 33 → 49) emanating from a state, traversing through other states and culminating in either of the attractors. Upon introduction of a second permutation, 64 new one-step transitions between as many pairs of states are added to S^1 leading to S^2 (Supplementary Figure 5A-iii). Out of these 64 pairs in S^2, 24 of them (blue arrows) did not have a link in S^1, while 40 of them (red arrows) accounted for the second one-step transitions between them. The other 24 links (black arrow) in S^2 correspond to those pairs between which a transition was *not* caused by the second permutation and therefore, are carried forward from S^1.

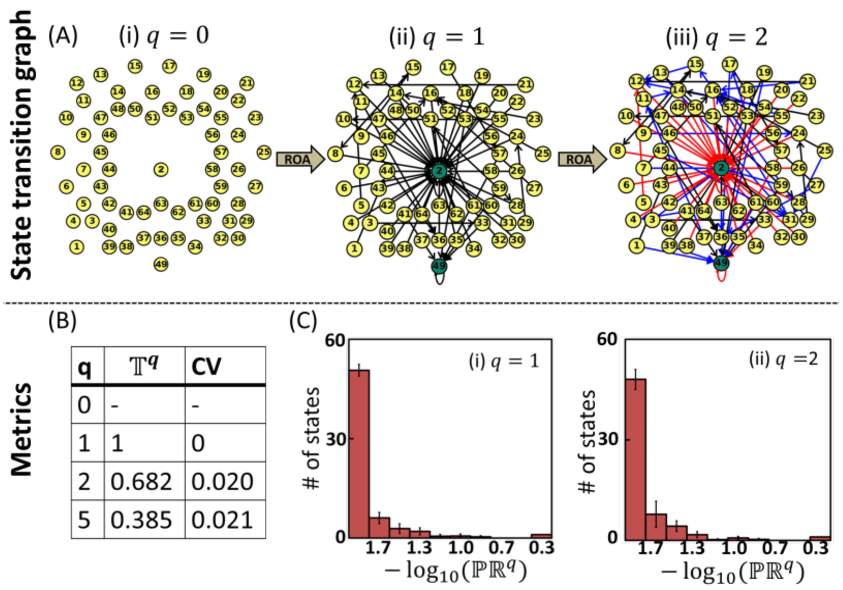

Supplementary Figure 5: Permutation guided evolution of the state transition graph (STG) underlying the Boolean model of 6-node T-LGL apoptosis network. (A) STG for 0 (null), 1 and 2 permutations. Note for every permutation, 64 new one-step transitions are added. For every one-step transition, BD simulations were performed using a permutation chosen uniformly randomly from $6!\,(= 720)$ sequences. Green and yellow circles capture the fixed points and other states, respectively. Black arrows in STG for $q = 2$ are those one-step transitions between a pair of states carried forward, as is, from that constructed for the previous permutation ($q = 1$). While blue arrows represent one-step state transition obtained between a pair of states due to permutation $q = 2$ but not found in the STG for $q = 1$, red arrows capture two one-step transitions, one each obtained between same pair of states for $q = 1$ and $q = 2$. (B) Temporality measure ($\mathbb{T}^q$) of the STG constructed for a few permutations. The values reported for $\mathbb{T}^q$ are averaged quantities over 50 random constructions of the STG for every q. The CV reflects the coefficient of variation of $\mathbb{T}^q$ over these random constructions. (C) Histogram of PageRank ($\mathbb{PR}$) of the states in STGs for $q = 1$ and 2. Note that the mean frequency and the corresponding standard deviation (captured by error bars) for every bin were estimated using 50 STG reconstructions.

Temporality measure ($\mathbb{T}^q$) corresponding to the partial STGs shows a decreasing trend with increasing permutations indicating convergence (Supplementary Figure 5B). PageRank ($\mathbb{PR}$) of all states in the null STG is 0. A comparison of the histogram of $\mathbb{PR}$ of all $\bar{\boldsymbol{v}}$ in S^1 and S^2 suggests that addition of 64 new one-step transitions causes a change in the topology (Supplementary Figure 5C-i, C-ii) suggesting the evolution of STG due to additional links and weights carried by those links between states in STG.

Supplementary Note 3.2: Effect of permutation on PageRank order

Kendall's Tau rank correlation[49] was used as a measure to compare PageRank order of states in STG with subsequent permutations. For this purpose, based on a comparison of PageRank of states in STG at q and $(q-1)^{th}$ permutation, the concordant and discordant pairs are identified. Total number of pairs in STG is $\binom{2^N}{2}$. Therefore, the sum of concordant (C) and discordant (D) pairs at a certain permutation for which the Kendall's Tau rank correlation is estimated, is given by

$$C^q + D^q = \binom{2^N}{2} = \frac{2^N(2^N-1)}{2} = 2^{N-1}(2^N-1), \quad (1)$$

where, C^q and D^q, respectively are the concordant and discordant pairs at the qth permutation estimated as described in section 2.3, main text.

Supplementary Note 3.3: Reliable computation of the steady-state probability using BM-ProSPR

We implemented the entire BM-ProSPR (Fig. 4) on the 6-node T-LGL network by assuming $q_0 = 1$ (seed permutations) and identified q_l needed to reliably estimate $P_{ss}^{\text{FP}_1}$ and $P_{ss}^{\text{FP}_2}$. We tested and benchmarked BM-ProSPR by implementing the algorithm for all 720 $(= q_{max})$ permutations, rather than terminating at the minimum required permutations q_l. Since the algorithm employs permutations chosen uniformly randomly, we implemented the BM-ProSPR on the T-LGL network 50 times, that is, 50 STG reconstructions, and estimated $\mathbb{T}$, $\mathbb{PR}$, $\mathbb{D}$ and q_l for each of these. Dependence of the $\mathbb{T}$, $\mathbb{PR}$ and $\mathbb{D}$ (averaged over 50 STG reconstructions) on the permutations are in Supplementary Figures 6A, 6B, and 6C, respectively. We contrast the variation of $\mathbb{T}^q$ (Supplementary Figure 6A, blue) with the distance measure[50] (Supplementary Figure 6A, red) given by

$$d_f(M^q, M^{q_{max}}) = \frac{\|M^q - M^{q_{max}}\|_2}{\|M^{q_{max}}\|_2} \quad (2)$$

where, $\|.\|_2$ is the Fröbenius norm. $d_f(M^q, M^{q_{max}})$ quantitates the extent to which M^q approximates $M^{q_{max}}$, decreases with increasing q and is zero for $q = q_{max}$ (Supplementary Figure 6A, red). This comparison clearly shows that $\mathbb{T}$ is indeed a better indicator of the evolution of the partial STG and accurately reflects the extent to which S^q may have evolved to

mimic the complete STG $S^{q_{max}}$ such that $M^q \approx M^{q_{max}}$. Moreover, temporality measure offers a unique advantage over Fröbenius norm-based distance as $\mathbb{T}$ is *amenable for large networks* for which complete STG and thereby $M^{q_{max}}$ are unavailable.

A comparison of the histograms for different permutations suggests that the $\mathbb{PR}$ distribution saturates after $q = 200$ indicating that $M^{q \geq 200} \approx M^{q_{max}}$ (Supplementary Figure 6B). This is substantiated by the sharp decrease in $\mathbb{D}$ with increasing q and insignificant variation for $q \geq 200$ (Supplementary Figure 6C). The conditions that satisfy the convergence test (Eqs (5) and (6), main text) require 286 ± 65 minimum number of permutations. Note that the relatively large coefficient of variance (CV) across instances of $\sim 23\%$ is due to the tiny state-space of the underlying STG. (We show that for large networks, such as that of TNFR1 network analyzed in the main text, the CV is indeed very small (see Supplementary Table 6 in Supplementary Note 4 below).) Assuming that $S^{286} \approx S^{q_{max}}$, we estimated the steady-state probability $P_{ss}^{\mathrm{FP}_i}$ (Eq. 2; main text) to reach FP_1 and FP_2 as 0.82 and 0.18, respectively.

For an accurate prediction $P_{ss}^{\mathrm{FP_i}}$, $\mathrm{i} = 1{,}2$, absorption probabilities to reach $\mathrm{FP_i}$ computed from M^{286} must converge to those estimated using $\mathrm{M}^{\mathrm{q_{max}}}$. A state can reach either one or both the fixed points FP_1 and FP_2. The probability of reaching an attractor from a certain state is its absorption probability. In Supplementary Table 4, we show the absorption probability $\wp_{\mathrm{FP}_1}^{\bar{v}}$ of all states reaching FP_1. Those having an absorption probability of 1 belong exclusively to the basin of attraction of that attractor. For example, states 000001 (state ID 2, Supplementary Table 4), 001011 (state ID 12, Supplementary Table 4) belong exclusively to the basin of attraction of FP_1. On the other hand, states 011111, 110000 and 110110 (state IDs 32, 49, and 55, respectively in Supplementary Table 4) belong exclusively to the basin of attraction of FP_2. All other states belong to both basin of attraction of FP_1 and FP_2 with $0 < \wp_{\mathrm{FP}_1}^{\bar{v}} = 1 - \wp_{\mathrm{FP}_1}^{\bar{v}} < 1$.

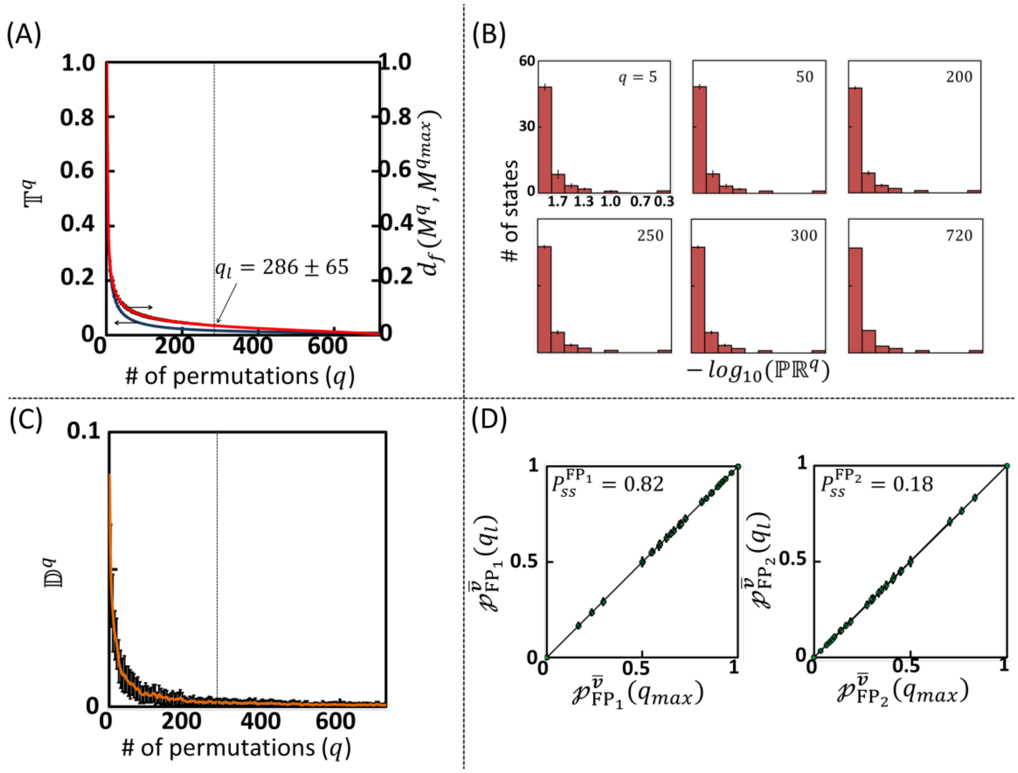

Supplementary Figure 6: (A) Comparison of the dependence of the Temporality measure $\mathbb{T}^q$ (blue) and the Fröbenius distance $d(M^q, M^{q_{max}})$ (red) on the number of permutations q. M^q and $M^{q_{max}}$ are the state transition matrices corresponding to the partial STG at q^{th} permutation and the complete STG at $q_{max} = 720$. q_l is the number of permutations required to construct reliable partial STG. (B) Dependence of the distribution of PageRank ($\mathbb{PR}$) of states on permutations. (C) Dependence of the discordant PageRank fraction across successive STG constructions with increasing number of permutations. (D) A comparison of the absorption probabilities computed using the partial STG at q_l with those specified by complete STG at $q_{max} = 720$ to reach the two attractors (FP_1 and FP_2) from 64 states. The fractions specified along with the comparison specify the steady-state probabilities of the T-LGL apoptosis network to reach the two attractors. Deviation from the ensemble average of the measures and absorption probabilities obtained over 50 STG reconstructions are presented as error bars.

A state $\bar{\boldsymbol{v}}$ having $p_{\mathrm{FP}}^{\bar{v}} = 1$ belongs exclusively to the basin of attraction of FP ($\mathbb{B}^{\mathrm{FP}}$). Out of 64 states in $\mathbb{R}$, 36 and 3 of them respectively belong exclusively to the basins of attraction of FP_1 (000001) and FP_2 (110000) (Supplementary Table 4). The remaining 25 states can reach either of the FPs, each with a finite absorption probability. Absorption probabilities computed from M^q estimated by BM-ProSPR and $M^{q_{max}}$ for both FPs are contrasted in Supplementary Figure 6D. This clearly proves that BM-ProSPR indeed predicts the absorption probabilities and

therefore, the steady-state probabilities of the 6-node network's ability to settle into the two fixed points.

Supplementary Table 4: List of states, sorted in the increasing order of state ID, corresponding to the T-LGL network (Supplementary Figure 4) along with absorption probability $\wp_{\mathrm{FP}_1}^{\bar{v}}$. The probability of a state reaching FP_2 $\wp_{\mathrm{FP}_2}^{\bar{v}} = 1 - \wp_{\mathrm{FP}_1}^{\bar{v}}$. Note that the order in which the states are presented in merely for convenience purposes and do not reflect any specific preference to a state or otherwise.

ID	State	$\wp_{\mathrm{FP}_1}^{\bar{v}}$	ID	State	$\wp_{\mathrm{FP}_1}^{\bar{v}}$	ID	State	$\wp_{\mathrm{FP}_1}^{\bar{v}}$
1	000000	0.235	23	010110	1	45	101100	1
2	000001	1	24	010111	0.907	46	101101	0.649
3	000010	1	25	011000	1	47	101110	1
4	000011	0.665	26	011001	0.501	48	101111	1
5	000100	1	27	011010	1	49	110000	0
6	000101	0.725	28	011011	1	50	110001	1
7	000110	1	29	011100	1	51	110010	0.934
8	000111	0.919	30	011101	1	52	110011	1
9	001000	1	31	011110	1	53	110100	0.499
10	001001	0.694	32	011111	0	54	110101	1
11	001010	1	33	100000	1	55	110110	0
12	001011	1	34	100001	0.811	56	110111	1
13	001100	1	35	100010	1	57	111000	0.548
14	001101	1	36	100011	0.500	58	111001	1
15	001110	1	37	100100	1	59	111010	0.859
16	001111	0.966	38	100101	0.553	60	111011	1
17	010000	1	39	100110	1	61	111100	0.587
18	010001	0.166	40	100111	0.863	62	111101	1
19	010010	1	41	101000	1	63	111110	0.592
20	010011	0.626	42	101001	0.296	64	111111	0.894
21	010100	1	43	101010	1			
22	010101	0.703	44	101011	0.835			

Supplementary Note 3.4: BM-ProSPR accurately predicts steady-state probability for multiple phenotypes

We demonstrate BM-ProSPR's ability to compute absorption probabilities to reach multiple phenotypes (FPs) by implementing the algorithm on an 8-node developmental transcription factor network regulating the spinal cord ventrilization in HEK293T cells (Supplementary Figure 7A).[51] The ventrilization network permits five progenitor cell types depending upon the activated

or inactivated state of different nodes. We frame Boolean functions for every node by capturing the inhibitory actions on

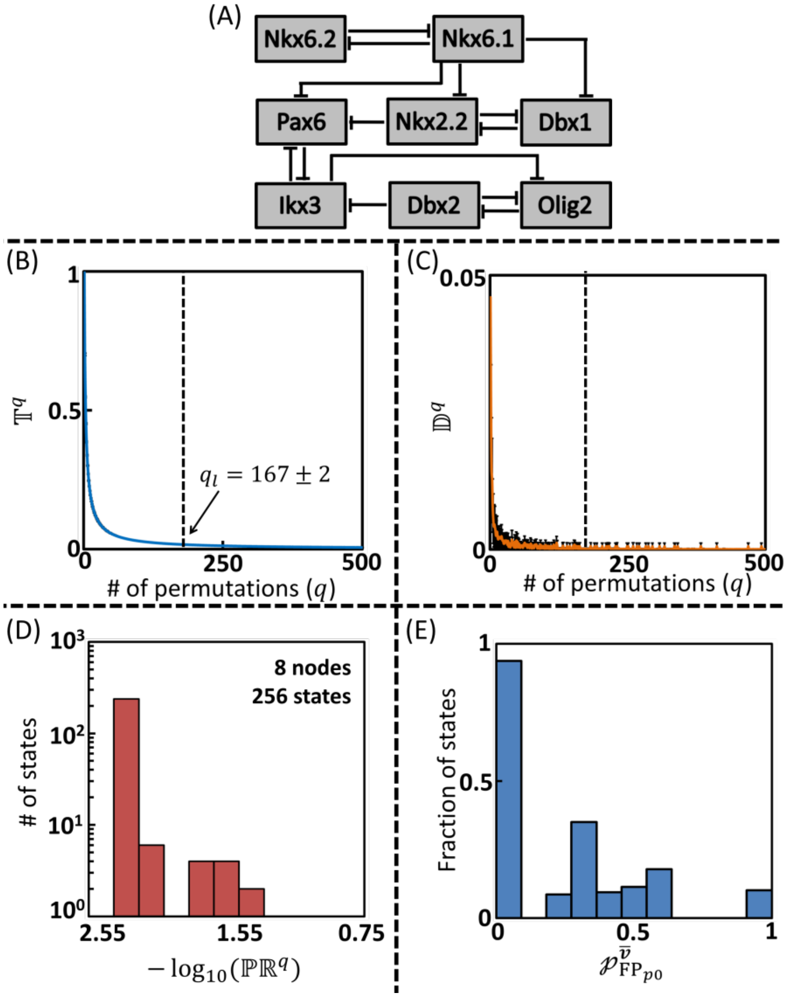

Supplementary Figure 7: 8-node developmental transcription factor network permitting multiple phenotypes. (A) 8-node network having nodes as transcription factors with inhibitory interactions between them. Dependence of (B) Temporality measure $\mathbb{T}$, and (C) Fraction of discordant pairs $\mathbb{D}$ on the permutations. (D) PageRank distribution for $q_l = 167$ permutation. (E) Distribution of the absorption probability for reaching $p0$ phenotype. Error bars in (B) and (C) capture the deviation from the corresponding ensemble average.

it by other entities in the network. For example, the Boolean function corresponding to Pax6 , inhibited by Nkx6.1, Nkx2.2, Ikx3, is

$$f_{\text{Pax6}}(\bar{\boldsymbol{v}}) = \sim(v_{\text{Nkx6.1}} | v_{\text{Nkx2.2}} | v_{\text{Ikx3}}) \quad (3)$$

where ~ and | represent NOT and OR logic, respectively. The fixed points (FPs) $p3, pMN, p2, p1$ and $p0$ of the network correspond to the five cell types and the respective states are in Supplementary Table 5.

Supplementary Table 5: States corresponding to different phenotypic responses of 8 node developmental transcriptional network regulating the spinal cord ventrilization.

Dorsal ⟶ Ventral

TFs↓ Cells→	$p3$	pMN	$p2$	$p1$	$p0$
Nkx6.2	0	0	0	1	0
Nkx6.1	1	1	1	0	0
Pax6	0	1	1	1	1
Nkx2.2	1	0	0	0	0
Dbx1	0	0	0	0	1
Ikx3	0	0	1	1	1
Dbx2	0	0	0	1	1
Olig2	0	1	0	0	0

State transition graph of the network consists of $2^8 = 256$ states and 50 randomized STG constructions were considered. Temporality measure and fraction of pairs having discordant PageRank across successive permutations show a decreasing trend with increase in permutations (Supplementary Figure 7B,C) indicating that the STG evolves rapidly. PageRank distribution is shown in Supplementary Figure 7D.

Only $q_l = 167 \pm 2$ permutations out of $q_{max} = 40320$ were required for reliable estimation of the partial state transition matrix M^{q_l}. While the complete STG will contain $40320 \times 256 = 1{,}03{,}21{,}920$ one-step state transitions, on an average, only a tiny fraction of $0.41\% = 167 \times 256 = 42752$ is needed for finding $\mathbb{S}^{q_l}$ required to estimate M^{q_l} reliably. A histogram of the absorption probability of $p0$ phenotype (Supplementary Figure 7E) clearly shows that a fraction of states belongs exclusively to the basin of attraction $p0$ and also several having $0 < \wp_{p0}^{\bar{v}} < 1$ indicating reachability to multiple phenotypes. In Supplementary Figure 8A, as an example, we show all signal paths originating from the state 01100011 (state ID 117) and terminating only in the FP pMN (state ID 113). While the probability of reaching pMN via signal path $117 \to 51 \to 115 \to 113$ is $0.19 \times 0.52 \times 1.0 = 0.0988$, that for $117 \to 50 \to 113$ is $0.21 \times 0.49 = 0.1029$. These probabilities summed over all signal paths led to an absorption probability $\wp_{pMN}^{117}$ of 1, indicating that state 117 belongs exclusively to the basin of attraction of pMN. Such sub-graphs of partial STG starting from a state and culminating in different FPs were

used to identify the corresponding absorption probabilities. In Supplementary Figure 9A, we present an example of state 00001110 absorbing into FPs $p0$ and $p3$ along with the one-step transition probabilities.

In order to enable placing the states into the basin of attraction of the five FPs (Supplementary Table 5), using $\mathcal{M}^{q_l}$, we estimated the absorption probability of each the states to absorb into these fixed points. While some states such as 00001110 could absorb into FPs $p0$ and $p3$ with equal probabilities, many others such as state 00000000 fall into the basin of attraction of all five FPs with a finite probability (Supplementary Figure 8B). Thus, all states in the state space of the STG can be classified according to their absorption probability in reaching the five FPs. Venn diagram (Supplementary Figure 8C) shows the statistics of the states absorbing into different FPs and that ~18% of the states have the ability to reach all five FPs. Based on these absorption probabilities, we estimated the steady-state probability of the network's ability to settle into the five FPs, specified next to every phenotype in Supplementary Figure 8C. A comparison with those computed using the complete state transition matrix shows that BM-ProSPR algorithm enables accurate estimation of the steady-state probabilities using a significantly smaller number of Boolean dynamics simulations (Supplementary Figure 9B). This clearly demonstrates that the proposed algorithm can reliably lead to identifying the minimum number of permutations q_l required to estimate $\mathcal{M}^{q_l}$ in order to find the steady-state probabilities of different FPs.

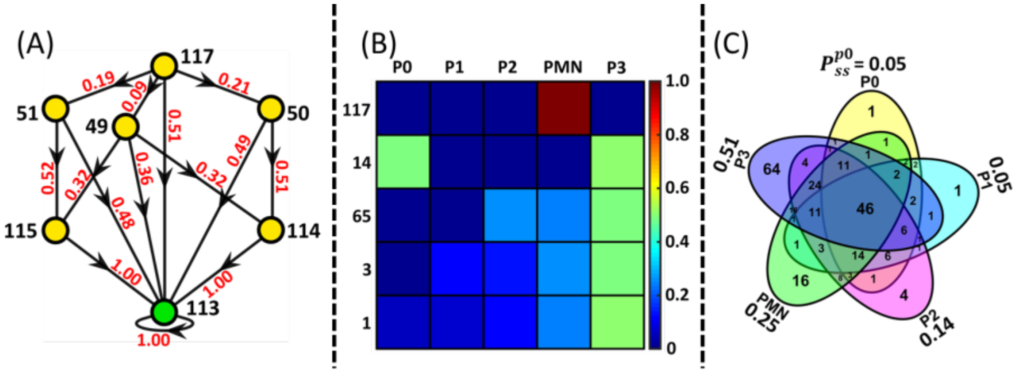

Supplementary Figure 8: FP reachability. (A) Signaling flow path to reach FP: Various paths are shown from a state 01100011 (state ID 117) to reach the fixed point state ID 113. States with yellow color are transient ones and green represents a FP. Transition probability for the one-step state transitions are shown next to the directed interaction in the STG. (B) Reachability to different cell types (phenotypes) from a few states along

with the corresponding absorption probabilities. (C) Venn diagram showing sharing of states between the five FPs. The steady-state probability of network's ability to settle in these five FP is mentioned next to ellipse.

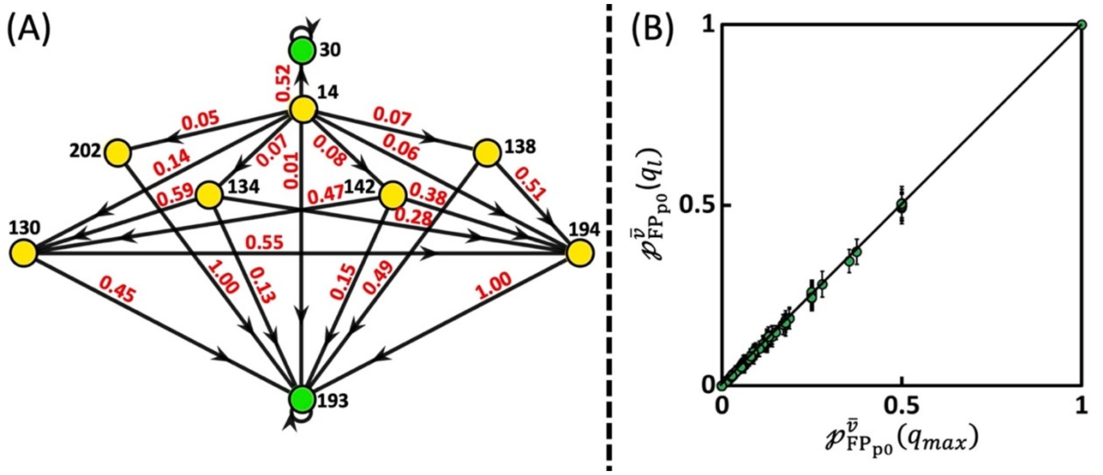

Supplementary Figure 9: Multiple FP reachability. (A) All signaling flow paths from state 00001110 (state ID 14) that can reach to two different FPs, that is, $p0$ (state 00101110, ID 30) and$p3$ (state 01010000, ID 193). The values next to the arrows capture the corresponding one-step state transition probability **(B)** Comparison of absorption probabilities computed from reliable partial STG with q_l permutations to those of complete STG. Error bars capture the standard deviation from the mean across 50 STG reconstructions.

Supplementary Note 3.5: Optimal error tolerance

In order to assess the effect of variation in the error tolerance, which affects the minimum number of permutations, on the extent to which the absorption probabilities computed from the partial STG matched those from the complete STG. For the sake of simplicity, we assumed same value for both error tolerances in Eqs 5 and 6 in the main text, that is, $\epsilon_1 = \epsilon_2 = \epsilon$. As a measure of assessment, for every tolerance value chose, we implemented the BM-ProSPR algorithm in the 8-node network and estimated the symmetric Kulback-Leibler Divergence given by

$$\mathbb{KL}\left(\mathbb{A}_{q_l}, \mathbb{A}_{q_{max}}\right) = \frac{1}{2}\left(\sum -\mathbb{A}_{q_l} \ln\left(\frac{\mathbb{A}_{q_l}}{\mathbb{A}_{q_{max}}}\right) + \sum -\mathbb{A}_{q_{max}} \ln\left(\frac{\mathbb{A}_{q_{max}}}{\mathbb{A}_{q_l}}\right)\right) \quad (4)$$

where $\mathbb{A}_{q_l}(\wp^{\bar{v}}_{\mathrm{FP_i}})$ and $\mathbb{A}_{q_{max}}(\wp^{\bar{v}}_{\mathrm{FP_i}})$ are the distributions of the absorption probability (of reaching the fixed point $\mathrm{FP_i}$) computed from the partial STG at the minimum number of permutation q_l and from the complete STG, respectively. The dependence of $\mathbb{KL}(\mathbb{A}_{q_l}, \mathbb{A}_{q_{max}})$ on

ϵ is in Supplementary Figure 10. We then identified that ϵ after which the $\mathbb{KL}(\mathbb{A}_{q_l}, \mathbb{A}_{q_{max}})$ does not change appreciably as the optimal error tolerance to find the minimum number of permutations for arriving at a reliable partial STG. The minimum ϵ for the 8-node network considered is $1e^{-4}$.

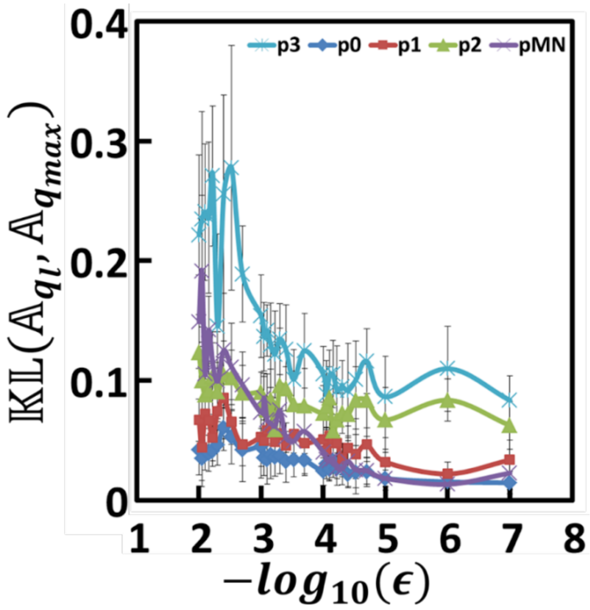

Supplementary Figure 10: Sensitivity analysis of the absorption probability distribution to reach the attractors of the 8-node network[51] with respect to the error tolerance adopted for the BM-ProSPR. Symmetric Kulback-Leibler divergence $\mathbb{K}(\mathbb{A}_{q_l}, \mathbb{A}_{q_{\max}})$ given by Eq. 4 (above) is used to compare the extent to which the absorption probability distribution computed at a certain tolerance mimics that of the complete STG. Error bars capture the mean $\pm$ standard deviation across 5 STG reconstructions.

We next assessed the sensitivity of the steady-state probability with respect to the error tolerance set. We assumed $\epsilon_1 = \epsilon_2 = \epsilon$. The steady-state probability achieved for reaching pro-survival attractor by assuming $\epsilon = 1 \times 10^{-3}$, 1×10^{-4}, and 1×10^{-5} are 0.8125, 0.8154, 0.8114. Since over three orders of magnitude the steady-state probability remained unchanged, we assumed $\epsilon = 2 \times 10^{-4}$.

Supplementary Note 4: Boolean dynamics of TNFR1 signalling network

__**Supplementary Note 4.1**: Minimum number of permutations for computing state transition matrix__

Temporality measure and the fraction of discordant pairs as a function of the number of permutations for the three input conditions, *viz.*, no stimulation (basal), TNFα stimulation, FasL stimulation are in the first three columns of Supplementary Figure 11. The minimum number of permutations required to reliably estimate the state transition matrix corresponding to the partial STG of the TNFR1 network Boolean model (Fig. 2, main text) for these cases are in Supplementary Table 6. Note that for the Basal, TNFα, and FasL cases, all 50 reconstructions resulted in the same minimum number of permutations. This is possibly due to the topology of the STG saturating faster leading to very marginal changes in the evolution of the STG when new permutations are introduced, as captured by Temporality measure converging slower than the PageRank for these cases (Supplementary Figure 11, panels I and II).

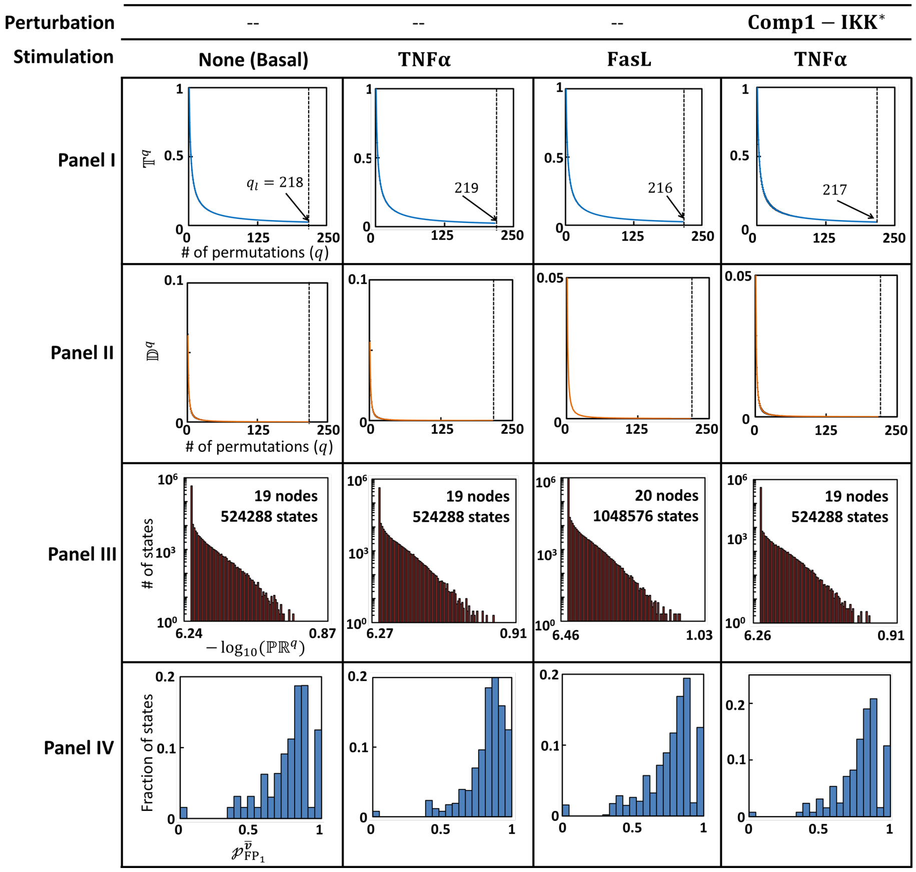

Supplementary Figure 11: Metrics and pro-survival absorption probabilities for the three input and perturbation conditions. Effect of permutations on the (Panel I)

Temporality measure $\mathbb{T}$ and (Panel II) Fraction of discordant pairs $\mathbb{D}$. (Panel III) PageRank distribution and (Panel IV) absorption probability distribution for reaching pro-survival phenotype. Error bars in panels I and II capture the standard deviation from the corresponding ensemble average.

For the case of perturbed networks, TNFR1^{Δ}, stimulated with TNFα, we first identified the attractors using the procedure in Supplementary Note 2.1. The attractors of these perturbed networks are in Supplementary Table 7. Dependence of the temporality measure and the fraction of discordant pairs on q are in Supplementary Figure 11, column 4. Note that knocking off of $\text{Comp1} - \text{IKK}^*$ does not alter the basal response as Comp1 always takes a Boolean value of 0 in the absence of stimulation.

Supplementary Table 6: BM-ProSPR predicted minimum number of permutations q_l for reliable estimation of the partial state transition matrix for different input and perturbation conditions on the TNFR1 network.

Input Conditions	q_l
Basal	218
TNFα	219
FasL	216
TNFα + Takinib	217

Supplementary Table 7: FPs reached by activated TNFR1 signaling network after introduction of perturbations. FP_1 and FP_2, respectively refers to pro-survival and apoptotic phenotypes. Procedure described in Supplementary Note 2.1 was used finding the FPs.

ID	Entity	TNFR1^{Δ}	
		$\bar{v}_{\text{FP}_1}$	$\bar{v}_{\text{FP}_2}$
1	TRADD	1	1
2	RIP	1	1
3	TRAF2	1	1
4	FADD	1	1
5	$\text{p} - 14 - 3 - 3$	1	1
6	c8	1	1
7	IKK	1	1
8	cIAP1/2	1	1
9	PARP	1	1
10	TNFα	1	1
11	FasL	0	0
12	TNFR1	1	1
13	Comp1	1	1

14	Comp2	1	1
15	$c8^* - \text{Comp2}$	1	1
16	Fas	0	0
17	DISC	0	0
18	$c8^* - \text{DISC}$	0	0
19	$c8^*$	1	1
20	cIAP1/2^*	1	1
21	$c3^* - p20$	1	1
22	$c3^* - p17$	0	1
23	PARP^*	1	0
24	CAD	0	1
25	PI3K	1	0
26	PKB	1	0
27	Raf1	1	0
28	$\text{Bad} - 14 - 3 - 3$	1	0
29	$\text{BCL} - \text{xL}$	1	0
30	Bax	0	1
31	smac	0	1
32	$\text{I}\kappa\text{B}^*$	0	1
33	IKK^*	1	0
34	$\text{Comp1} - \text{IKK}^*$	0	0
35	FLIP	1	0
36	XIAP	1	0
37	A20	1	0
38	$\text{BCL} - 2$	1	0
39	$\text{NF}\kappa\text{B}$	1	0
40	Apoptosis	0	1

Supplementary Note 4.2: BD simulations on a Random network model

We constructed a random network using configuration model.[52] In order to correspond to the TNFR1 network (Fig. 2, main text), we used the in-degree distribution, sign distribution and logics distribution while construction a random network. We wrote the Boolean function for the nodes in the constructed network and simulated the model using BM-ProSPR algorithm (Supplementary Table 8). Note that the simulations were performed only on the dynamically varying nodes, while value of rest of the entities were fixed using pLSSA (Methods, main text). The error tolerance used was same as that specified for the TNFR1 network (Fig. 2, main text). The algorithm showed that the minimum number of permutations needed is equal to 216 ± 5. We note that the random model did not exhibit any fixed point attractor and therefore the steady-state probability of reaching the FPs could not computed. Since the minimum number of

permutations needed to reliably capture the state transition matrix is significantly smaller compared to the maximum possible permutations, the algorithm BM-ProSPR could be implemented on any general network.

Supplementary Table 8: Boolean functions corresponding to the nodes in the random network constructed using Configuration model. &, | and $\sim$ operators respectively capture AND, OR and NOT operations.

Boolean function name	Boolean function
$f_{Node(1)}$	$Node(1)$
$f_{Node(2)}$	$Node(5)$ & $\sim Node(7)$
$f_{Node(3)}$	$Node(2)$
$f_{Node(4)}$	$Node(8)$
$f_{Node(5)}$	$\sim Node(10)$ & $Node(17)$
$f_{Node(6)}$	$Node(8)$
$f_{Node(7)}$	$Node(14)$
$f_{Node(8)}$	$\sim Node(7)$
$f_{Node(9)}$	$Node(7)$
$f_{Node(10)}$	$\sim Node(16)$
$f_{Node(11)}$	$Node(11)$
$f_{Node(12)}$	$Node(6)$ & $\sim Node(7)$
$f_{Node(13)}$	$\sim Node(1)$ | $\sim Node(7)$ | $\sim Node(13)$
$f_{Node(14)}$	$\sim Node(5)$
$f_{Node(15)}$	$\sim Node(4)$ & $\sim Node(9)$ | ($Node(14)$ & $\sim Node(18)$)
$f_{Node(16)}$	$Node(8)$ & $\sim Node(15)$
$f_{Node(17)}$	$Node(1)$ & $\sim Node(16)$
$f_{Node(18)}$	$Node(7)$
$f_{Node(19)}$	$Node(12)$
$f_{Node(20)}$	$\sim Node(3)$ | $\sim Node(4)$

Supplementary Note 4.3: Illustration of computing the conditional probability of a node being active in a path in the STG

Consider a start state s_1 with $\overline{\boldsymbol{v}}_{s_1}$ containing the corresponding Boolean values. Let the node (n) be inactive, that is, its Boolean value v_n in $\overline{\boldsymbol{v}}_{s_1}$ is 0. As an illustration, consider a few signal flow paths from s_1 traversing through different states and culminating in either a FP (say FP_1 or FP_2) or in states belong to the strongly connected component of the STG (Supplementary Figure 12A). We define pseudo-time points $(t = 1, 2, 3, \dots, \infty)$ placed sequentially at the states in a signal flow path starting from s_1. The states appearing in various signal flow paths from s_1 are aligned as per the pseudo-time points, as illustrated in Supplementary Figure 12. For every pseudo-time point, using Eqs 8 and 9, main text, the connectivity matrix $(\mathbb{C})$ is populated, a few columns of which corresponding to the signal flow paths in Supplementary Figure 12A are shown in Supplementary Figures 12B. Using the $\mathbb{C}$ and the state transition matrix at the minimum number of permutations, the signal flow path matrix $\mathbb{P}$ and the conditional probability $\mathbb{E}_N|_{s_i}$ are estimated using Eqs 10 to 13, main text.

(A)

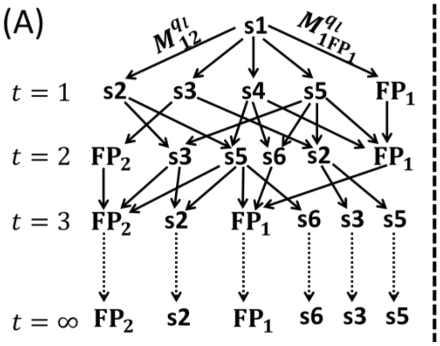

(B)

	s1	s2	s3	s4	s5	s6	FP_1	FP_2
$t = 1$	0	1	1	1	1	0	1	0
$t = 2$	0	1	1	0	1	1	1	1
$t = 3$	0	1	1	0	1	1	1	1
⋮	⋮	⋮	⋮	⋮	⋮	⋮	⋮	⋮
$t = \infty$	0	1	1	0	1	1	1	1

Supplementary Figure 12: (A) An illustration of alignment of the signal flow paths in STG as per the pseudo-time steps (t). s_1 to s_6 represent transient states, $M_{12}^{q_l}$ is the one-step state transition probability to reach s_2 from s_1, and FP_1 and FP_2 are the fixed points. (B) Connectivity matrix $\mathbb{C}$.

Supplementary Note 4.4: Analysis of NFκB, PI3K and $\text{c3}^* - \text{p17}$ transients at the single-cell level

From the single-cell level dynamics $(\mathbb{E})$ of NFκB, IκB*,PI3K and $\text{c3}^* - \text{p17}$ being active estimated from the Boolean dynamic simulations (Fig. 5A-D, main text), we culled out those transients exhibiting a qualitative undulation behaviour similar to that observed in experimentally measured (Fig. 5E-H, main text). The number of states that satisfied this comparison for NFκB, IκB , PI3K and $\text{c3}^* - \text{p17}$ are 58031, 59186, 54896, and 60435

respectively (Supplementary Figure 13A). This shows that the dynamics of the significant fraction of the states simulated by the Boolean model of the TNFR1 network mimics experimentally measured transients.

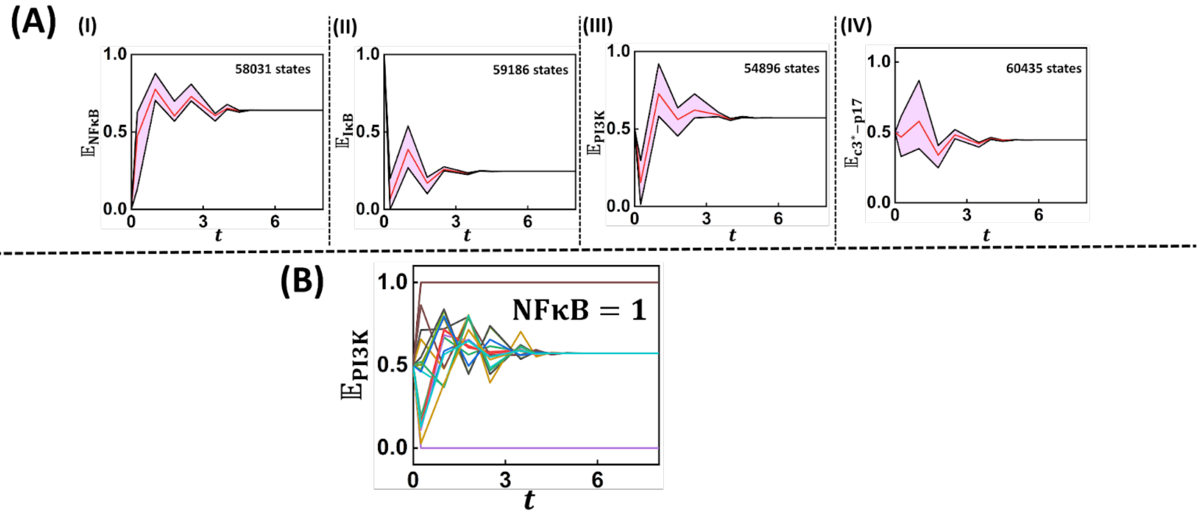

Supplementary Figure 13: (A) Dynamics of the conditional probability of (I) NFκB, (II) $I\kappa B^*$ (III) PI3K and (IV) $c3^* - p17$ being active similar to those measured by experimentation. Number of states that matched the similarity condition for each of these three nodes is specified inside the corresponding figure. (B) The trajectories of PI3K being active from 20 randomly chosen states, in which NFκB is active, corresponding to the dynamics of PI3K at single-cell level.

Supplementary Note 4.5: Steady-state probability of FPs exhibiting phenotype response

TNFα stimulation led to a ~7% increase in the steady-state probability (over basal) in the network culminating into a pro-survival phenotype (Supplementary Figure 14). Note that the anti-apoptotic phenotype (FP_3), one of the basal responses having a $P_{ss}^{FP_3} = 0.23$, is absent for the TNFα stimulation case. Under no stimulation conditions, when NFκB is not activated, positive feedback loop involving $c3^* - p20$, $c3^* - p17$ and smac permits $c3^* - p17$ being either active (1) or inactive (0) leading respectively to apoptotic or anti-apoptotic phenotypes. However, when stimulated with TNFα, $c3^* - p17$ can only take an active form in the absence of NFκB, indicating that the anti-apoptotic phenotype is absent in the stimulated TNFR1 network. An independent activation of $c3^* - p20$ by FasL (positive control) suggests that anti-apoptotic

phenotype is not found when the positive feedback loop is activated under stimulatory conditions.

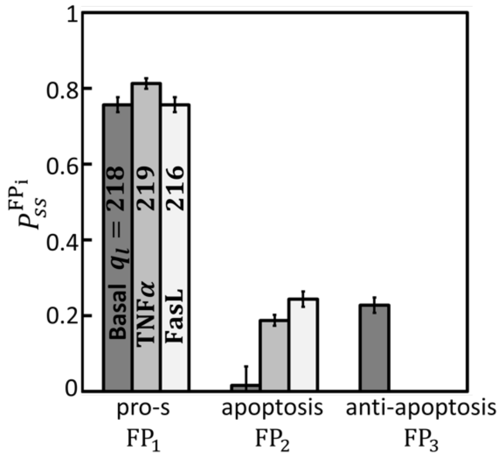

Supplementary Figure 14: Steady-state probabilities of TNFR1 network settling into different phenotypes (FPs), *viz.*, pro-survival (pro-s), apoptosis (Apop) and anti-apoptotic (anti-apop) under Basal, TNFα and FasL. Error bars capture mean $\pm$ standard deviation across 50 STG reconstructions.

<u>**Supplementary Note 4.6**: Illustration of a shift in signal flow paths achieved by inhibiting $\mathrm{Comp1-IKK^*}$</u>

Consider a signal flow path originating from state ID 385 (Supplementary Figure 15; Supplementary Table 9) along with a few one-step state transitions leading to states 132726, 394305, 329475, etc. in case of (unperturbed) TNFR1 network (Fig. 2). Note that $\mathrm{Comp1-IKK^*}$ is a dynamically varying entity taking a value of 1 and that state 328771 belongs exclusively to apoptosis phenotype $\mathrm{FP_2}$ (Supplementary Table 9). However, upon inhibiting $\mathrm{Comp1-IKK^*}$, the modified network ($\mathrm{TNFR1^{\Delta}}$) causes a change in the transition probability of reaching state 394497 from 385 as compared to that achieved under the normal unperturbed conditions (Supplementary Figure 15) but continues to be exclusively in the basin of attraction of the apoptotic phenotype ($\mathrm{FP_2}$) (Supplementary Table 9). On the other hand, under TAK1 inhibitory conditions, several new transitions have emerged, for e.g., those from state 385→394497, 385→460545 having a transition probability of 0.004 and 0.008, respectively. Note that under perturbed conditions, state 385 itself have a higher absorption probability to reach apoptosis as compared to that achieved under unperturbed case (Supplementary Table 9). This demonstrates that the TAK1 perturbation has indeed caused a shift in the signal flow paths towards apoptosis.

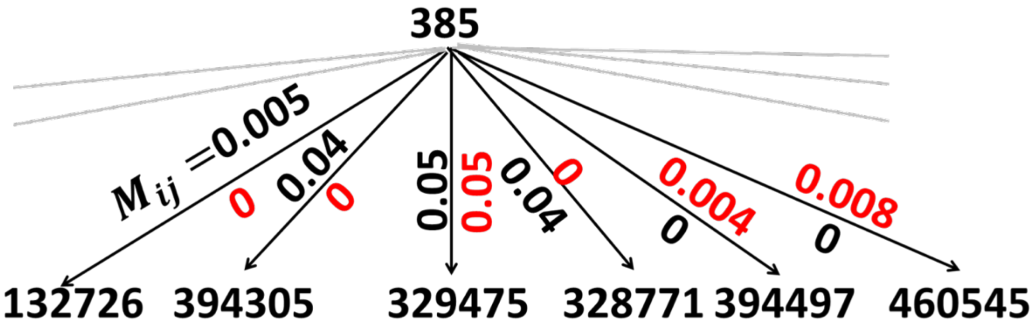

Supplementary Figure 15: An illustration of a few one-step state transitions from state ID-385 along with the transition probabilities in case of TNFα stimulated TNFR1 (black) and TNFR1$^{\Delta}$ network (red). The Boolean values of different nodes in the network along with the absorption probabilities are in Supplementary Table 9.

Supplementary Table 9: Boolean values and the absorption probability to reach apoptosis FP ($\wp^{\bar{v}}_{\mathrm{FP}_2}$) for the states in the signal flow paths of TNFR1 and $TNFR1^{\Delta}$ network in Supplementary Figure 15.

**State ID**	**State**	$\wp^{\bar{v}}_{\mathrm{FP}_2}$ **in TNFR1**	$\wp^{\bar{v}}_{\mathrm{FP}_2}$ **in TNFR1$^{\Delta}$**
385	1111111110111100011100000000000110000000	0.46	0.63
132726	1111111110111100011101000000011001110110	0	0
394305	1111111110111100011111000000010001000000	0.26	0
FP_2	1111111110111100011110100000011100000001	1	1
328771	1111111110111100011110100000010001000001	0.27	0
394497	1111111110111100011111000000010100000000	1	1
460545	1111111110111100011111100000011100000000	1	1

<u>**Supplementary Note 4.7**: Apoptotic response observed in TAK1 inhibited U937 and Jurkat-T cells</u>

We used Takinib as a TAK1 inhibitor[53] We measured the apoptotic levels in the Takinib pre-treated U937 and Jurkat-T cells exposed to 100 ng/ml TNFα stimulation using Annexin V-PI assay. Additionally, as a control, we also performed Annexin-PI assay on cells exposed only to Takinib. The sample time for the TAK1 inhibitory conditions is the same as that adopted in Supplementary Note 1.1 for uninhibited cells. Apoptosis levels were measured at an ensemble-level (Methods, main text). For every experimental condition and time point, three replicates were used. Pro-survival and apoptotic response obtained for the basal as well as the

case of cells treated only with Takinib are in Supplementary Figure 16. Takinib had no effect on the overall viability of both U937 and Jurkat-T cells. Pro-survival and apoptotic response obtained in U937 cells after 24 hrs and those in Jurkat-T cells after 18 hrs, both with TNFα stimulation are respectively shown in Supplementary Figures 17A and 17C. Fraction of cells present in survival state were ~0.48. Moreover, effect of the TNFα exposure time on the apoptosis fold-change for both cell lines is presented in Supplementary Figure 17B and 17D. Model analysis reflecting these observations are in Supplementary Note 1.4.

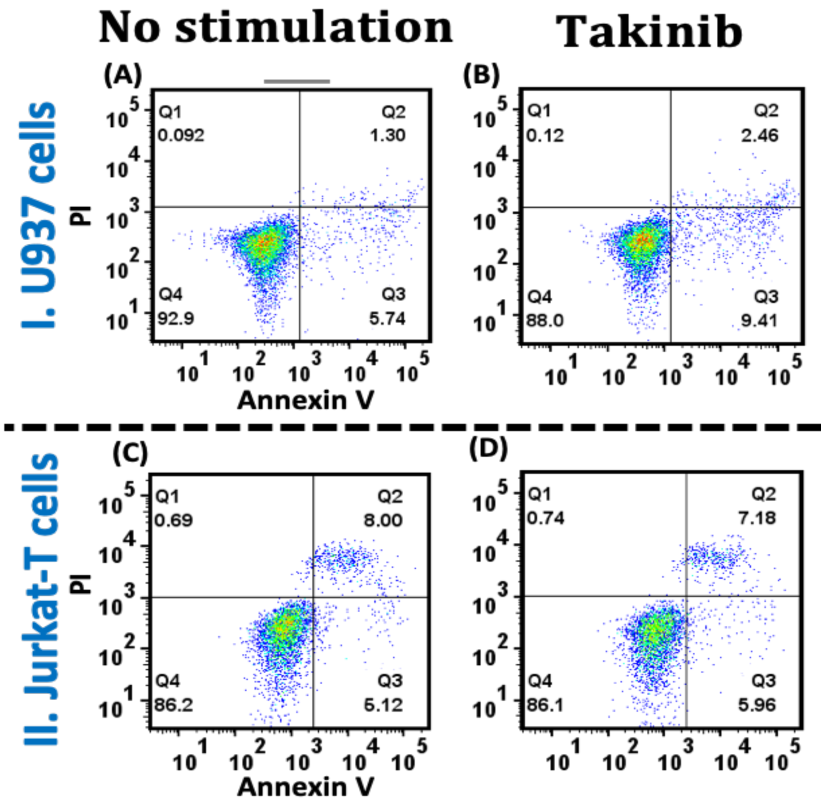

Supplementary Figure 16: Apoptosis response in TAK1 inhibited U937 (Panel I) and Jurkat-T cells (Panel II). (A) and (C) are the four-quadrant plots capturing different U937 and Jurkat-T cell states following no TNFα stimulation for 24 and 18 hrs, respectively. The four-quadrants specify populations as indicated in Supplementary Figure 1. (B) and (D) captures the effect of exposure time on FC_A (defined in Supplementary Note 1) following TAK1 inhibited U937 and Jurkat-T cells, respectively pre-treated with 20 μM Takinib[53]

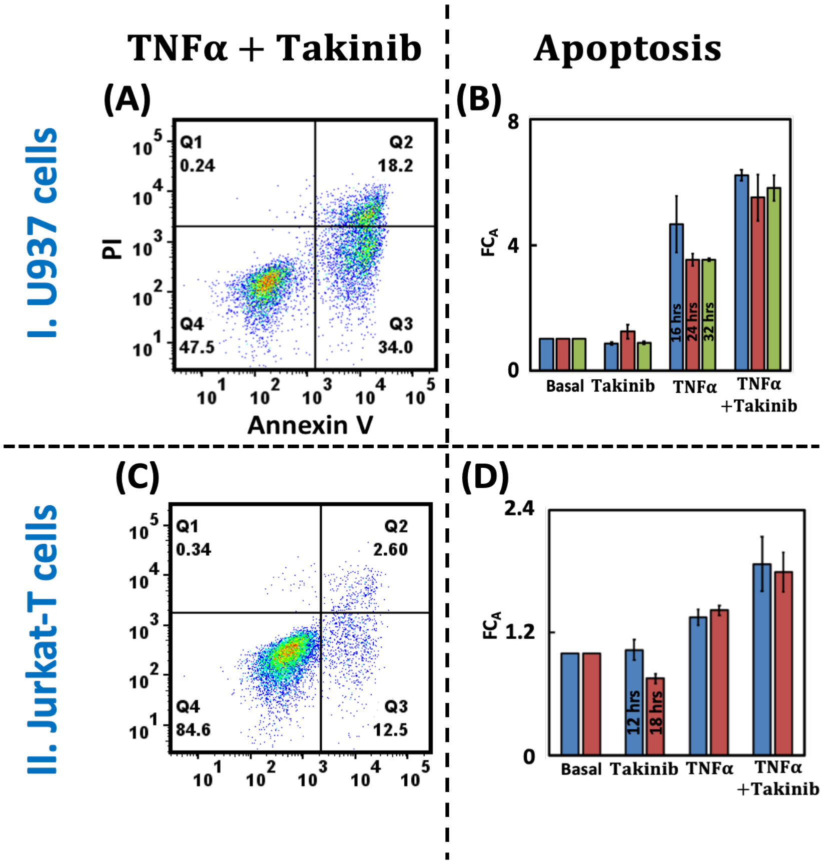

Supplementary Figure 17: TNFα mediated pro-survival and apoptosis response in TAK1 inhibited U937 (Panel I) and Jurkat-T cells (Panel II). (A) and (C) are the four-quadrant plot capturing different U937 and Jurkat-T cell states following 100 ng/ml TNFα stimulation after a pre-treatment with 20 µM Takinib[53] for 24 and 18 hrs, respectively. The four-quadrants specify populations as indicated in Supplementary Figure 1. (B) and (D) captures the effect of exposure time on FC_A following TNFα stimulation in TAK1 inhibited U937 and Jurkat-T cells, respectively. Error bars capture the mean ± standard deviation across three independent replicates.

<u>**Supplementary Note 4.8**: Maximum pseudo-time points in the signal flow paths of the STG</u>

The connectivity matrix $\mathbb{C}$ (Eqs 8 and 9, main text), along with the path matrix $\mathbb{P}$ (Eqs 10 and 11, main text) and the conditional probability $\mathbb{E}$ (Eqs 12 and 13, main text) were assembled for two instances of the state transition graph constructions. Histogram of the number of states for which $\mathbb{C}$ saturates at a certain maximum pseudo-time point, for each of these STG instances, are in Supplementary Figure 18. The maximum number of pseudo-time points needed to traverse through all the signal flow paths in the STG hovers around 12.

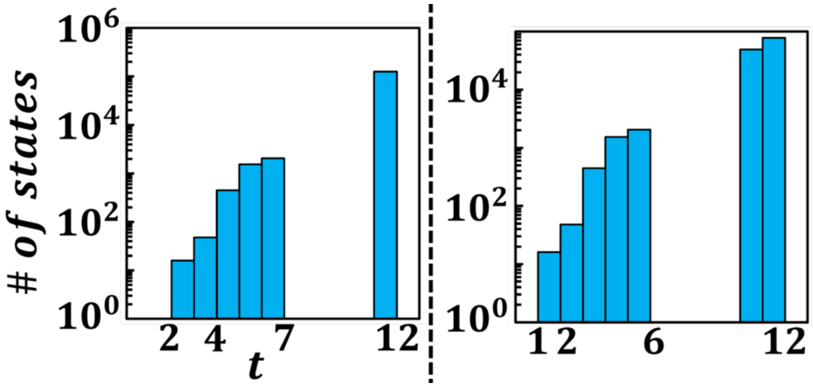

Supplementary Figure 18: The distribution of the start states requiring a certain maximum pseudo-time points (t) in the connectivity matrix $\mathbb{C}$ for two instances of the state transition graph constructions.

Supplementary References

1. Wajant H, Pfizenmaier K, Scheurich P. Tumor necrosis factor signaling. *Cell Death & Differentiation* **10,** 45-65 (2003)
2. Michieu O, Tshopp. Induction of TNF receptor-I mediated apoptosis via two sequential ssignaling complexes. *Cell* **114,** 181-190 (2003)
3. Wajant H, Scheurich P. TNFR1-induced activation of the classical NF-κB pathway. *FEBS J* **278,** 862-876 (2011)
4. Wang C, *et al*. TAK1 is a ubiquitin-dependent kinase of MKK and IKK. *Nature* **412**, 346-351 (2001)
5. Dondelinger Y, *et al*. NF-κB-independent role of IKKα/IKKβ in preventing RIPK1 kinase-dependent apoptotic and necroptotic cell death during TNF signaling. *Molecular cell* **60**, 63-76 (2015)
6. Geng J, *et al*. Regulation of RIPK1 activation by TAK1-mediated phosphorylation dictates apoptosis and necroptosis. Nature communications 8:359 (2017)
7. Dondelinger Y, *et al*. MK2 phosphorylation of RIPK1 regulates TNF-mediated cell death. Nature cell biology **19**,1237-1247 (2017)
8. Hayden MS, Ghosh S. Signaling to NF-κB. *Genes & development* **18**,2195-2224 (2004)
9. Zhang Z, *et al*. An antiapoptotic Bcl-2 family protein index predicts the response of leukaemic cells to the pan-Bcl-2 inhibitor S1. *British journal of cancer* **108**,1870-8 (2013)
10. Lickliter JD, *et al*. Small-molecule Bcl-2 inhibitors sensitise tumour cells to immune-mediated destruction. *British journal of cancer* **96**, 600-8 (2007)
11. de Moissac, Mustapha S, Greenberg AH, Kirshenbaum LA. Bcl-2 activates the transcription factor NFkappaB through the degradation of the cytoplasmic inhibitor IkappaBalpha. *The Journal of biological chemistry* **273**, 37 (1998)
12. de Moissac D, Zheng H, Kirshenbaum LA. Linkage of the BH4 domain of Bcl-2 and the nuclear factor κB signaling pathway for suppression of apoptosis. *Journal of Biological Chemistry* **274**, 29505-9 (1999)

13. Popgeorgiev N, Jabbour L, Gillet G. Subcellular localization and dynamics of the Bcl-2 family of proteins. *Frontiers in cell and developmental biology* **13**, 6:13 (2018)
14. Vasudevan KM, Gurumurthy S, Rangnekar VM. Suppression of PTEN Expression by NF-B Prevents Apoptosis. *Molecular and cell biology* **24,** 1007–1021 (2004)
15. Carracedo A, Pandolfi PP. The PTEN–PI3K pathway: of feedbacks and cross-talks. *Oncogene* **27,** 5527-5541 (2008)
16. McCubrey JA, *et al.* Roles of the Raf/MEK/ERK pathway in cell growth, malignant transformation and drug resistance. *Biochimica et Biophysica* **1773,** 1263-1284 (2007)
17. Arsura M, Mercurio F, Oliver AL, *Thorgeirsson SS, Sonenshein GE.* Role of the IκB kinase complex in oncogenic Ras-and Raf-mediated transformation of rat liver epithelial cells. *Molecular and Cellular Biology* **20,** 5381-5391 (2000)
18. Hutti JE, Turk BE, Asara JM, Cantley LC, Abbott DW. IκB kinase β phosphorylates the K63 deubiquitinase A20 to cause feedback inhibition of the NF-κB pathway. *Molecular and cellular biology* **27**, 7451-7461 (2007)
19. Shembade N, Ma A, Harhaj EW. Inhibition of NF-κB signaling by A20 through disruption of ubiquitin enzyme complexes. *Science* **327**, 1135-1139 (2010)
20. Hunter AM, LaCasse EC, Korneluk RG. The inhibitors of apoptosis (IAPs) as cancer targets. *Apoptosis* **12,** 543:1568 (2007)
21. Hemmings BA, Restuccia DF. Pi3k-pkb/akt pathway. *Cold Spring Harbor perspectives in biology* **4,** a011189 (2012)
22. Rath PC, Aggarwal BB. TNF-induced signaling in apoptosis. *J Clin Immunol* **19,** 350-364 (1999)
23. Wang L, Du F, Wang X. TNF-alpha induces two distinct caspase-8 activation pathways. *Cell* **133,** 693-703 (2008)
24. Wilson NS, Dixit V, Ashkenazi A. Death receptor signal transducers: nodes of coordination in immune signaling networks. *Nat Immunol* **10,** 348-355 (2009)
25. Deveraux QL, *et al.* IAPs block apoptotic events induced by caspase-8 and cytochromec by direct inhibition of distinct caspases. *EMBO J* **17,** 2215-2223 (1998)
26. Bratton SB, Lewis J, Butterworth M, Duckett CS, Cohen *GM.* XIAP inhibition of caspase-3 preserves its association with the Apaf-1 apoptosome and prevents CD95- and Bax-induced apoptosis. *Cell death and differentiation* **9,** 881-892 (2002)
27. Wei MC, *et al.* Proapoptotic BAX and BAK: a requisite gateway to mitochondrial dysfunction and death. *Science* **292,** 727-730 (2001)
28. Tsuruta F, Masuyama N, Gotoh Y. The Phosphatidylinositol 3-Kinase (PI3K)-Akt Pathway Suppresses Bax Translocation to Mitochondria. *J of Biol chem* **277,** 14040-14047 (2002)
29. Peña-Blanco A, García-Sáez AJ. Bax, Bak and beyond—mitochondrial performance in apoptosis. *FEBS J* **285,** 416-431 (2018)
30. Harlin H, Reffey SB, Duckett CS, Lindsten T, Thompson CB. Charaterization of XIAP-deficient mice. *Molecullar Cell Biology* **21,** 3604-3608 (2001)
31. Yang S, Thor AD, Edgerton S, Yang X. Caspase-3 mediated feedback activation of apical caspases in doxorubicin and TNF-α induced apoptosis. *Apoptosis* **11**:1987-1997 (2006)
32. Fujita E, Egashira J, Urase K, Fujita E, Egashira J, Urase K. Caspase 9 processing by caspase-3 via a feedback amplicaion loop *in vivo*. *Nature* **8,** 335-344 (2001)

33. O'Connor CL, *et al.* Intracellular signaling dynamics during apoptosis execution in the presence or absence of X-linked-inhibitor -of-apoptosis protein. *Biochimica et biophysica acta-molecular cell research* **1783**, 1903-1913 (2008)
34. Fujita N, Nagahashi A, Nagashima K, Rokudai S, Tsuruo T. Acceleration of apoptotic cell death after the cleavage of Bcl-XL protein by caspase-3-like proteases. *Oncogene* **17(10)**, 1295-304 (1998)
35. Fadeel B, *et al*. Cleavage of Bcl-2 is an early event in chemotherapy-induced apoptosis of human myeloid leukemia cells. *Leukemia*. **13(5)**, 719-28 (1999)
36. Cryns v, Yuan J. Proteases to die for. *Genes and development* **12,** 1551-1570 (1998)
37. Lavrik IN, Krammer PH. Regulation of CD95/Fas signaling at the DISC. *Cell Death & Differentiation* **19,** 36-41 (2012)
38. Elzey BD, *et al*. Regulation of Fas ligand-induced apoptosis by TNF. *The Journal of Immunology* **167,** 3049-3056 (2001)
39. Hsu, H , Xiong J, Goeddel DV. The TNF receptor 1-associated protein TRADD signals cell death and NF-kappa B activation. *Cell* **81**, 495-504 (1995)
40. Hsu H, Huang J, Shu HB, *et al.* TNF-dependent recruitment of the protein kinase RIP to the TNF receptor-1 signaling complex. *Immunity* **4**, 387-396 (1996)
41. Song HY, Donner DB. Association of a RING finger protein with the cytoplasmic domain of the human type-2 tumour necrosis factor receptor. *Biochemical Journal* **309**, 825-829 (1995)
42. Kim PK, Dutra AS, Chandrasekharappa SC, Puck JM. Genomic structure and mapping of human FADD, an intracellular mediator of lymphocyte apoptosis. *Journal of immunology* **157**, 5461-5466 (1996)
43. Darling DL, Yingling J, Wynshaw-Boris A. Role of 14–3–3 proteins in eukaryoticsignaling and development. *Current topics in developmental biology* **68**, 281-315 (2005)
44. Gryko M, Łukaszewicz-Zając M, Guzińska-Ustymowicz K, K Kucharewicz M, Mroczko B. The caspase-8 and procaspase-3 expression in gastric cancer and non-cancer mucosa in relation to clinico-morphological factors and some apoptosis-associated proteins. *Advances in Medical Sciences* **68**, 94-100 (2023)
45. Mahoney DJ, *et al*. Both cIAP1 and cIAP2 regulate TNFα-mediated NF-κB activation. *Proceedings of the National Academy of Sciences* **105**, 11778-11783 (2008)
46. Amé JC, *et al*. PARP-2, A novel mammalian DNA damage-dependent poly (ADP-ribose) polymerase. *Journal of Biological Chemistry* **274**, 17860-17868 (1999)
47. Klamt S, Rodriguez JS, Gilles ED. Structural and functional analysis of cellular networks with CellNetAnalyzer, *BMC systems biology* **1,** 1-13 (2007)
48. Saadatpour A, *et al.* TP. Dynamical and structural analysis of a T-cell survival network identifies novel candidate therapeutic targets for large granular lymphocyte leukemia. *PLoS Comp Biol* **7**, e1002267 (2011)
49. Sen PK. Estimates of the regression coefficient based on Kendall's tau, *Journal of American statistical association* **63,** 1379-1389 (1968)
50. Golub, G. H. and Van Loan, C. F. *Matrix Computations*, 3rd ed. Baltimore, MD: Johns Hopkins, (1996)
51. Lovrics A, *et al*. Boolean modeling reveals new regulatory connections between transcription factors orchestrating the development of the Ventral spinal cord, *PLoS One* **9,** e111430 (2014)

52. Song Y. *Networks: An Introduction by MEJ Newman*: Oxford, UK: Oxford University Press. 250-251 (2013)
53. Totzke J, *et al*. Takinib, a selective TAK1 inhibitor, broadens the therapeutic efficacy of TNF-α inhibition for cancer and autoimmune disease. *Cell chemical biology* **24**, 1029-1039 (2017)
